# Meta-Analysis for the Global Prevalence of Foodborne Pathogens Exhibiting Antibiotic Resistance and Biofilm Formation

**DOI:** 10.3389/fmicb.2022.906490

**Published:** 2022-06-14

**Authors:** Qian Tao, Qian Wu, Zhaohuan Zhang, Jing Liu, Cuifang Tian, Zhenhua Huang, Pradeep K. Malakar, Yingjie Pan, Yong Zhao

**Affiliations:** ^1^College of Food Science and Technology, Shanghai Ocean University, Shanghai, China; ^2^Laboratory of Quality and Safety Risk Assessment for Aquatic Products on Storage and Preservation (Shanghai), Ministry of Agriculture and Rural Affairs, Shanghai, China; ^3^Shanghai Engineering Research Center of Aquatic-Product Processing and Preservation, Shanghai, China

**Keywords:** foodborne pathogens, antimicrobial resistance, biofilm, meta-analysis, global prevalence

## Abstract

Antimicrobial-resistant (AMR) foodborne bacteria causing bacterial infections pose a serious threat to human health. In addition, the ability of some of these bacteria to form biofilms increases the threat level as treatment options may become compromised. The extent of antibiotic resistance and biofilm formation among foodborne pathogens remain uncertain globally due to the lack of systematic reviews. We performed a meta-analysis on the global prevalence of foodborne pathogens exhibiting antibiotic resistance and biofilm formation using the methodology of a Cochrane review by accessing data from the China National Knowledge Infrastructure (CNKI), PubMed, and Web of Science databases between 2010 and 2020. A random effects model of dichotomous variables consisting of antibiotic class, sample source, and foodborne pathogens was completed using data from 332 studies in 36 countries. The results indicated AMR foodborne pathogens has become a worrisome global issue. The prevalence of AMR foodborne pathogens in food samples was greater than 10% and these foodborne pathogens were most resistant to β-lactamase antibiotics with *Bacillus cereus* being most resistant (94%). The prevalence of AMR foodborne pathogens in human clinical specimens was greater than 19%, and the resistance of these pathogens to the antibiotic class used in this research was high. Independently, the overall biofilm formation rate of foodborne pathogenic bacteria was 90% (95% CI, 68%–96%) and a direct linear relationship between biofilm formation ability and antibiotic resistance was not established. Future investigations should document both AMR and biofilm formation of the foodborne pathogen isolated in samples. The additional information could lead to alternative strategies to reduce the burden cause by AMR foodborne pathogens.

## Introduction

Infections caused antibiotic resistant bacteria are attributing to serious burdens for society (World Health Organization, 2021a) and where there are also threats to food safety and public health [Cania et al., 2018; FAO/OIE/WHO (Food and Agriculture Organization of the United Nations, World Organisation for Animal Health, World Health Organization), 2019]. It is estimated that 600 million people (almost 1 in 10) worldwide get sick from eating contaminated food and 420,000 die every year [World Health Organization (WHO), 2020a]. In the meanwhile, foodborne diseases cause huge economic losses in low- and middle-income countries, costing up to $110 billion annually [World Health Organization (WHO), 2020b]. Foodborne diseases are mainly caused by pathogenic microorganisms, such as *Escherichia coli*, *Staphylococcus aureus*, *Campylobacter* spp., *Salmonella* spp., and *Vibrio parahaemolyticus* [Kirk et al., 2015; World Health Organization (WHO), 2020a]. It cannot be ignored that the emergence of antibiotic resistance of foodborne pathogens has a serious impact on public health (Cania et al., 2018).

The use of antibiotics in the global food production system remains widespread and, concurrently, the development of antimicrobial resistance in humans, animals, plants, and the environment has accelerated [World Health Organization (WHO), 2021b]. There is increasing recognition that bacteria in natural ecosystems can transmit antibiotic resistance genes to humans and where the principal mode of transmission is human ingestion of food containing drug-resistant bacterial pathogens (Martínez, 2013; Hernando-Amado et al., 2019). In many clinical and food settings, foodborne pathogenic bacteria can adhere to biotic or abiotic surfaces to form biofilms, which can mitigate the action of antibiotics (Coughlan et al., 2016) and the results in decreased treatment efficacy (Guzman-Soto et al., 2021). According to the United States National Institutes of Health, biofilms mediate about 65% of human infections worldwide, and about 80% of chronic infections can be directly related to the formation of biofilms (Lebeaux et al., 2014; Melander and Melander, 2015). Moreover, a lot of research indicates that pathogens that produce biofilms are significantly more resistant to antibiotics and biocides than planktonic or free-living bacteria (Gebreyohannes et al., 2019; Karygianni et al., 2020).

Currently, foodborne pathogenic bacteria are monitored, globally, for resistance to important classes of antibiotics (Grundmann et al., 2011). And, a large number of studies of about drug resistance of foodborne pathogens and the ability to form biofilm has been published worldwide (see Supplementary Material). However, a systematic study of this data has yet to be reported. The aim of this meta-analysis is to systematically analyze (1) the global prevalence of antibiotic resistance and biofilm formation among foodborne pathogens and (2) the correlation between the antibiotic resistance and biofilm formation.

## Materials and Methods

### Search Strategy

A meta-analysis based on PRISMA guidelines (Moher, 2010) and Cochrane recommendations (Cumpston et al., 2019) were employed to investigate the antibiotic resistance and biofilm production of foodborne pathogens. Information from curated databases and academic websites, including of PubMed, Web of Sciences, and China National Knowledge Infrastructure (CNKI) were evaluated for the suitability to the meta-analysis. The following keywords: “antibiotic resistance,” “biofilm,” “biofilm formation,” “foodborne pathogenic bacteria,” “foodborne disease,” and “Food Poisoning” were used to search the information sources. In addition, the literature search was truncated, for articles published between 1 January 2010 and 31 December 2020, for six foodborne pathogen bacteria (*Bacillus cereus*, *Escherichia coli*, *Listeria monocytogenes*, *Salmonella* spp., *Staphylococcus aureus*, and *Vibrio parahaemolyticus*) and for ability to form biofilm. We also searched Google Scholar to ensure the comprehensiveness of the literature search, inclusive of literature from preprint repositories (bioRxiv and medRxiv).

### Selection Criteria

Literature was downloaded to EndNoteX9 and an initial screen removed duplicate information. Then eligible studies which met the following criteria was chosen: (1) pathogenic bacteria are common foodborne pathogens; (2) the pathogenic bacteria were isolates from food, patients with foodborne illness, or food practitioners with no search restrictions placed on human race, food type, sample size, and source; and (3) reported patterns of resistance of the foodborne pathogen and or the ability to form biofilm. Inclusion and exclusion of literature from the final analysis were performed, independently, by the authors, Qian Tao (TQ) and Qian Wu (WQ) by examining the titles, abstracts and full texts of the collected literature after the initial screen. This independent filtering of the literature minimized selection bias and a final selection was mediated through discussion or adjudication.

### Data Extraction and Quality Assessment

The authors, TQ and WQ, independently extracted data from the final selection by using a standardized format which included: the name of the first author, the published year, the period of sample collection, location, varieties of food, disease types, pathogenic bacteria investigated, number of isolates, method of antibiotic susceptibility testing, guideline used to interpret antimicrobial sensitivities, reported antibiotic sensitivities, number of antibiotic-resistant strains, number of multidrug-resistant strains, method of biofilm formation testing, biofilm types. If the information in a selection was unclear, an attempt was made to contact the author to verify the validity of the data. Merge tools and adapted version of the Joanna Briggs Institute (JBI) critical appraisal checklist (Moola et al., 2020) and the Newcastle-Ottawa quality-assessment scale (Wells, 2014) were also used for data assessment.

### Data Synthesis and Analysis

Stata (version 16) software was used for the statistical analysis of data from human and food studies. We used a forest plot to visualize effect sizes and 95% CI, where the estimated antibiotic resistance rate was a pooled according to the class of antibiotics used and type of bacteria. Preliminary analysis of data revealed that antibiotic resistance and biofilm formation were heterogeneous and a random-effects models was used to account for the heterogeneity across all the studies. The I^2^ statistic was used to quantify the heterogeneity across studies according to the following classes: a low level (less than 25%), a moderate level (25%–50%), and a high level (more than 75%; Higgins et al., 2003; IntHout et al., 2016).

Publication bias and subgroup analysis was completed by merging sample type and bacterial species. A funnel plot was used to show the extent of publication bias and a nonparametric scissor-complement analysis of publication bias was performed if bias existed. We conducted subgroup analysis from sample type, geographic location, date of sample collection (before 2010, 2011–2015, and 2016–2020) and susceptibility test.

## Results

### Study Selection, Characteristics, and Quality Assessment

**Figure 1:**
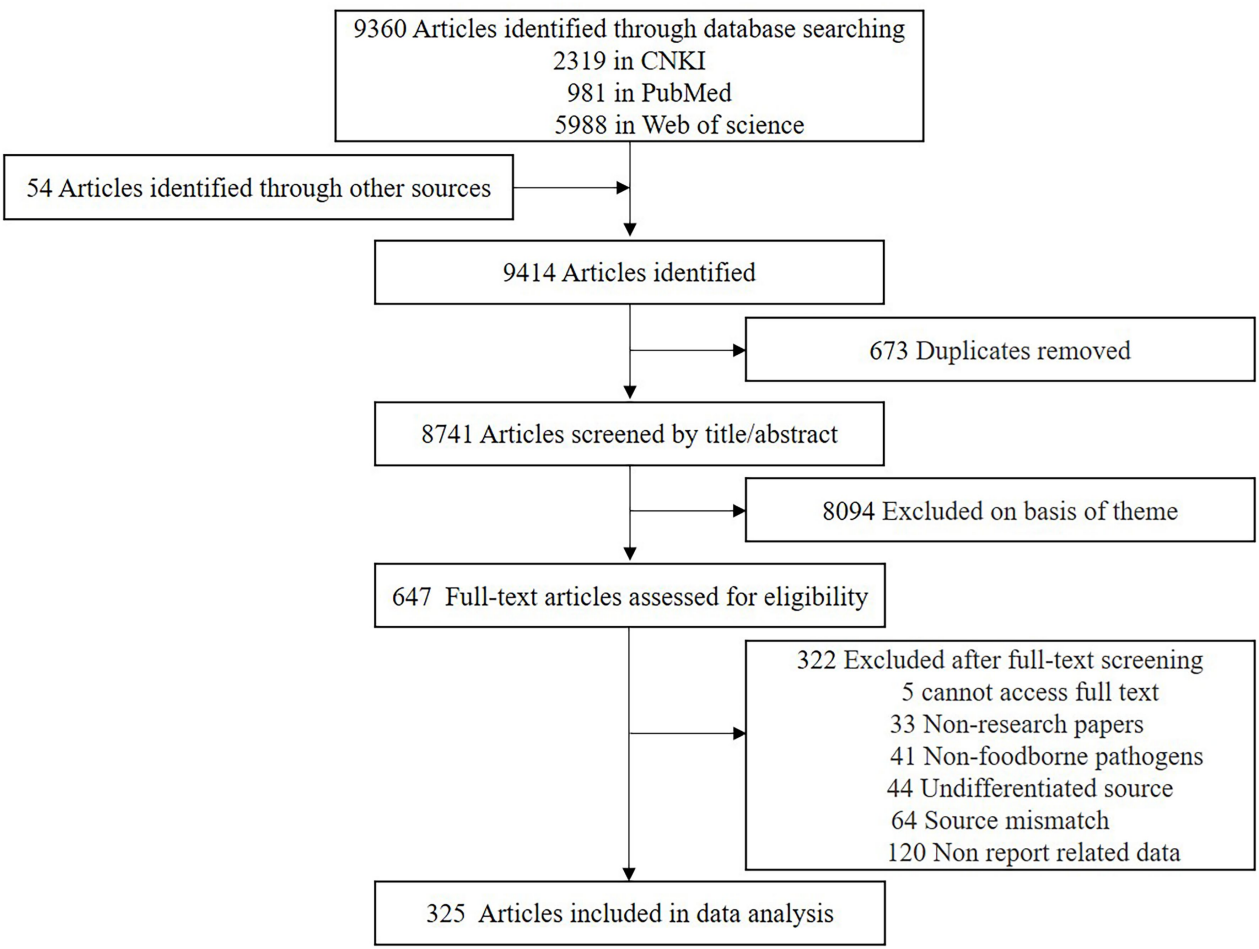
Study selection.

We identified 9,360 articles from electronic databases and 54 articles from other sources. Duplicate articles were removed, and the remaining 8,741 articles were screened for eligibility *via* the title and contents of the abstract (Figure 1). This screening further reduced the number of articles to 647, and a review of the content excluded another 322 articles. The exclusion criteria included five studies, where the text was not accessible. Another 33 articles were review type articles, which included meta-analyses or letters to the editor and 41 studies reported on non-foodborne pathogens. A further 44 studies where the isolates were not attributed to food or human source, and 64 studies were on pathogenic bacteria which were isolated from water or other environmental sources. Finally, 120 studies did not contain sufficient and extractable data.

In total, 325 articles were included in the meta-analysis, where 104 studies reported on antibiotic resistance in patients with foodborne illness and 244 studies reported on antibiotic resistance or biofilm formation in food. Twelve studies reported consequences for both food and patients (Supplementary Table 1), and 11 studies were double-counted because they reported on the result of antibiotic resistance in multiple countries or multiple foodborne pathogens. No unpublished literature met the inclusion criteria.

**Figure 2:**
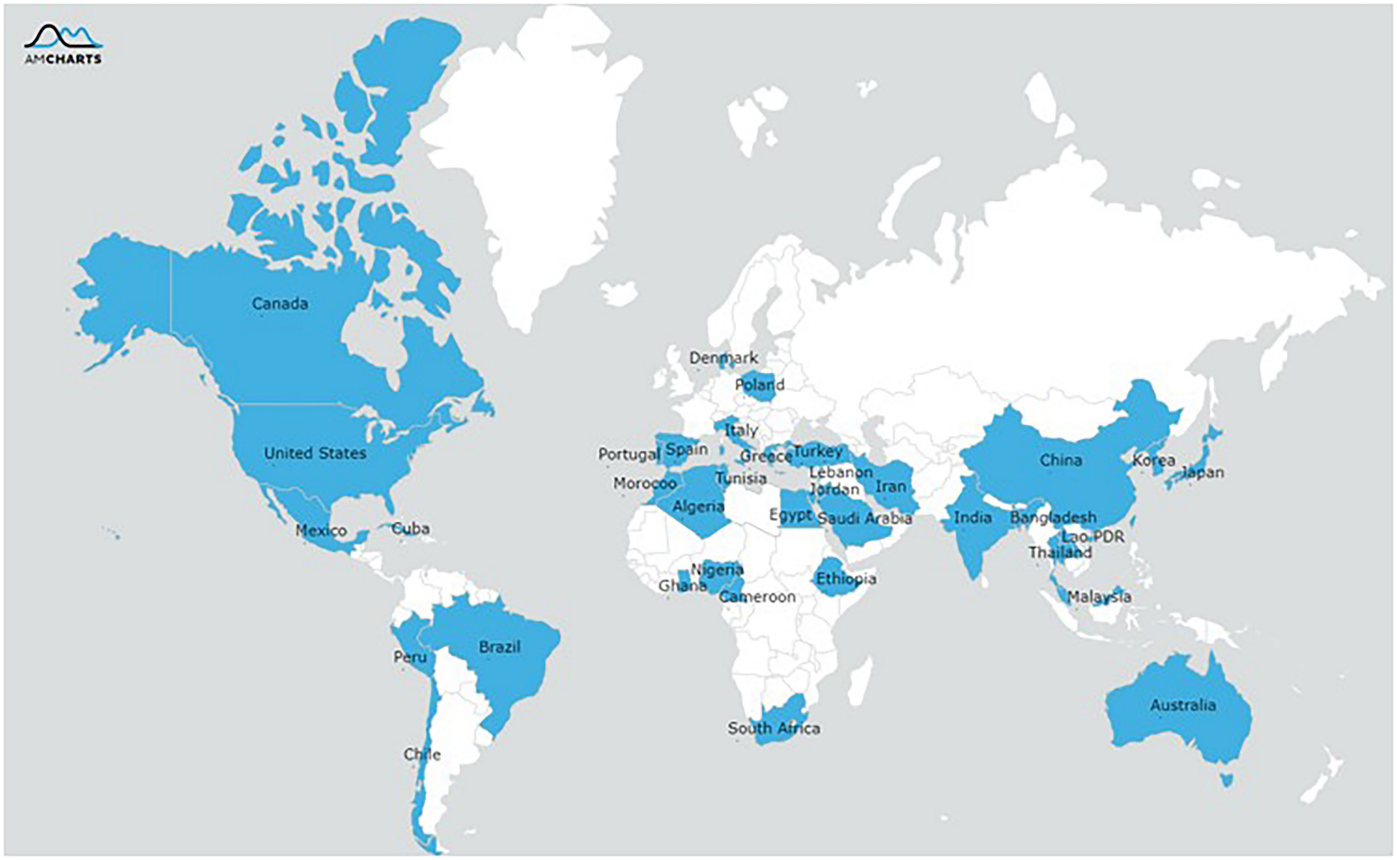
Geographical distribution of reported resistance of the pathogens isolated from food or humans.

The final selection of studies was conducted in 36 different countries and from six continents (Figure 2) with the Asian (*n* = 287[82%]) and Africa regions (*n* = 24[11%]) being predominant. The antibiotic resistance or biofilm formation of following foodborne pathogens were evaluated: *Bacillus cereus* (*n* = 9[3%]), *Escherichia coli* (*n* = 37[11%]), *Listeria monocytogenes* (*n* = 19[5%]), *Vibrio parahaemolyticus* (*n* = 129[37%]), *Salmonella* (*n* = 85[24%]), and *Staphylococcus aureus* (*n* = 58[17%]). All the included studies used a cross-sectional design, and these studies were published in journal articles except for one which was a dissertation.

The 244 food studies included 34,746 isolates of pathogenic bacteria and these strains were isolated from a number of food groups, mainly raw meat (raw beef, mutton, chicken, duck, and pork), raw milk, aquatic products (fish, shrimp, crab, and other seafood), vegetables, and ready-to-eat foods. Only 11 studies reported on the formation of foodborne pathogenic bacteria biofilms, and the biofilm formation of these 752 isolates were measured by quantitative microtiter assay. The 104 human population studies were concentrated foodborne diarrhea patients (*n* = 93[89%]), food poisoning patients (*n* = 10[10%]), and food practitioners (*n* = 1[1%]). Further detailed of these studies study is shown in the Table 1.

**Table 1:** Included studies characteristics.

Study characteristics	Human studies (*n* = 104)	Food studies (*n* = 244)
**Location**		
Africa	2 (2%)	22 (9%)
Asia	99 (95%)	188 (77%)
Europea	1 (1%)	6 (2%)
North America	—	10 (4%)
Oceania	—	1 (1%)
South America	2 (2%)	6 (2%)
**Period**		
2000–2010	21 (20%)	51 (21%)
2011–2015	48 (46%)	108 (44%)
2016–2020	35 (34%)	74 (30%)
**Method of antimicrobial susceptibility testing**		
Disk diffusion	46 (44%)	156 (64%)
MIC	44 (42%)	61 (25%)
Vitek	10 (10%)	10 (4%)
Others	—	4 (2%)
Not reported	4 (4%)	2 (1%)
**Guidelines used to interpret antimicrobial sensitivities**		
CLSI	85 (82%)	199 (82%)
EUCAST	2 (2%)	1 (1%)
NCCLS	6 (6%)	13 (5%)
Others	1 (1%)	6 (2%)
Not reported	10 (10%)	14 (6%)
**Bacteria studied**		
*Bacillus cereus*	1 (1%)	8 (3%)
*Escherichia coli*	11 (10%)	26 (11%)
*Listeria monocytogenes*	1 (1%)	18 (7%)
*Vibrio parahaemolyticus*	50 (48%)	79 (32%)
*Salmonella*	36 (35%)	49 (20%)
*Staphylococcus aureus*	5 (5%)	53 (22%)
**Source of isolate**		
Aquatic products	—	78 (32%)
Meat	—	50 (20%)
Milk and dairy products	—	12 (5%)
RET-food	—	13 (5%)
Others	—	80 (33%)
Foodborne diarrhea patients	93 (89%)	
Food poisoning samples	10 (10%)	
Food handlers	1 (1%)	
**Biofilm forming ability**	—	11 (5%)

To ensure the reliability of the meta-analysis results, we evaluated the quality of the included literature in terms of research purposes, research objects, and research methods, etc. Quality assessment results showed that overall was very complete, there was a low risk of bias (Supplementary Table 4). Both human studies and food studies, which purpose and results of the research are clearly described. The sample characteristics and select of study subjects may cause bias notwithstanding, there are some studies (62% food studies and 30% human studies) with poor description in this part. A full description of the measurement methods and guidelines of antimicrobial sensitivities and biofilm formation was provided in 96% (*n* = 234) of the food studies and 90% (*n* = 94) of the human studies.

### Prevalence of Antibiotic Resistant Foodborne Pathogens

The pooled prevalence of foodborne pathogens resistant to classes of tested antibiotics in food and human studies are shown in Table 2. The mean prevalence of antimicrobial resistant foodborne pathogens isolated in foods was ≥11% and the majority of these foodborne pathogens were highly resistant to β-lactam antibiotics. The combined or pooled prevalence of *B. cereus* resistant to β-lactams antibiotics from all the food studies was 94% (95% CI, 91%–98%), although the presence of *B. cereus* in food was only reported in six studies. The prevalence of *Escherichia coli* which was resistant to all classes of antibiotics tested ranged from 56% (95% CI, 45%–67%) to 25% (95% CI, 15%–34%). Comparatively *Salmonella* was more resistant to chloramphenicol (32, 95% CI, 21%–43%) and *V. parahaemolyticus* was mildly resistant to sulfonamides (14, 95% CI, 10%–18%) and tetracyclines (14, 95% CI, 11%–17%). The Gram-positive pathogen, *L. monocytogenes*, was also mildly resistant to fluoroquinolones (11%; 95% CI, 6%–16%) and sulfonamides (11%; 95% CI, 3%–19%). Lastly *S. aureus* is susceptible to treatment with chloramphenicol (17 9% CI, 12%–19%) when compared to other antibiotics.

**Table 2:** Pooled prevalence of antibiotic resistance from meta-analysis of food studies and human studies, by antibiotic category.

	Food studies		Human studies	
	Articles (*n*)	Prevalence % (95% CI)	Articles (*n*)	Prevalence % (95% CI)
**Gram-negative bacterium**				
***Escherichia coli***				
Aminoglycosides	11	32 (19–46)	8	25 (19–31)
β-Lactams	19	56 (45–67)	10	61 (51–70)
Chloramphenicol	8	25 (15–34)	6	19 (8–30)
Fluoroquinolones	12	37 (22–52)	8	38 (18–58)
Sulfonamides	19	48 (30–65)	9	39 (26–52)
Tetracyclines	21	54 (41–57)	9	49 (43–55)
***Salmonella***				
Aminoglycosides	31	39 (31–47)	17	44 (27–61)
β-Lactams	42	47 (38–55)	33	56 (47–66)
Chloramphenicol	18	32 (21–43)	18	33 (24–42)
Fluoroquinolones	42	44 (30–59)	24	50 (40–60)
Sulfonamides	34	42 (29–54)	26	43 (32–54)
Tetracyclines	30	56 (47–64)	27	45 (35–54)
***Vibrio parahaemolyticus***				
Aminoglycosides	39	45 (36–53)	14	22 (11–33)
β-Lactams	77	77 (71–83)	37	76 (69–82)
Fluoroquinolones	7	13 (7–19)	8	19 (−7–44)
Sulfonamides	36	14 (10–18)	11	25 (−1–52)
Tetracyclines	22	14 (11–17)	6	2 (0–4)
**Gram-positive bacterium**				
***Bacillus cereus***				
β-Lactams	6	94 (91–98)	1	81 (75–86)
Sulfonamides	3	32 (6–58)	1	66 (60–73)
***Listeria monocytogenes***				
Aminoglycosides	6	21 (8–35)	—	—
β-Lactams	13	45 (27–63)	1	54 (38–70)
Chloramphenicol	8	30 (11–49)	-	-
Fluoroquinolones	9	11 (6–16)	1	62 (47–78)
Sulfonamides	6	11 (3–19)	—	—
Tetracyclines	13	22 (15–30)	1	30 (15–44)
***Staphylococcus aureus***				
Aminoglycosides	33	30 (24–36)	2	30 (−12–73)
β-Lactams	45	78 (73–82)	3	68 (32–102)
Chloramphenicol	17	16 (12–19)	—	—
Fluoroquinolones	27	23 (19–28)	1	36 (8–65)
Sulfonamides	19	31 (19–43)	2	35 (5–66)
Tetracyclines	43	41 (33–48)	4	28 (4–52)

As shown in Table 2, the mean prevalence of antibiotic resistant foodborne pathogens isolated from human samples was ≥19% and the outlier was the mean prevalence of tetracycline resistant *V. parahaemolyticus* (2%; 95% CI, 0%–4%). In tandem to pathogens isolated from food samples, the mean prevalence of the pathogen group to β-lactams antibiotics resistance was high, with *B. cereus* at 81% (95% CI, 75%–86%), *V. parahaemolyticus* at 76% (95% CI, 69%–82%) and *L. monocytogenes* at 54% (95% CI, 38%–70%). The pattern of resistance to the classes of antibiotics tested for Gram negative and Gram-negative pathogens is similar to those isolated from the food samples with the exception of *L. monocytogenes*. The Gram-positive bacterium, *L. monocytogenes*, appears to be highly resistant to fluoroquinolones but this information is only based on 1 study.

#### Subgroup Analysis by Food Types

For the food studies, studies with clear classification of food samples were included in our subgroup analysis, mainly in the following categories: aquatic products, meat, milk, and dairy products, RTE-food. Table 3 shows the prevalence of foodborne pathogen food isolates resistant to antibiotics in different food types. The prevalence of multi-drug resistant (MDR) pathogen was ≥36% for all food types, with the highest rates in meat (52%; 95% CI, 40–60). Also in all food types, pathogens resistant to β-lactams were most common (≥57%). In aquatic products, the pooled prevalence of isolates resistant to fluoroquinolones and sulfonamides were both around 13%, but resistance to β-lactams was over six times higher.

**Table 3:** Subgroup analysis of antibiotic resistance by food types.

	Aquatic products		Meat		Milk and dairy products		RTE-food	
	Articles (*n*)	Prevalence % (95% CI)	Articles (*n*)	Prevalence % (95% CI)	Articles (*n*)	Prevalence % (95% CI)	Articles (*n*)	Prevalence % (95% CI)
MDR	9	42 (26–58)	16	52 (40–63)	4	43 (1–84)	8	36 (21–51)
Aminoglycosides	38	43 (34–51)	36	29 (31–47)	7	33 (14–51)	6	35 (24–46)
β-Lactams	65	73 (66–81)	42	62 (52–70)	9	61 (45–77)	10	57 (34–83)
Chloramphenicol	—	—	19	36 (21–51)	4	21 (17–25)	4	25 (16–34)
Fluoroquinolones	11	13 (8–19)	38	39 (25–53)	5	30 (14–46)	9	25 (16–34)
Sulfonamides	34	14 (11–17)	32	47 (31–63)	7	62 (46–78)	7	43 (10–76)
Tetracyclines	21	22 (9–35)	36	62 (54–70)	9	28 (2–55)	8	43 (24–63)

#### Subgroup Analysis by Study Population

Foodborne pathogens isolated from three groups in human studies also had the highest resistance to β-lactams antibiotics (Table 4). The mean prevalence of antibiotic resistant pathogens among foodborne diarrhea patients were ≥32%, with mean prevalence of resistant isolates to sulfonamides of 39% (95% CI, 27%–52%) and tetracyclines at 41% (95% CI, 33%–48%). In comparison to the other groups, the antibiotic resistance rate of food poisoning sample isolates were lower, except for resistance to β-Lactams which was similar. Only one study (Xu et al., 2019) delineated the antibiotic resistance *Salmonella* isolates, the predominant resistance was to β-lactams (65%), followed by that of sulfonamides (47%), tetracyclines (46%).

**Table 4:** Subgroup analysis of antibiotic resistance by study population.

	Diarrhea patients		Food poisoning samples		Food handlers	
	Articles (*n*)	Prevalence % (95% CI)	Articles (*n*)	Prevalence % (95% CI)	Articles (*n*)	Prevalence % (95% CI)
Aminoglycosides	34	32 (23–42)	4	11 (0–21)	1	23 (16–29)
β-Lactams	78	65 (60–71)	6	78 (62–95)	1	65 (57–72)
Chloramphenicol	23	29 (21–37)	—	—	1	37 (30–45)
Fluoroquinolones	41	42 (30–53)	2	22 (6–39)	—	—
Sulfonamides	46	39 (27–52)	2	14 (2–26)	1	47 (37–52)
Tetracyclines	42	41 (33–48)	5	21 (3–39)	1	46 (37–52)

#### Subgroup Analysis by Region, Time Period, and Susceptibility Test

Table 5 shows the regional distribution of reported resistance profile of the pathogens isolated from food or humans and where the majority of the studies were concentrated in Asia (>75%). In South America, the pooled prevalence of antibiotic resistant pathogens was as high as 97% (food) and 90% (humans). In contrast, the prevalence of antibiotic resistant pathogens isolated from human samples was only 48% (95% CI, 40–55) in Europe. Overall, there was a decrease in the prevalence pathogens resistance of food isolate decreased from75% (95% CI, 65–85) before 2010 to 72% (95% CI, 66–79) during in 2011–2015, then increased to 80% (95% CI, 77–84) between in 2016–2020. The pathogens resistance of human isolate had changed with the time of collection, and which had the same resistance trend with food isolate. In general, we found that a trend of antimicrobial resistance of foodborne pathogens firstly decreasing and then increasing during 2000–2020. Subgroup analysis by susceptibility test group showed higher rates of antibiotic resistance tested with Vitek (automatic drug sensitivity analyzer) compared to used disk diffusion and minimum inhibitory concentration (MIC).

**Table 5:** Subgroup analysis of antibiotic resistance by region, time period and susceptibility test.

	Food studies		Human studies	
	Articles ***(n)***	Prevalence % (95% CI)	Articles ***(n)***	Prevalence % (95% CI)
**Region**				
Africa	19	80 (75–86)	2	92 (82–102)
Asia	149	77 (73–81)	93	82 (80–84)
Europe	6	78 (68–89)	1	48 (40–55)
North America	10	76 (66–87)	—	—
Oceania	1	85 (74–95)	—	—
South America	6	97 (95–99)	2	90 (80–100)
**Period**				
Before 2010	40	75 (65–85)	19	80 (75–84)
2011–2015	75	72 (66–79)	38	79 (75–83)
2016–2020	51	80 (77–84)	30	82 (78–86)
**Susceptibility test**				
Disk diffusion	36	82 (79–85)	112	78 (75–81)
MIC	39	77 (72–82)	47	69 (59–79)
Vitek	9	88 (83–93)	9	90 (85–95)

### Prevalence of Biofilm Formation in Foodborne Pathogens

**Figure 3:**
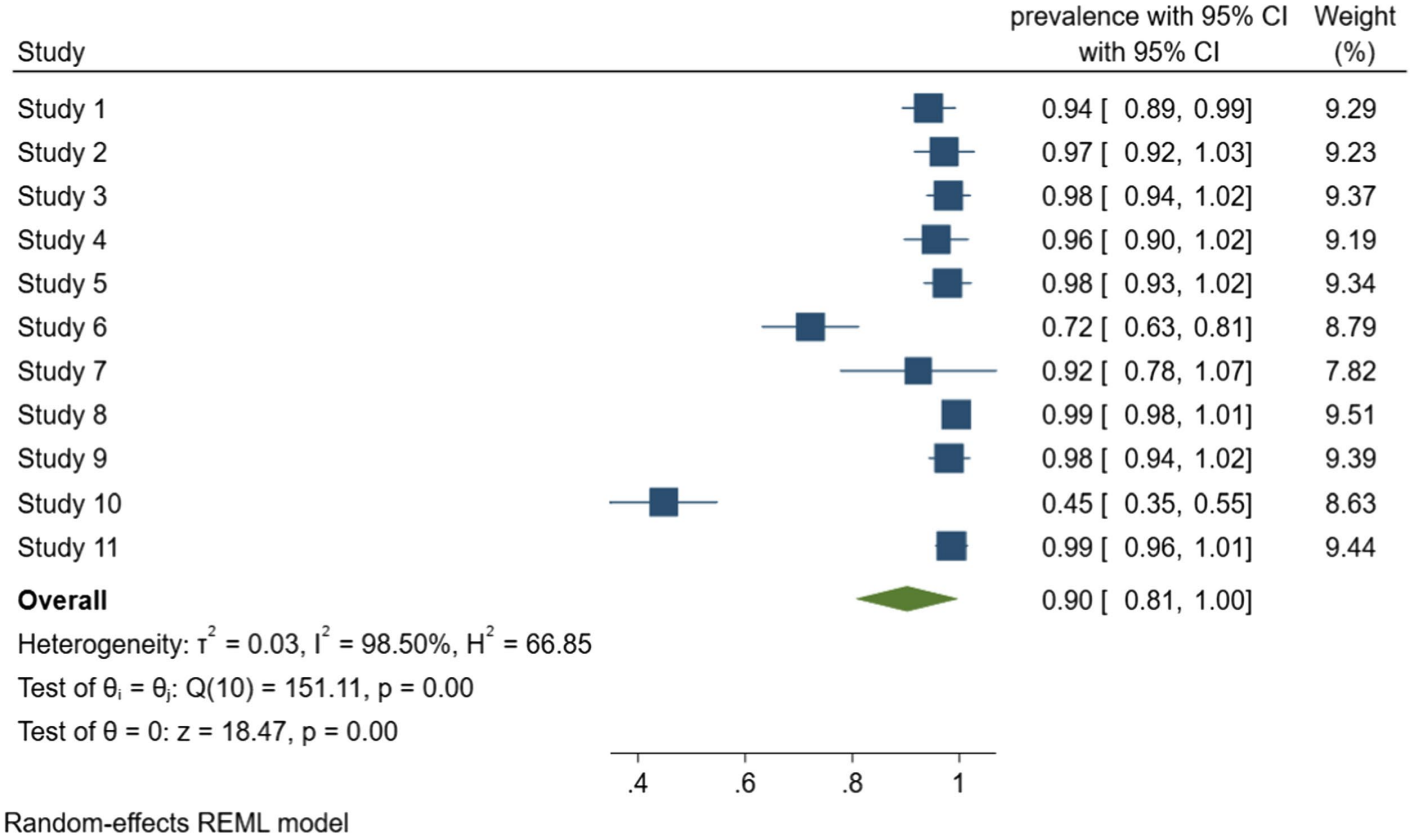
Forest plot of the meta-analysis of biofilm formation rate in foodborne pathogen retrieved from food samples. Study 1: Lapierre et al. (2020). Study 2: Lopez-Leon et al. (2016). Study 3: Rodríguez-Lázaro et al. (2018). Study 4: Beshiru and Igbinosa (2018). Study 5: Beshiru et al. (2018). Study 6: Chen and Xie (2019). Study 7: Maia et al. (2020). Study 8: Ou et al. (2020). Study 9: Puah et al. (2018). Study 10: Wang et al. (2020). Study 11: Kim et al. (2018).

According to meta-analysis of biofilm formation rate in foodborne pathogen retrieved from food samples that shown in Figure 3, the combined rate of biofilm formation was 90% (95% CI, 81–100). Considerable heterogeneity was detected between the studies, with I^2^ = 98.5%, Q (10) = 151.11, *p* < 0.001, which most likely due to the inclusion of multiple species of bacteria. Among them, *Staphylococcus aureus* was the most prone to produce biofilms, all the *Staphylococcus aureus* isolates from food in the four studies can form biofilm. It is worth mentioning that Ou et al. (2020) investigated the antimicrobial resistance and biofilm formation of *staphylococcus aureus* in food, and reported 64.8% of 165 antibiotic resistant strains had strong biofilm formation ability, study describes the significant correlation between antibiotic resistance and biofilm formation.

### Publication Bias

The Begg’s test for funnel plot of the resistance rate of pathogenic bacteria in food and human studies shows that both were exist visual asymmetry evidence, therefore a trim and fill procedure was executed. Funnel plot were produced for of biofilm formation rate of foodborne pathogen retrieved from food samples too, which no evidence of publication bias. Funnel plot shown in Appendix, Supplementary Material.

## Discussion

### Prevalence of Antibiotic Resistance of Foodborne Pathogens

This meta-analysis showed that foodborne pathogens present high levels of antibiotic resistance, both in food samples and clinical specimens. Worldwide, rates of resistance to β-lactams were the highest, irrespective of types of foodborne pathogens. Analogously, a meta-analysis by Jia et al. (Kai et al., 2020) retrieved resistance data of *Staphylococcus aureus* isolates from retail foods, between 2007 and 2017 from70, The main finding was that resistance by *S. aureus* found in retail foods to seven types of antibiotics ranged from 8% to 87% with the most serious resistance being to beta-lactam antibiotics.

Overall, human isolates of *Salmonella* and *Listeria monocytogenes* are significantly more resistant to various antibiotics than food isolates. According to the Microbiological Risk Assessment 2020 report, Salmonellosis is one of the most common zoonotic diseases; the number of listeriosis had increased compared to before [EFSA (European Food Safety Authority), 2020], which might be related to the increased antibiotic resistance of pathogenic bacteria. Our research found a resistance rate increase of 41%–52% in the proportion of isolates that were *Listeria monocytogenes* resistant to fluoroquinolones in human studies compared with food studies. By contrast, for the other pathogens, there is no absolute difference in antibiotic resistance between humans and food isolates. For example, for *Escherichia coli*, food isolates are more resistant to β-lactams and fluoroquinolones than human isolates, which had an opposite of resistance to the other four types of antibiotics.

In the subgroup analysis by food types, foodborne pathogenic bacteria in meat were significantly more resistant to antibiotics than other food isolates, which might be related to the amount of antibiotics used. In farming, antibiotic use in food animal production accounts for two-thirds of the overall antibiotic usage (Done et al., 2015). Meat contamination is mainly caused by *Escherichia coli*, *Salmonella*, and *Staphylococcus aureus* (Adesokan et al., 2020), our reported pooled multi-drug resistance rate of foodborne pathogens in meat was 52%, which was similar to the reported antimicrobial resistance in zoonotic and indicator bacteria from food by EFSA (European Food Safety Authority) and ECDC (European Centre for Disease Prevention and Control) (2021). Meanwhile, we find that the resistance of pathogenic bacteria in meat to antibiotics were, in increasing order of resistance, aminoglycosides, chloramphenicol, fluoroquinolones, sulfonamides, β-lactams, and tetracyclines. To 62% resistance to tetracyclines and β-lactams was observed.

In the subgroup analysis by study population, we found that pathogenic bacteria isolated from food handlers and foodborne diarrhea patients have similar drug resistance. The antibiotic resistance of pathogenic bacteria in food handlers was significantly stronger than that in food poisoning samples, except for resistance to β-lactams. Because of direct or indirect contact with food, food handlers are likely to transmit drug-resistant foodborne pathogens to consumers through food. Franck et al. (2014) found that in the 191 foodborne disease outbreaks in Denmark between 2005 and 2011, food handlers played an indispensable role. There have also been 43 cases of infections caused by *Salmonella* carried by food handlers in the United States (Kimura et al., 2005).

In general, the antibiotic resistance rates of foodborne pathogens from six continents were at high levels, regardless of the source or food samples isolated from clinical specimens. A notable finding was the difference in the summary analysis value of antibiotic resistance rate of foodborne pathogens between regions, which was significantly higher in Africa and South America compared to others. While most countries in South America and Africa are low-income and middle-income countries, antibiotic consumption was greater than in high-income countries (Klein et al., 2020). Heavy use of antibiotics will make the enhanced bacterial resistance (Oliveira et al., 2021), in the European Union and the United States, according to reports, agriculture accounts for more than 75% of the annual antibiotic use; between 2011 and 2014, the use of antibiotics in 24 European Union countries fell by 12% [OECD (Organization for Economic Cooperation and Development), 2016]. By time period, in our subgroup analysis, antibiotic resistance of foodborne pathogens also declined during 2011–2015. In general, we detected that the resistance rate foodborne pathogens in food samples and human specimens had increased.

In the studies included, most of the studies applied the antibiotic susceptibility testing methods (disk diffusion and MIC) recommended by the Clinical and Laboratory Standards Institute (CLSI Clinical and Laboratory Standards Institute, 2019). Our meta-analysis shows that foodborne pathogens measured with Vitek had a higher resistance rate, which might be related to the higher sensitivity of the automatic drug sensitivity analyzer (Nakasone et al., 2007). In fact, sensitivity testing methods will not have a substantially impact on bacterial resistance. The resistance of foodborne pathogens bacteria depends on several factors, such as type of bacteria, the type of food and disease, the source of the sample, and other related factors.

### Prevalence of Biofilm Formation of Foodborne Pathogens

We described the biofilm formation rate of foodborne pathogen, and most foodborne pathogens have the ability to form biofilms in the food studies included. Due to lack of relevant research, we did not find evidence of biofilm formation of foodborne pathogens isolated from humans.

The mechanisms by which biofilms promote resistance of bacteria to antimicrobials is very complicated. A series of molecular mechanisms occur can conduce to the stability of the biofilm microbial community (Hall and Mah, 2017). These occur in the main in the following three ways. First, interaction between biofilm matrix components and antibiotics; there are factors in the biofilm that can reduce the penetration rate of antibiotics (Boudjemaa et al., 2016; Singh et al., 2016). Second, bacteria in biofilms evade external stimuli by reducing their growth rate (Walters et al., 2003; Borriello et al., 2004). Third, the role of specific genetic determinants of antibiotic resistance in biofilms, such as efflux pumps (Van Acker and Coenye, 2016), quorum sensing (Chua et al., 2016), colony variants (Proctor et al., 2006), et al. The results of our meta-analysis show that 90% of the 752 foodborne pathogens can form biofilms, this provides the possibility of enhancing their resistance. However, due to the limitations of existing research, we cannot provide evidence of a direct linear relationship between biofilm formation ability and antibiotic resistance.

### Strengths and Limitations

To our knowledge, this study is the first comprehensive meta-analysis of global prevalence of antibiotic resistance and biofilm formation in foodborne pathogens. We were able to include 11 studies (Supplementary Table 3) with assess the biofilm formation of foodborne pathogens in food, and the relationship between the ability of biofilm formation and antibiotic resistance was discussed. One of our main advantages is that strict compliance with PRISMA guidelines (Moher, 2010), we have adopted strict standards and limited to research that analyzed the resistance of foodborne pathogen in food and humans, the reservoirs of pathogenic bacteria, and the main vehicle of antibiotic resistance transmitted through the food chain.

Our meta-analysis has some limitations. First, there was significant heterogeneity among the included studies in the analysis. As we undertook a bundled meta-analysis (Tang et al., 2017), it is reasonable that no relevant reduction of heterogeneity was detected when we conducted subgroup analysis from sample type, region, date of sample collection and susceptibility test. Furthermore, another potential source of heterogeneity may be related to antibiotic consumption patterns in various regions. Second, because of we limited by study quality, there was potential data reporting bias in this meta-analysis. Studies which met the inclusion criteria and which reported the prevalence of antibiotic resistance of foodborne pathogens isolated from food handlers was scarce and is, as a consequence, poorly represented.

## Conclusion

In conclusions, this study provides a comprehensive overview of global antibiotic resistance of foodborne pathogen in food and humans, which shows worrying levels of resistance in some parts of the world, where one possible explanation is large and irregular use of antibiotics. Following preliminary pooling of data relating to biofilm formation of pathogenic bacteria, our analysis shows that foodborne pathogens have a high tendency to form biofilms. It is less clear that a direct linear relationship exists between the ability to form biofilms and antibiotic resistance. It is recommended that future research should thoroughly explore the relationship between the ability of biofilm formation and the antibiotic resistance of foodborne pathogens and provide a new theoretical basis for the discovery of the mechanism of foodborne pathogens’ antibiotic resistance. Therefore, frequent monitoring of types, antibiotic resistance and biofilm characteristics of foodborne pathogens in clinical and environmental is urgently needed to raise our awareness of antibiotic resistance and its spread, and prompt the development of effective strategies to improve food safety and prevent foodborne illness infection.

## Data Availability Statement

The original contributions presented in the study are included in the article/Supplementary Material, and further inquiries can be directed to the corresponding authors.

## Author Contributions

QT, QW, and ZZ interpreted the literature sources and drafted the manuscript. QT and QW conceived and planned the data collection and analysis. JL, CT, and ZH involved in data verification. ZZ, PM, and YP critically reviewed and revised the manuscript. ZZ and YZ conceptualized the idea and edited the manuscript. All authors contributed to the article and approved the submitted version.

## Funding

This research was supported by Innovation Program of Shanghai Municipal Education Commission (2017-01-07-00-10-E00056) and Program of Shanghai Academic Research Leader (21XD1401200).

## Conflict of Interest

The authors declare that the research was conducted in the absence of any commercial or financial relationships that could be construed as a potential conflict of interest.

## Publisher’s Note

All claims expressed in this article are solely those of the authors and do not necessarily represent those of their affiliated organizations, or those of the publisher, the editors and the reviewers. Any product that may be evaluated in this article, or claim that may be made by its manufacturer, is not guaranteed or endorsed by the publisher.
