# Supplementary material for: Meta-Analysis for the Global Prevalence of Foodborne Pathogens Exhibiting Antibiotic Resistance and Biofilm Formation
Source: Front Microbiol. 2022 Jun 14;13:906490. doi: 10.3389/fmicb.2022.906490 (PMC9239547; doi:10.3389/fmicb.2022.906490)
Supplement: Supplementary file 1 [file Data_Sheet_1.PDF]

Supplementary material: Table of Contents

Table 1: Example of search strategy 2

Table 2. Characteristics of included antibiotic resistance studies...............3

Table 3. Characteristics of included biofilm studies.................................20

Table 4. Quality Assessment of Included Studies......................................21

Supplementary forest plots...22

Supplementary Funnel plots...75

References for included studies 78

Table 1: Example of search strategy

**PubMed**
((((((Antimicrobial Drug Resistance[Title/Abstract]) OR (Antimicrobial Drug Resistances[Title/Abstract])) OR (Antibiotic Resistance[Title/Abstract])) OR (Antibiotic-resistant[Title/Abstract])) OR (Antimicrobial Resistance[Title/Abstract6])) OR (((biofilm[Title/Abstract]) OR (Bacterial Biofilm[Title/Abstract])) OR (Biofilm formation[Title/Abstract]))) AND ((((food-borne pathogenic bacteria[Title/Abstract]) OR (foodborne pathogen[Title/Abstract])) OR (foodborne pathogenic bacteria[Title/Abstract])) OR (("Foodborne Diseases"[Mesh]) OR (((((((((((((((((Disease, Foodborne[Title/Abstract]) OR (Foodborne Illnesses[Title/Abstract])) OR (Foodborne Disease[Title/Abstract])) OR (Food-borne Illnesses[Title/Abstract])) OR (Food borne Illnesses[Title/Abstract])) OR (Food-borne Disease[Title/Abstract])) OR (Food borne Disease[Title/Abstract])) OR (Food-borne Illness[Title/Abstract])) OR (Food borne Illness[Title/Abstract])) OR (Illness, Food-borne[Title/Abstract])) OR (Foodborne Illness[Title/Abstract])) OR (Illness, Foodborne[Title/Abstract])) OR (Illnesses, Foodborne[Title/Abstract])) OR (Food-borne Diseases[Title/Abstract])) OR (Disease, Food-borne[Title/Abstract])) OR (Food borne Diseases[Title/Abstract])) OR (Food Poisoning[Title/Abstract])) OR (Poisoning, Food[Title/Abstract])) OR (Food Poisonings[Title/Abstract])))) Filters: from 2010 - 2020
**Web of science**
“TS=(biofilm OR Bacterial Biofilm OR Biofilm formation)” OR “TS=(Antimicrobial Drug Resistance OR Antimicrobial Drug Resistances OR Antibiotic Resistance OR Antibiotic-resistant OR Antimicrobial Resistance)” AND “TS=(foodborne pathogen OR foodborne pathogenic bacteria OR foodborne illnesses OR foodborne disease OR food borne diseases OR food poisoning)”

Table 2. Characteristics of included antibiotic resistance studies.

Study	Period	Location	Pathogenic Bacteria	Source	Strains (n)	Susceptibility Test	Guidelines	Resistance strains (n)
Abass A (2020)[1]	—	Ghana	*Escherichia coli*	meat	38	Disk diffusion test	CLSI	36
Abdalrahman LS (2015)[2]	2010	USA	*staphylococcus aureus*	meat	283	Disk diffusion test	CLSI	261
Adesokan HK (2020)[3]	2017	Nigeria	*staphylococcus aureus*	meat	76	Disk diffusion test	CLSI	76
			salmonella		36			15
			Listeria monocytogenes		40			36
Adzitey F (2020)[4]	—	Ghana	*Escherichia coli*	meat	60	Disk diffusion test	CLSI	60
Adzitey F (2020)[5]	—	Ghana	*salmonella*	meat	44	Disk diffusion test	CLSI	8
Ali DA (2020)[6]	2013-2014	Ethiopia	*salmonella*	broiler	50	Kirby-Bauer	CLSI	48
Amajoud N (2017)[7]	2010-2012	Morocco	*salmonella*	food	48	Disk diffusion test	CLSI	19
An XH (2010)[8]	2008	China	*Vibrio parahemolyticus*	aquatic products	105	Kirby-Bauer	NCCLS	75
Aydin A (2011)[9]	2007-2008	Turkey	*staphylococcus aureus*	food	154	Agar dilution	CLSI	139
Baghbaderani ZT (2020)[10]	2018	Iran	*staphylococcus aureus*	meat	48	Disk diffusion test	CLSI	38
Bai Y (2017)[11]	2015	China	*Vibrio parahemolyticus*	aquatic products	1046	Microdilution broth method	CLSI	965
Bai Y (2018)[12]	2018	China	*Vibrio parahemolyticus*	food	1137	Microdilution broth method	CLSI	883
Ballga PV (2019)[13]	—	India	*Vibrio parahemolyticus*	aquatic products	46	Kirby-Bauer	CLSI	42
Beshiru A (2019)[14]	2016-2017	Nigeria	*salmonella*	RTE-shimps	45	Kirby-Bauer	CLSI	39
Bi XY (2015)[15]	2013-2014	China	*Vibrio parahemolyticus*	aquatic products	101	Disk diffusion test	NCCLS	77
Bissong FEA (2020)[16]	2018	Cameroon	*staphylococcus aureus*	beef milk	50	Disk diffusion test	CLSI	41
Bokharaei NM (2020)[17]	—	Iran	*staphylococcus aureus*	Hamburger	39	Disk diffusion test	CLSI	28
Cai XF (2011)[18]	2008-2010	China	*staphylococcus aureus*	food	50	Analyzer		50

Study	Period	Location	Pathogenic Bacteria	Source	Strains (n)	Susceptibility Test	Guidelines	Resistance strains (n)
Cai YQ (2018)[19]	2014-2017	China	*Vibrio parahemolyticus*	aquatic products	37	Kirby-Bauer	CLSI	27
Canizalez-Roman A (2013)[20]	2008-2009	Mexico	*Escherichia coli*	food	56	Agar dilution	CLSI	37
Capalonga R (2014)[21]	2007-2012	Brazil	*salmonella*	foodborne diseases	163	Disk diffusion test	CLSI	138
Castro A (2017)[22]	2009	Portugal	*staphylococcus aureus*	food	73			72
Chen Y (2015)[23]	2009-2013	China	*Vibrio parahemolyticus*	diarrheal disease	501	Agar dilution	CLSI	436
Chen GL (2019)*	2013-2015	China	*staphylococcus aureus*	food	53	Disk diffusion test	CLSI	43
Chen GL (2016)[24]	2013-2014	China	*staphylococcus aureus*	food	38	Kirby-Bauer	WS/T	34
Chen JH (2014)[25]	2006-2011	China	*salmonella*	foodborne diseases	449	Kirby-Bauer	CLSI	448
Chen LP (2016)*	2013-2014	China	*Vibrio parahemolyticus*	diarrheal disease	131	Kirby-Bauer	CLSI	121
Chen Q (2019)[26]	2015-2016	China	*staphylococcus aureus*	food poisoning	37	Guidebook		11
Chen RL (2010)*	—	China	*Vibrio parahemolyticus*	food poisoning	38	Microdilution broth method	CLSI	33
Chen XH (2010)*	2009	China	*Vibrio parahemolyticus*	diarrheal disease	228	Kirby-Bauer	CLSI	226
Chen XN (2020)[27]	2016-2017	China	*Vibrio parahemolyticus*	diarrheal disease	182	Vetek 2 AST	CLSI	144
Chen YH (2012)[28]	2011	China	*Vibrio parahemolyticus*	aquatic products	60	Disk diffusion test	CLSI	40
Chen YZ (2016)[29]	2010-2014	China	*Vibrio parahemolyticus*	diarrheal disease	97	Microdilution broth method	CLSI	
				aquatic products	210			4
Chen YZ (2012)[30]	2003-2010	China	*salmonella*	food	111	Microdilution broth method	CLSI	110
Chen ZQ (2020)[31]	2017-2019	China	*salmonella*	meta	151	microdilution broth method	CLSI	151
Cho YS (2019)[32]	—	Korea	*staphylococcus aureus*	bakery	42	Vitek 2	CLSI	37
Chon JW (2012)[33]	2007-2008	Korea	*Bacillus cereus*	RET-food	35	Kirby-Bauer	CLSI	35
Cui HX (2011)[34]	2010	China	*staphylococcus aureus*	food	46	Microdilution broth method	CLSI	46

Study	Period	Location	Pathogenic Bacteria	Source	Strains (n)	Susceptibility Test	Guidelines	Resistance strains (n)
			salmonella		40			40
Cui HX (2014)[35]	2012	China	*Listeria monocytogenes*	food	34	Minimal broth method	CLSI	34
Cui X (2019)[36]	2015-2018	China	*staphylococcus aureus*	food	103	Microdilution broth method	CLSI	102
Cunha-Neto AD (2018)[37]	2014-2015	Brazil	*salmonella*	chicken carcasses	31	Disk diffusion test	CLSI	31
Dallal MMS (2020)[38]	2015-2016	Iran	*salmonella*	foodborne diseases	74	Microdilution broth method	CLSI	73
Dehkordi FS (2018)[39]	2016-2017	Iran	*staphylococcus aureus*	food	47	Disk diffusion test	CLSI	47
Ed-Dra A (2018)[40]	2014-2015	Morocco	*staphylococcus aureus*	sausages	63	Disk diffusion test	CLSI	61
Eguale T (2018)[41]	2013-2014	Ethiopia	*salmonella*	foodborne diarrhea patients	68	Kirby-Bauer	CLSI	59
Elexson N (2014)[42]	—	Malaysia	*Vibrio parahemolyticus*	aquatic products	36	Disc diffusion	CLSI	36
Elhadi N (2014)[43]	2012-2013	Saudi Arabia	*salmonella*	fish	140	Disk diffusion test	CLSI	140
Fadlallah SM (2017)[44]	2011-2014	Lebanon	*salmonella*	food	49	Kirby-Bauer	CLSI	10
Fallah AA (2012)[45]	2010-2011	Iran	*Listeria monocytogenes*	meat	52	Disc diffusion test	CLSI	31
Fang WJ (2016)[46]	2014	China	*Vibrio parahemolyticus*	diarrheal disease	46	Kirby-Bauer		46
Farhooumand P (2020)[47]	2018	Iran	*Listeria monocytogenes*<br>*Escherichia coli*	meat	45<br>71	Disc diffusion test	CLSI	45<br>71
Feng HR (2013)[48]	2012	China	*Vibrio parahemolyticus*	diarrheal disease	78	Disk diffusion test	CLSI	73
Fri J (2020)[49]	—	South Africa	*staphylococcus aureus*	fish	33	Disk diffusion test	CLSI	32
Fu FB (2018)[50]	2008-2017	China	*Vibrio parahemolyticus*	aquatic products	320	Kirby-Bauer	CLSI	18
Fu HQ (2012)[51]	2010-2011	China	*Vibrio parahemolyticus*	diarrheal disease	299	Kirby-Bauer	CLSI	297

Study	Period	Location	Pathogenic Bacteria	Source	Strains (n)	Susceptibility Test	Guidelines	Resistance strains (n)
Fu YF (2013)[52]	2011	China	*Vibrio parahemolyticus*	diarrheal disease	97	Kirby-Bauer	CLSI	96
Fu YX (2020)[53]	2019	China	*Escherichia coli*	diarrheal disease	32	broth dilution method	CLSI	25
Gao H (2019)[54]	2017	China	*Vibrio parahemolyticus*<br>*salmonella*	diarrheal disease	169<br>139	Microdilution broth method	CLSI	167<br>113
Gao YL (2014)[55]	—	China	*staphylococcus aureus*	milk	82	Microdilution broth method	CLSI	82
Ghoddusi A (2019)[56]	2008-2014	Iran	*salmonella*	clinical samples	109	Agar dilution	CLSI	75
Gousia P (2011)[57]	2004-2007	Greece	*Escherichia coli*	meat	157	Disk diffusion test	CLSI	155
Gu WH (2018)[58]	2014	China	*Vibrio parahemolyticus*	food	71	Microdilution broth method	CLSI	37
Gu XL (2018)*	2017	China	*Vibrio parahemolyticus*	Singmon	65	Vitek 2 compact30	AST-GN14	62
Guan WH (2019)[59]	2017	China	*Vibrio parahemolyticus*	ostracean-shrimp	84	Kirby-Bauer	CLSI	83
Han Y (2016)[60]	2012-2014	China	*Vibrio parahemolyticus*	diarrheal disease	92	Kirby-Bauer	NCCLS	38
Hao RE (2020)*	2016-2019	China	*Vibrio parahemolyticus*	disease	75	broth dilution	CLSI	74
Harb A (2018)[61]	2015-2016	Australia	*salmonella*	chicken carcasses	46	Disk diffusion test	CLSI	39
Hassena AB (2019)[62]	2011-2012	Tunisia	*salmonella*	milk meat	45	Disk diffusion test	CLSI	38
He LH (2012)[63]	2008-2011	China	*salmonella*	meat	69	Kirby-Bauer	CLSI	39
He SN (2015)[64]	2011-2014	China	*Vibrio parahemolyticus*	diarrheal disease	52	Kirby-Bauer	CLSI	46
He Y (2016)[65]	2013-2014	China	*Vibrio parahemolyticus*	shrimp	35	Kirby-Bauer	CLSI	35
He Y (2014)[66]	2012	China	*staphylococcus aureus*	milk	37	Kirby-Bauer	CLSI	37
Hong J (2015)[67]	2009-2011	Korea	*staphylococcus aureus*	leaf vegetable	53	Disc diffusion test	CLSI	53
Hou LJ (2013)[68]	—	China	*Vibrio parahemolyticus*	aquatic products	35	Kirby-Bauer	CLSI	32
Hou Z (2017)[69]	2012-2015	China	*Listeria monocytogenes*	food	50	E-test	CLSI	11
Hu QX (2016)[70]	2014	China	*Vibrio parahemolyticus*	aquatic products	208	Kirby-Bauer	CLSI	197
Hu YJ (2018)[71]	2016	China	*salmonella*	food	755	Microdilution broth method	CLSI	549

Study	Period	Location	Pathogenic Bacteria	Source	Strains (n)	Susceptibility Test	Guidelines	Resistance strains (n)
Hu YJ (2017)[72]	2015	China	*salmonella*	food	1070	Microdilution broth method	CLSI	769
Huang CM (2016)[73]	—	China	*Vibrio parahemolyticus*	diarrheal disease<br>aquatic products	30<br>46	Kirby-Bauer	CLSI	30<br>46
Huang HJ (2020)[74]	2016-2018	China	*salmonella*	foodborne diarrhea patients	170	—	NFDMM	149
Huang JY (2015)[75]	2010-2013	China	*Vibrio parahemolyticus*	diarrheal disease	108	Disk diffusion test	CLSI	98
Huang JY (2010)[76]	2006-2009	China	*Bacillus cereus*	clinical specimens	208	Kirby-Bauer	NCCLS	168
Huang LL (2016)*	—	China	*staphylococcus aureus*	food	38	Kirby-Bauer	—	38
Huang MR (2014)[77]	2012-2013	China	*Vibrio parahemolyticus*	aquatic products	43	Vitek 2	CLSI	40
Huang SH (2017)*	2015	China	*salmonella*	food	47	BD	—	47
Iwabuchi E (2010)[78]	2006-2008	Japan	*salmonella*	chicken carcasses	452	Disk diffusion test	CLSI	443
Jaja IF (2020)[79]	2016-2017	South Africa	*staphylococcus aureus*	meat	142	Kirby-Bauer	CLSI	142
Jamali H (2014)[80]	—	Malaysia	*Listeria monocytogenes*	food	32	Disk diffusion test	CLSI	17
Jamali H (2015)[81]	2006-2013	Malaysia	*staphylococcus aureus*	food	328	disc diffusion test	CLSI	119
Jeamsripong S (2020)[82]	—	Thailand	*Vibrio parahemolyticus*	oyster	361	agar dilution	CLSI	187
Jia HY (2016)[83]	2010-2013	China	*Vibrio parahemolyticus*	aquatic products	221	Microdilution broth method	CLSI	71
Jia HY (2013)[84]	—	henan	*staphylococcus aureus*	RET food	68	Microdilution broth method	CLSI	44
Jiang H (2020)[85]	2017-2019	China	*Vibrio parahemolyticus*	shrimp	90	Disk diffusion test	CLSI	69
Jiang LX (2010)[86]	2008-2009	China	*Vibrio parahemolyticus*	aquatic products	113	ATB Fungs	—	75
Jiang Y (2014)[87]	2009-2010	China	*Vibrio parahemolyticus*	sea cucumbers	87	Disc diffusion method	CLSI	87
Jiang YH (2019)[88]	2009-2016	China	*Vibrio parahemolyticus*	aquatic products	90	Kirby-Bauer	CLSI	86

Study	Period	Location	Pathogenic Bacteria	Source	Strains (n)	Susceptibility Test	Guidelines	Resistance strains (n)
Jiang YH (2018)[89]	2015-2016	China	*Vibrio parahemolyticus*	aquatic products	165	Kirby-Bauer	NCCLS	159
Jiang YH (2015)[90]	2014	China	*Vibrio parahemolyticus*	aquatic products	84	Kirby-Bauer	CLSI	77
Jiang YH (2013)[91]	2012	China	*Vibrio parahemolyticus*	aquatic products	50	Kirby-Bauer	CLSI	49
Jiang YH (2012)[92]	2010	China	*Vibrio parahemolyticus*	aquatic products	98	Kirby-Bauer	NCCLS	91
Jiang Z (2018)[93]	2010-2015	China	*Vibrio parahemolyticus*	aquatic products	52	Minimal broth	CLSI	23
Jin JH (2013)[94]	—	China	*Vibrio parahemolyticus*	aquatic products	116	Kirby-Bauer	CLSI	97
Kang CH (2017)[95]	2015	Korea	*Vibrio parahemolyticus*	oysters	44	Disk diffusion test	NCCLS	38
Kang CH (2018)[96]	2016	Korea	*Vibrio parahemolyticus*	oysters	59	Disk diffusion test	NCCLS	59
Kassem II (2020)[97]	2018	Lebanon	*Escherichia coli*	beef	120	disk diffusion test	CLSI	120
Kevenk TO (2016)[98]	2011-2012	Turkey	*Listeria monocytogenes*	milk dairy	54	Disc diffusion test	CLSI	27
Kizil S (2020)[99]	—	Turkey	*salmonella*	food poisoning	34	Microdilution broth method	CLSI	19
KOO HJ (2012)[100]	2004-2006	Korea	*Escherichia coli*	food	162	Disk diffusion test	CLSI	121
Lampugnani C (2020)[101]	—	Brazil	*staphylococcus aureus*	milk	59	Disk diffusion test	CLSI	59
Lan T (2020)[102]	2017	China	*Escherichia coli*	milk	92	Microdilution broth method	CLSI	40
Lapierre L (2020)[103]	—	Chile	*salmonella*	meat	87	Kirby-Bauer	CLSI	87
Lee LH (2018)[104]	2016	Malaysia	*Vibrio parahemolyticus*	fish	165	Kirby-Bauer	CLSI	145
Lee N (2012)[105]	—	Korea	*Bacillus cereus*	RET-food	39	Disk diffusion test	CLSI	39
Letchumanan V (2015)[106]	2014	Malaysia	*Vibrio parahemolyticus*	shrimps<br>aquatic products	185<br>200	Disk diffusion test	CLSI	151<br>176
Li BB (2019)[107]	2017-2018	China	*staphylococcus aureus*	meats	60	Agar dilution	CLSI	54
Li BB (2016)[108]	2015	China	*salmonella*	food	32		NCCLS	32
Li HJ (2020)[109]	2015-2017	China	*Vibrio parahemolyticus*	diarrheal disease	72	Disk diffusion test	CLSI	64
Li LQ (2020)[110]	2018	China	*Vibrio parahemolyticus*	shellfish	140	Disk diffusion test	CLSI	140

Study	Period	Location	Pathogenic Bacteria	Source	Strains (n)	Susceptibility Test	Guidelines	Resistance strains (n)
Li MH (2019)[111]	2017	China	*staphylococcus aureus*	food	397	Microdilution broth method	CLSI	383
Li MJ (2017)*	2016	China	*Escherichia coli*	foodborne diseases	123	Microdilution broth method	CLSI	103
Li N (2019)[112]	2012	China	*Vibrio parahemolyticus*	diarrheal disease	65	Agar dilution	CLSI	6
				aquatic products	65			58
Li P (2019)[113]	2014-2016	China	*Vibrio parahemolyticus*	aquatic products	65	Disk diffusion test	CLSI	62
Li QC (2019)[114]	—	China	*staphylococcus aureus*	meat	104	Disk diffusion test	CLSI	101
Li SJ (2016)[115]	—	China	*Escherichia coli*	diarrheal disease	37	Agar dilution	CLSI	18
Li SJ (2016)[116]	—	China	*Escherichia coli*	diarrheal disease	41	Agar dilution	CLSI	41
Li X (2020)[117]	2018	China	*salmonella*	diarrhea patients	80	Microdilution broth method	CLSI	71
Li YC (2014)[118]	2011	China	*salmonella*	pork	163	Disk diffusion test	CLSI	134
Li YH (2017)[119]	2009-2014	China	*Escherichia coli*	diarrheal disease	168	Disc diffusion test	CLSI	130
Li YP (2020)[120]	2015-2016	China	*Vibrio parahemolyticus*	food	150	Disk diffusion test	CLSI	133
Li YT (2015)[121]	2014	China	*staphylococcus aureus*	food	59	Microdilution broth method		59
Li Z (2019)[122]	2015-2016	China	*Vibrio parahemolyticus*	diarrheal disease	46	Agar dilution	CLSI	3
Li ZY (2020)*	—	China	*Escherichia coli*	slaughterhouse	158	Kirby-Bauer	CLSI	157
Lin YT (2019)[123]	2015-2017	China	*Vibrio parahemolyticus*	diarrheal disease	30	Kirby-Bauer	CLSI	30
				aquatic products	46			46
Liu J (2018)[124]	2009-2016	China	*salmonella*	raw meat	109	Vitek 2	CLSI	93
Liu L (2017)[125]	2004-2012	China	*salmonella*	food	65	Microdilution broth method	CLSI	11
Liu SQ (2020)[126]	2017-2018	USA	*Escherichia coli*	meat	104	Kirby-Bauer	CLSI	95
Liu WJ (2020)[127]	2017-2019	China	*Vibrio parahemolyticus*	disease	15	Microdilution broth method	CLSI	10
Liu Y (2018)[128]	2016	China	*salmonella*	raw meat	158	Microdilution broth method	NARMS	158
Long ZD (2020)[129]	2018-2019	China	*Vibrio parahemolyticus*	aquatic products	104	Kirby-Bauer	CLSI	101

Study	Period	Location	Pathogenic Bacteria	Source	Strains (n)	Susceptibility Test	Guidelines	Resistance strains (n)
Lotapak M (2015)[130]	2009-2012	Poland	*Vibrio parahemolyticus*	shellfish	64	Microdilution broth method	CLSI	56
Lu QY (2020)[131]	2018	China	*Vibrio parahemolyticus*	diarrheal disease	58	Microdilution broth method		37
Lu Y (2016)[132]	—	China	*Vibrio parahemolyticus*	aquatic products	45	Kirby-Bauer	NCCLS	45
Lv GP (2013)[133]	2010-2011	China	*staphylococcus aureus*	food poisoning<br>food	14<br>61	Disk diffusion test	CLSI	13<br>60
Lv SL (2010)*	2006-2007	China	*staphylococcus aureus*	milk	45	Vitek 2	GPS-110	42
Lv SL (2012)*	2010	China	*staphylococcus aureus*	food	118	Reagent test kit	—	105
Ma ZL (2011)[134]	2006-2010	China	*salmonella*<br>*Listeria monocytogenes*	food	33<br>50	Disk diffusion test	CLSI	33<br>50
Matallah AM (2019)[135]	2016-2017	Algeria	*staphylococcus aureus*	milk	95	Disk diffusion test	CLSI	52
Mezali L (2012)[136]	2007-2008	Algeria	*salmonella*	meat	64	Disk diffusion test	CLSI	56
Nadi ZR (2020)[137]	2013-2019	Iran	*salmonella*	food-borne diarrhea patients	80	Disk diffusion test	CLSI	46
Nemati V (2020)[138]	2018	Iran	*Listeria monocytogenes*	RET-food	100	Microdilution broth method	CLSI	37
Niu LY (2017)[139]	2011-2016	China	*salmonella*	food poisoning	40	Vitek 2	CLSI	40
Obaidat MM (2015)[140]	—	Jordan	*staphylococcus aureus*	fish	156	Disk diffusion test	CLSI	135
Obaidat MM (2015)[141]	—	Jordan	*Listeria monocytogenes*	fish	104	Disk diffusion test	CLSI	104
Oh EG (2011)[142]	2005-2006	Korea	*Vibrio parahemolyticus*	fishes	218	Disk diffusion test	CLSI	142
Ottaviani D (2013)[143]	2009-2011	Italy	*Vibrio parahemolyticus*	Shellfish	87	Kirby-Bauer	CLSI	86
Park KM (2018)[144]	2015	Korea	*Bacillus cereus*	food	118	Disk diffusion test	CLSI	118

Study	Period	Location	Pathogenic Bacteria	Source	Strains (n)	Susceptibility Test	Guidelines	Resistance strains (n)
Pazhani GP (2014)[145]	2001-2012	India	*Vibrio parahemolyticus*	diarrheal disease	178	Disk diffusion test	CLSI	174
Pen SP (2020)[146]	2018-2019	China	*salmonella*	diarrhea patients	366	Microdilution broth method	CLSI	294
Pu SH (2011)[147]	—	USA	*staphylococcus aureus*	food	152	Microdilution broth method	CLSI	151
Puig-Pena Y (2020)[148]	2004-2018	Cuba	*Escherichia coli*	food	381	Disk diffusion test	CLSI	220
			salmonella		402			236
Pungpian C (2020)[149]	2013-2018	Thailand	*Escherichia coli*	clinical samples	70	Microdilution broth method	CLSI	45
		Lao PDR			80			48
		Lao PDR		pork	104			95
		Thailand			112			84
Qi QQ (2020)[150]	2012-2018	China	*salmonella*	diarrhea patients	84	BD Phoenix	CLSI	83
Qin S (2020)[151]	2018-2019	China	*Escherichia coli*	diarrheal disease	540	Agar dilution	CLSI	490
Qin S (2020)[152]	2016-2019	China	*Escherichia coli*	foodborne diseases	104	Agar dilution	CLSI	74
Qu Mei (2020)[153]	2019-(2020)	China	*Vibrio parahemolyticus*	diarrheal disease	1853	Kirby-Bauer	CLSI	1428
Que FX (2020)[154]	2017-2018	China	*Vibrio parahemolyticus*	aquatic products	54	Minimal broth	CLSI	34
Quino W (2020)[155]	2012-2015	Peru	*salmonella*	clinical samples	520	Kirby-Bauer	CLSI	494
Ramadan H (2020)[156]	2017	Egypt	*Escherichia coli*	foodborne diseases	32	Microdilution broth method	CLSI	31
		Egypt	*Escherichia coli*	meat	88	Microdilution broth method	CLSI	72
Ranjbar R (2018)[157]	2015-2016	Iran	*Escherichia coli*	milk dairy product	64	Disk diffusion test	CLSI	64
Richter (2019)[158]	2017-2018	South Africa	*Escherichia coli*	vegetables	67	Disk diffusion test	CLSI	23
Romero-Barrios P	2012-2013	Canadian	*Escherichia coli*	broiler	1135	Microdilution broth method	—	940

Study	Period	Location	Pathogenic Bacteria	Source	Strains (n)	Susceptibility Test	Guidelines	Resistance strains (n)
(2020)[159]			*salmonella*		1495			879
Rong DL (2017)[160]	2015-2016	China	*staphylococcus aureus*	fish	119	Disk diffusion test	CLSI	105
Ryu AR (2019)[161]	2016	Korea	*Vibrio parahemolyticus*	bivalves	288	Disk diffusion test	CLSI	233
Ryu AR (2017)[162]	2013	Korea	*Vibrio parahemolyticus*	aquatic products	194	—	—	87
Sadr MM (2019)[163]	2017	Iran	*staphylococcus aureus*	traditional sweet	79	Disk diffusion test	CLSI	69
Savariraj WR (2019)[164]	—	India	*staphylococcus aureus*	pork	92	Disk diffusion test	EUCAST	92
Sha WJ (2018)*	—	China	*Vibrio parahemolyticus*	aquatic products	35	Kirby-Bauer	CLSI	33
Sharma SK (2020)[165]	—	India	*staphylococcus aureus*	clinical samples	35	Disk diffusion test	CLSI	35
Shen JL (2020)[166]	2017-2019	China	*Vibrio parahemolyticus*	aquatic products	68	Disk diffusion test	CLSI	67
Shen Y (2020)[167]	2019	China	*salmonella*	food-borne diarrhea patients	105	Vitek 2 Compact	—	104
Shen YH (2020)[168]	2017	China	*Vibrio parahemolyticus*	disease	65	Minimal broth dilution	—	41
Shen Z (2018) *	2016	China	*salmonella*	food borne diseases	287	Microdilution broth method	CLSI	186
Shi CP (2020)[169]	2016	China	*staphylococcus aureus*	milk	276	Kirby-Bauer	CLSI	261
Shi W (2015)[170]	2012-2014	China	*Listeria monocytogenes*	RET-food	248	Kirby-Bauer	CLSI	116
Silveira-filho VM (2014)[171]	—	Brazil	*staphylococcus aureus*	milk	94	Disk diffusion test	CLSI	84
Skowron K (2020)[172]	2016-2018	Poland	*Listeria monocytogenes*	meat	127	Disk diffusion test	CLSI	55
Parvin MS (2020)[173]	2019	Bangladesh	*Escherichia coli*	meat	86	Disk diffusion test	CLSI	86
Su CL (2020)[174]	2017	China	*Vibrio parahemolyticus*	aquatic products	561	Disk diffusion test	CLSI	521
Su J (2017)[175]	2015	China	*staphylococcus aureus*	food	49	Microdilution broth method	CLSI	36
Su JH (2012)[176]	2010	China	*Vibrio parahemolyticus*	food poisoning	59	Kirby-Bauer	CLSI	58

Study	Period	Location	Pathogenic Bacteria	Source	Strains (n)	Susceptibility Test	Guidelines	Resistance strains (n)
				cases				
				aquatic products	101			99
Ta YT (2014)[177]	—	USA	*salmonella*	meat	457	disk agar diffusion test	CLSI	335
Tan CW (2020)[178]	2018	Malaysia	*Vibrio parahemolyticus*	aquatic products	120	Disk diffusion test	CLSI	119
Tan CW (2017)[179]	2016	Malaysia	*Vibrio parahemolyticus*	fish	67	Disk diffusion test	CLSI	62
Tang J (2020)[180]	2018	China	*salmonella*	diarrhea patients	53	Disk diffusion test	CLSI	52
Tang YX (2017)[181]	2015	China	*Vibrio parahemolyticus*	aquatic products	108	Disk diffusion test	NCCLS	105
Tang Z (2018)[182]	2015-2017	China	*Vibrio parahemolyticus*	aquatic products	267	Minimal broth	CLSI	255
Tang Z (2018)[183]	2017	China	*Escherichia coli*	diarrheal disease	266	broth dilution method	CLSI	243
Tadesse HA (2018)[184]	2016-2017	Ethiopia	*Escherichia coli*	milk	115	Disk diffusion test	CLSI	80
Temelli S (2012)[185]	2006-2009	Turkey	*salmonella*	chicken carcasses	64	Agar dilution	CLSI	64
Torpdahl M (2013)[186]	2009-2010	taiwan<br>Denmark	*salmonella*	clinical samples	185<br>158	Microdilution broth method	CLSI	158<br>75
Touimi GB (2020)[187]	2015-2016	Morocco	*staphylococcus aureus*	food	52	Disk diffusion test	CLSI	52
Uysal A (2012)[188]	2009-2010	Turkey	*Escherichia coli*	food	40	Disk diffusion test	CLSI	40
Vega-Sánchez V (2020)[189]	2013-2014	Mexico	*salmonella*	pork	37	Disk diffusion test	CLSI	27
Viana C (2020)[190]	—	Brazil	*salmonella*	pork	41	Microdilution broth method	CLSI	41
Wang D (2014)[191]	2010-2012	China	*staphylococcus aureus*	food	187	Microdilution broth method	CLSI	116
Wang G (2020)[192]	—	China	*Vibrio parahemolyticus*	aquatic products	15	Minimal broth	CLSI	15
Wang HL (2017)[193]	2013-2014	China	*Vibrio parahemolyticus*	diarrheal disease	107	Microdilution broth method	CLSI	91
Wang J (2013)[194]	—	China	*Vibrio parahemolyticus*	food poisoning cases	35	Kirby-Bauer	CLSI	35
Wang JW (2019)[195]	2015-2016	China	*salmonella*	meat	219	Agar dilution	CLSI	219

Study	Period	Location	Pathogenic Bacteria	Source	Strains (n)	Susceptibility Test	Guidelines	Resistance strains (n)
Wang L (2011)[196]	2007-2010	China	*salmonella*	food	150	Microdilution broth method	CLSI	126
Wang LL (2017)[197]	2005-2008	Japan	*Escherichia coli*	food	82	Disk diffusion test	CLSI	50
Wang LL (2016)[198]	2013-2015	China	*Listeria monocytogenes*	patients	37	E-test	EUCAST	31
Wang LP (2019)[199]	2014-2017	China	*salmonella*	food	91	Microdilution broth method	CLSI	68
Wang P (2017)[200]	2014-2016	China	*Vibrio parahemolyticus*	diarrheal disease	125	Disk diffusion test	CLSI	125
Wang QY (2012)[201]	2009-2011	China	*Vibrio parahemolyticus*	diarrheal disease	45	Microdilution broth method	—	26
Wang W (2017)[202]	2015	China	*staphylococcus aureus*	food	1150	broth dilution method	CLSI	1122
Wang W (2019)[203]	2018	China	*staphylococcus aureus*	meat	32	Disk diffusion test	CLSI	30
			Escherichia coli		32			14
			salmonella		32			15
Wang W (2020)[204]	2017	China	*staphylococcus aureus*	RTE food	32	Microdilution broth method	CLSI	32
wang W (2016)[205]	2014	China	*salmonella*	chicken carcasses	355	Microdilution broth method	CLSI	321
Wang WX (2011)[206]	2009-2010	China	*salmonella*	pork	56	Agar plate dilution method	CLSI	52
Wang XH (2012)*	2011	China	*staphylococcus aureus*	food poisoning	11	Kirby-Bauer	CLSI	11
Wang XL (2020)[207]	2016-2019	China	*Vibrio parahemolyticus*	diarrheal disease	118	Minimal broth dilution	NCF	105
				food	36			19
Wang YB (2018)[208]	2016	China	*Vibrio parahemolyticus*	diarrheal disease	39	Minimal broth dilution	CLSI	39
Wang ZE (2019)[209]	2017	China	*Vibrio parahemolyticus*	diarrheal disease	34	Minimal broth dilution	CLSI	31
Wei Q (2016)[210]	2011-2015	China	*salmonella*	foodborne diseases	70	Microdilution broth method	CLSI	70
Wieczorek K (2017)[211]	2014-2016	Poland	*Listeria monocytogenes*	fish	57	Disk diffusion test	CLSI	17
Wu NW (2012)[212]	—	China	*Vibrio parahemolyticus*	food poisoning cases	30	Kirby-Bauer	CLSI	30

Study	Period	Location	Pathogenic Bacteria	Source	Strains (n)	Susceptibility Test	Guidelines	Resistance strains (n)
Wu Q (2015)[213]	—	China	*Vibrio parahemolyticus*	aquatic products	80	Microdilution broth method	CLSI	50
Wu S (2019)[214]	2011-2016	China	*staphylococcus aureus*	food	72	Kirby-Bauer	CLSI	72
Wu S (2014)[215]	2012-2014	China	*Listeria monocytogenes*	RET food	80	Kirby-Bauer	CLSI	80
Wu XF (2018)[216]	2013-2016	China	*salmonella*	food borne diseases	73	Microdilution broth method	CLSI	72
Wu ZF (2015)*	2013-2014	China	*Vibrio parahemolyticus*	diarrheal disease	47	—	—	26
Xia D (2017)[217]	2014-2015	China	*staphylococcus aureus*	food poisoning	44	Vitek 2	—	35
Xie AR (2017)[218]	—	China	*staphylococcus aureus*	food	100	Disk diffusion test	CLSI	92
Xie TF (2017)[219]	2014	China	*Vibrio parahemolyticus*	diarrheal disease	95	Disk diffusion test	CLSI	29
				aquatic products	31			86
Xie TF (2015)[220]	2013-2014	China	*Vibrio parahemolyticus*	aquatic products	150	Disk diffusion test	CLSI	133
Xie TF (2016)[221]	2011-2014	China	*Vibrio parahemolyticus*	RET food	39	Disk diffusion test	CLSI	39
Xing Y (2016)[222]	2013-2015	China	*salmonella*	foodborne diseases	75	Vitek 2	CLSI	64
Xu B (2017)[223]	2013-2016	China	*Vibrio parahemolyticus*	diarrheal disease	35	Kirby-Bauer	CLSI	31
Xu FF (2011)[224]	2007-2010	China	*Vibrio parahemolyticus*	diarrheal disease	193	Kirby-Bauer	NCCLS	123
Xu HY (2019)[225]	2012-2017	China	*salmonella*	food practitioner	164	—	—	116
Xu J (2014)[226]	2009-2012	China	*staphylococcus aureus*	food	78	Disk diffusion test	CLSI	78
Xu JY (2015)[227]	2008-2014	China	*Vibrio parahemolyticus*	diarrheal disease	530	Kirby-Bauer	CLSI	520
Xu JY (2015)[228]	—	China	*Vibrio parahemolyticus*	food	760	Kirby-Bauer	CLSI	386
Xu QF (2020)[229]	2015-2016	China	*Vibrio parahemolyticus*	diarrheal disease	296	broth dilution	CLSI	285
Xu XK (2016)[230]	2012-2013	China	*Vibrio parahemolyticus*	aquatic products	145	Disk diffusion test	CLSI	125
Xu ZH (2020)[231]	2009-2016	China	*salmonella*	raw meat	579	Kirby-Bauer	CLSI	507
Yan H (2010)[232]	2005-2007	China	*Listeria monocytogenes*	food	90	Disk diffusion test	CLSI	49

Study	Period	Location	Pathogenic Bacteria	Source	Strains (n)	Susceptibility Test	Guidelines	Resistance strains (n)
Yan HQ (2015)[233]	2011-2014	China	*Vibrio parahemolyticus*	diarrheal disease	104	Microdilution broth method	CLSI	98
				aquatic products	32			26
Yan SF (2014)[234]	2012	China	*Listeria monocytogenes*	food	635	Minimal broth method	CLSI	66
Yang H (2020)[235]	—	China	*Escherichia coli*	eady-to-eat duck products	109	Vitek 2	CLSI	109
Yang Juan (2016)[236]	2012-2014	China	*Vibrio parahemolyticus*	aquatic products	38	Kirby-Bauer	CLSI	22
Yang JX (2019)[237]	2016-2017	China	*salmonella*	RET-food	147	Agar dilution	CLSI	138
Yang LH (2010)[238]	2007-2009	China	*Vibrio parahemolyticus*	food	38	Kirby-Bauer	CLSI	34
Yang XJ (2019)[239]	2011-2016	China	*salmonella*	meat	209	Disk diffusion test	CLSI	181
Yang XJ (2016)[240]	2011-2014	China	*salmonella*	RET-food	54	Disk diffusion test	CLSI	37
Yang XW (2015)[241]	2006-2012	China	*salmonella*	clinical samples	88	Kirby-Bauer	CLSI	47
Yang Y (2017)[242]	2015-2017	China	*Vibrio parahemolyticus*	aquatic products	98	Disk diffusion test	CLSI	78
Yang YJ (2015)[243]	—	China	*Vibrio parahemolyticus*	aquatic products	53	Minimal broth dilution	—	53
Yano Y (2014)[244]	2007-2009	Thailand	*Vibrio parahemolyticus*	shrimps	70	Microdilution broth method	CLSI	52
Yao Y (2019)[245]	2013-2017	China	*Vibrio parahemolyticus*	foodbrone	44	Vitek 2	CLSI	37
			salmonella	diarrheal disease	78			60
Yin BK (2012)[246]	—	China	*staphylococcus aureus*	food	42	Kirby-Bauer	CLSI	38
Yim JH (2015) [247]	2013	Korea	*Bacillus cereus*	soybean products	87	Disc diffusion test	CLSI	87
Yoon RH (2014)[248]	2013	Korea	*salmonella*	duck meat	64	Disc diffusion test	CLSI	64
You YX (2020)[249]	2018	China	*salmonella*	diarrhea patients	58	Microdilution broth method	CLSI	45
Yu JM (2014)[250]	—	China	*Vibrio parahemolyticus*	aquatic products	129	Kirby-Bauer	CLSI	127
Yu PF (2019)[251]	2011-2016	China	*Bacillus cereus*	vegetables	294	Kirby-Bauer	CLSI	293
Yu QQ (2016)[252]	2014-2015	China	*Vibrio parahemolyticus*	aquatic products	96	Agar dilution	CLSI	85
Yuan DK (2013)[253]	2009-2010	China	*Vibrio parahemolyticus*	food poisoning	79	Kirby-Bauer	NCCLS	77

Study	Period	Location	Pathogenic Bacteria	Source	Strains (n)	Susceptibility Test	Guidelines	Resistance strains (n)
				cases				
Yuan M (2014)[254]	—	China	*Escherichia coli*	food	288	Microdilution broth method	NCCLS	288
Yuan PZ (2016)[255]	2012-2014	China	*salmonella*	foodborne diseases	35	Kirby-Bauer	CLSI	31
Yuan Y (2013)[256]	2011-2012	China	*Vibrio parahemolyticus*	aquatic products	65	Kirby-Bauer	CLSI	65
Yuan ZH (2019)[257]	2017	China	*salmonella*	food borne diseases	285	Microdilution broth method	CLSI	237
Yue M (2020)[258]	2013-2015	China	*salmonella*	foodborne diarrhea patients	61	Kirby-Bauer	CLSI	39
Zanella GN (2011)[259]	2006-2007	Brazil	*Escherichia coli*	milk	47	Disc diffusion test	CLSI	11
Zeng XY (2018)[260]	2016	China	*salmonella*	food borne diseases	234	Microdilution broth method	CLSI	164
				food	70			32
Zeng YB (2019)[261]	2010-2014	China	*salmonella*	meat	92	Disk diffusion test	CLSI	92
Zhang J (2018)[262]	2010-2016	China	*staphylococcus aureus*	food	118	Vitek 2	—	84
Zhang JQ (2020)[263]	2014-2018	China	*Vibrio parahemolyticus*	diarrheal disease	107	Kirby-Bauer	CLSI	107
Zhang JQ (2010)[264]	2007-2008	China	*Vibrio parahemolyticus*	diarrheal disease	43	Disk diffusion test	CLSI	25
Zhang LH (2014)[265]	2012-2013	China	*salmonella*	foodborne diseases	88	Kirby-Bauer	CLSI	43
Zhang MY (2020)[266]	—	China	*salmonella*	food-borne diarrhea patients	54	—	—	28
Zhang SH (2016)[267]	2013-2014	China	*Escherichia coli*	RET-food	36	Agar dilution	CLSI	31
Zhang SH (2014)[268]	2005-2013	China	*Listeria monocytogenes*	RET-food	136	Minimal broth method	CLSI	63
Zhang SY (2016)*	2013	China	*staphylococcus aureus*	food	31	Vitek 2	—	31

Study	Period	Location	Pathogenic Bacteria	Source	Strains (n)	Susceptibility Test	Guidelines	Resistance strains (n)
Zhang XY (2020)[269]	2019	China	*Listeria monocytogenes*	food	56	Minimal broth method	CLSI	11
Zhang Y (2020)[270]	2011-2016	China	*Bacillus cereus*	food	58	Kirby-Bauer	CLSI	58
Zhang Y (2011)[271]	2010	China	*Listeria monocytogenes*	food	36	Kirby-Bauer	CLSI	15
Zhang YY (2019)[272]	2016	China	*Listeria monocytogenes*	food	73	Microdilution broth method	CLSI	64
Zhang ZF (2018)[273]	2008-2012	China	*salmonella*	clinical samples<br>food	77<br>64	Microdilution broth method	CLSI	76<br>64
Zhang ZY (2015)[274]	2014	China	*Vibrio parahemolyticus*	aquatic products	40	Microdilution broth method	CLSI	37
Zhao D (2020)[275]	2018	China	*staphylococcus aureus*	food	41	Kirby-Bauer	CLSI	32
Zhao H (2011)*	2009	China	*Vibrio parahemolyticus*	diarrheal disease	31	Kirby-Bauer	NCCLS	29
Zhao SJ (2020)[276]	2018-2019	China	*Bacillus cereus*	dairy products	54	Kirby-Bauer	CLSI	52
Zhao Y (2012)[277]	2007-2009	China	*Listeria monocytogenes*	food	1069	Minimal broth method	CLSI	74
Zhen ZB (2020)[278]	2017-2019	China	*salmonella*	foodborne diarrhea patients	339	microdilution broth	CLSI	277
Zheng WL (2015)[279]	—	China	*Vibrio parahemolyticus*	diarrheal disease	79	Kirby-Bauer	CLSI	8
Zheng WL (2015)[280]	—	China	*Vibrio parahemolyticus*	food	79	Microdilution broth method	CLSI	79
Zhou HD (2018)[281]	2016-2017	China	*salmonella*	food borne diseases	146	Microdilution broth method	CLSI	146
Zhou HZ (2010)[282]	2009	China	*Vibrio parahemolyticus*	aquatic products	56	Kirby-Bauer	—	31
Zhou Q (2016)[283]	2014-2015	China	*Vibrio parahemolyticus*	aquatic products	36	Vitek 2	—	24
Zhou Q (2019)[284]	2015-2017	China	*Bacillus cereus*	vegetable	60	Kirby-Bauer	CLSI	60
Zhou WY(2016)[285]	2011-2014	China	*Vibrio parahemolyticus*	diarrheal disease	1100	Kirby-Bauer	CLSI	956
Zhou Y (2014)[286]	2012	China	*salmonella*	foodborne diseases	76	Kirby-Bauer	CLSI	66
Zhou YQS (2019)[287]	2015-2018	China	*salmonella*	food	337	Microdilution broth method	CLSI	—

**Study**	**Period**	**Location**	**Pathogenic Bacteria**	**Source**	**Strains (n)**	**Susceptibility Test**	**Guidelines**	**Resistance strains (n)**
Zhou ZY (2020) *	2017	China	*salmonella*	diarrhea patients	87	—	CLSI	74
Zhu AH (2019)[288]	2016-2017	China	*salmonella*	pork	115	Disk diffusion test	CLSI	112
Zhu JH (2020) [289]	2015-2017	China	*Vibrio parahemolyticus*	diarrheal disease	72	Kirby-Bauer	CLSI	71
Zhu XJ (2017) [290]	2013-2015	China	*Vibrio parahemolyticus*	diarrheal disease	42	Vitek 2	NCCLS	40
				aquatic products	164			160
Zhuge SY (2014) [291]	—	China	*Bacillus cereus*	aquatic products	59	Microdilution broth method	Guidebook	—
Zhuge SY (2015) [292]	2010-2013	China	*staphylococcus aureus*	quick frozen flour product	86	Microdilution broth method	—	86
Zhuo WL (2016) [293]	2011-2014	China	*Vibrio parahemolyticus*	diarrheal disease	1100	Kirby-Bauer	CLSI	965

Notice: * Articles are in Chinese without English titles.

Table 3. Characteristics of included biofilm studies.

Study	Period	Location	Pathogenic Bacteria	Source	Number of strains	Method	Biofilm strains (n)	Strong (n)	Moderate (n)	Weak (n)
Beshiru A (2018a)[294]	2016-2017	Nigeria	*Vibrio parahemolyticus*	shrimp	46	Quantitative microtiter assay	44	—	—	—
Beshiru A (2018b)[295]	2016-2017	Nigeria	*salmonella*	shrimp	45	Quantitative microtiter assay	45	0	25	20
Chen Q (2019)[296]	—	China	*staphylococcus aureus*	food	97	Quantitative microtiter assay	70	3	14	53
kim HJ (2018)[297]	2006-2013	Korea	*Escherichia coli*	food	67	Quantitative microtiter assay	66	30	36	0
Lapierre L (2020)[103]	—	Chile	*salmonella*	meat	87	Quantitative microtiter assay	82	1	0	81
Lopez-Leon P (2016)[298]	—	Mexico	*Vibrio parahemolyticus*	shrimp	35	Quantitative microtiter assay	35	5	30	0
Maia DSV (2020)[299]	2017	Brazil	*staphylococcus aureus*	food	13	Quantitative microtiter assay	13	—	—	—
Ou CJ (2020)[300]	2018-2019	China	*staphylococcus aureus*	food	165	Quantitative microtiter assay	165	107	33	25
Puah SM (2018)[301]	2014	Malaysia	*staphylococcus aureus*	fish	52	Quantitative microtiter assay	52	23	17	12
Rodríguez-Lázaro D (2018)[302]	—	Spain	*staphylococcus aureus*	food	49	Quantitative microtiter assay	49	8	41	0
Wang W (2019)[203]	2018	China	*staphylococcus aureus*<br>*Escherichia coli*<br>*salmonella*	meat	32<br>32<br>32	Quantitative microtiter assay	13<br>16<br>14	3<br>0<br>0	10<br>5<br>4	19<br>11<br>10

Table 4. Quality Assessment of Included Studies

	Yes	No	Unclear
**Assessment scale for food studies (n=244):**			
1.Was the study purpose clearly clear?	244(100%)	-	-
2.Were randomly select the study subjects?	77(32%)	15(6%)	152(62%)
3.Were the criteria for inclusion in the sample clearly defined?	185(76%)	10(4%)	49(20%)
4.Was the sample size sufficiently large to provide confidence in the findings?	163(67%)	13(5%)	68(28%)
5.Were the study subjects and the setting described in detail?	190(78%)	39(16%)	15(6%)
6.Were the outcomes measured in a valid and reliable way?	244(100%)	-	
7.Were objective, standard criteria used for measurement of the condition?	234(96%)	4(2%)	6(2%)
8.Was appropriate statistical analysis used?	110(45%)	96(39%)	38(16%)
9.Were the results stated in adequacy?	236(97%)	2(1%)	6(2%)
10.Was the research value described in clear?	244(100%)	-	-
**Assessment scale for human studies (n=104):**			
1.Was the sample frame appropriate to address the target population?	104(100%)	-	-
2.Were study participants sampled in an appropriate way?	71(68%)	2(2%)	31(30%)
3.Was the sample size adequate?	70(67%)	19(18%)	15(14%)
4.Were the study subjects and the setting described in detail?	53(51%)	45(43%)	6(6%)
5.Was the data analysis conducted with sufficient coverage of the identified sample?	88(85%)	1(%)	15(14%)
6.Were valid methods used for the identification of the condition?	93(89%)	1(1%)	10(10%)
7.Was the condition measured in a standard, reliable way for all participants?	94(90%)	3(3%)	7(7%)
8.Was there appropriate statistical analysis?	79(76%)	13(12%)	12(12%)
9.Was the response rate adequate, and if not, was the low response rate managed appropriately?	104(100%)	-	-

Supplementary forest plots

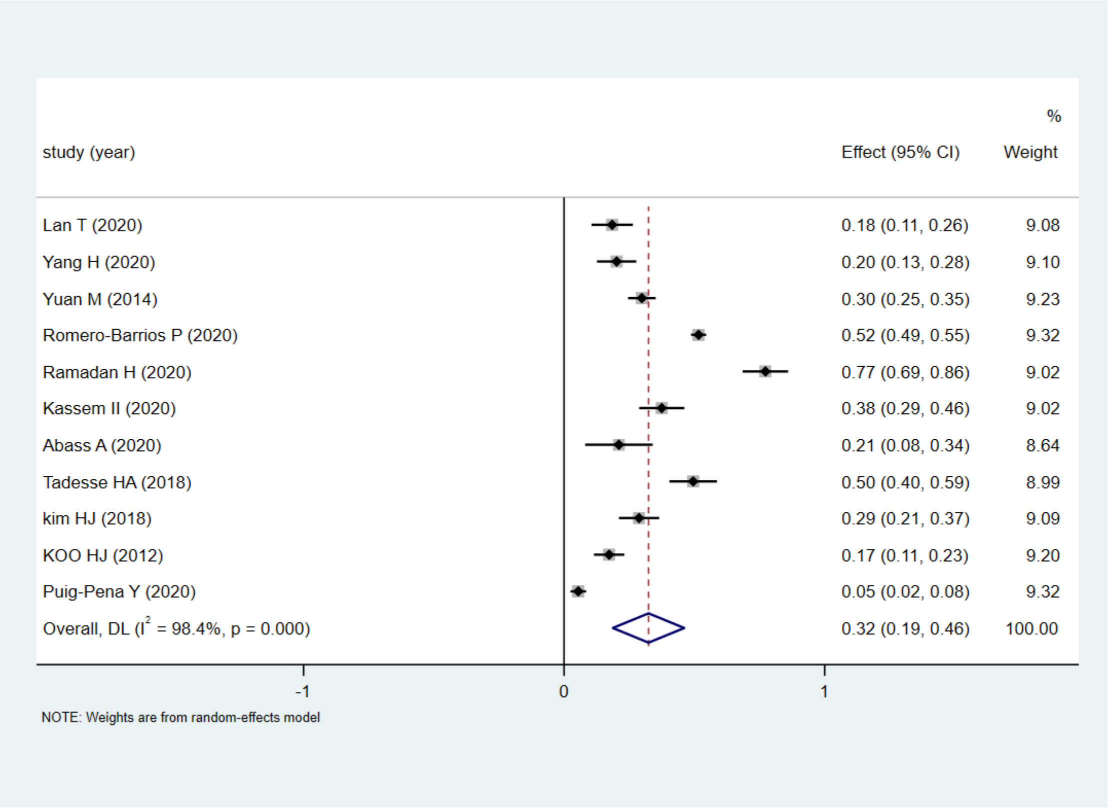

Figure 1. Aminoglycosides resistance in *Escherichia coli* isolates from food

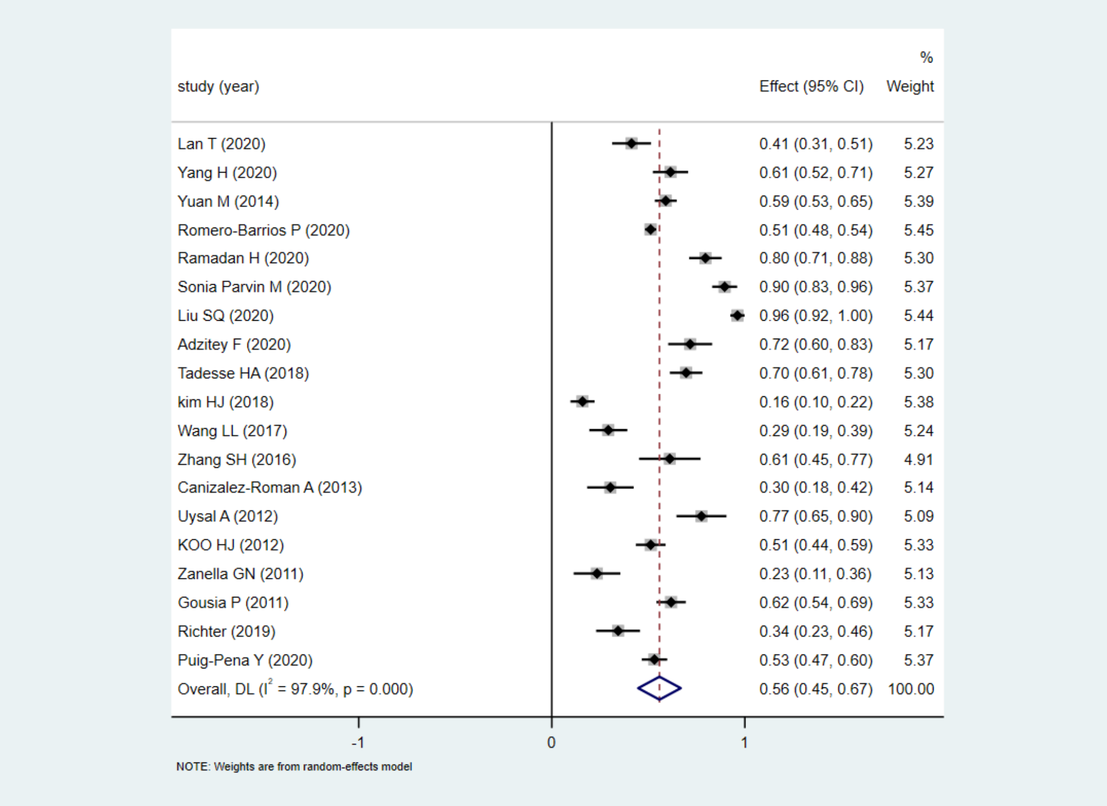

Figure 2. β-Lactams resistance in *Escherichia coli* isolates from food

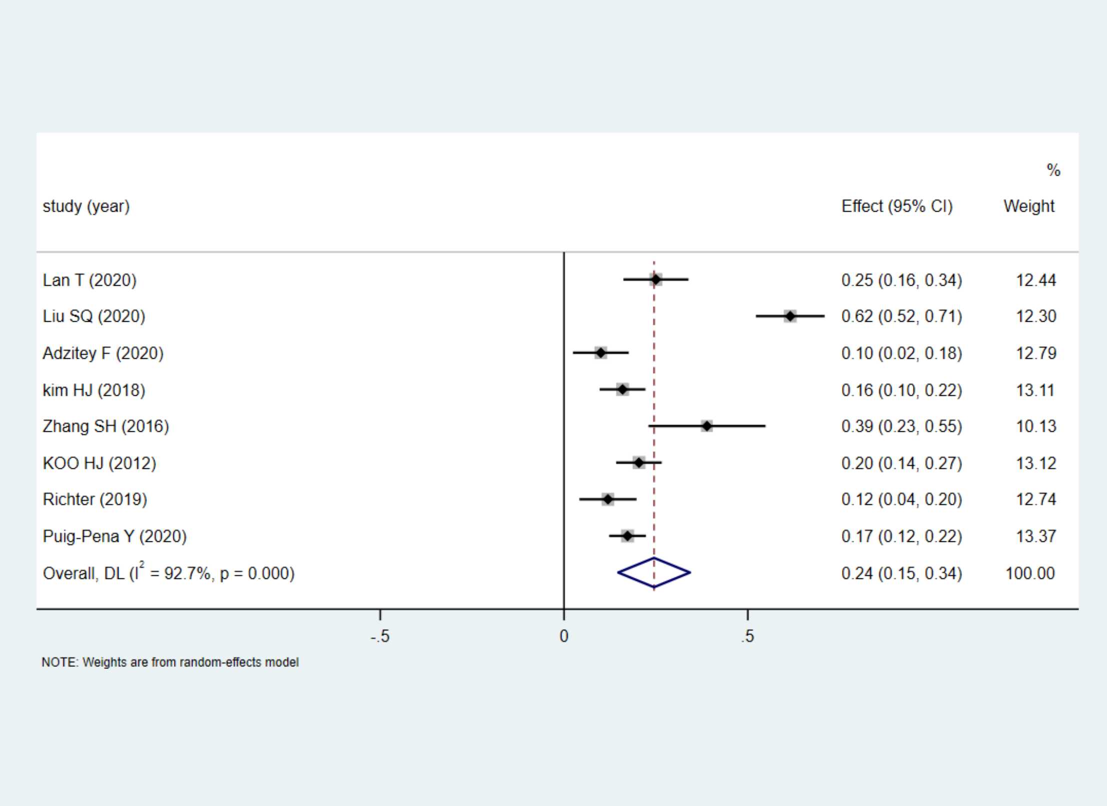

Figure 3. Chloramphenicol resistance in *Escherichia coli* isolates from food

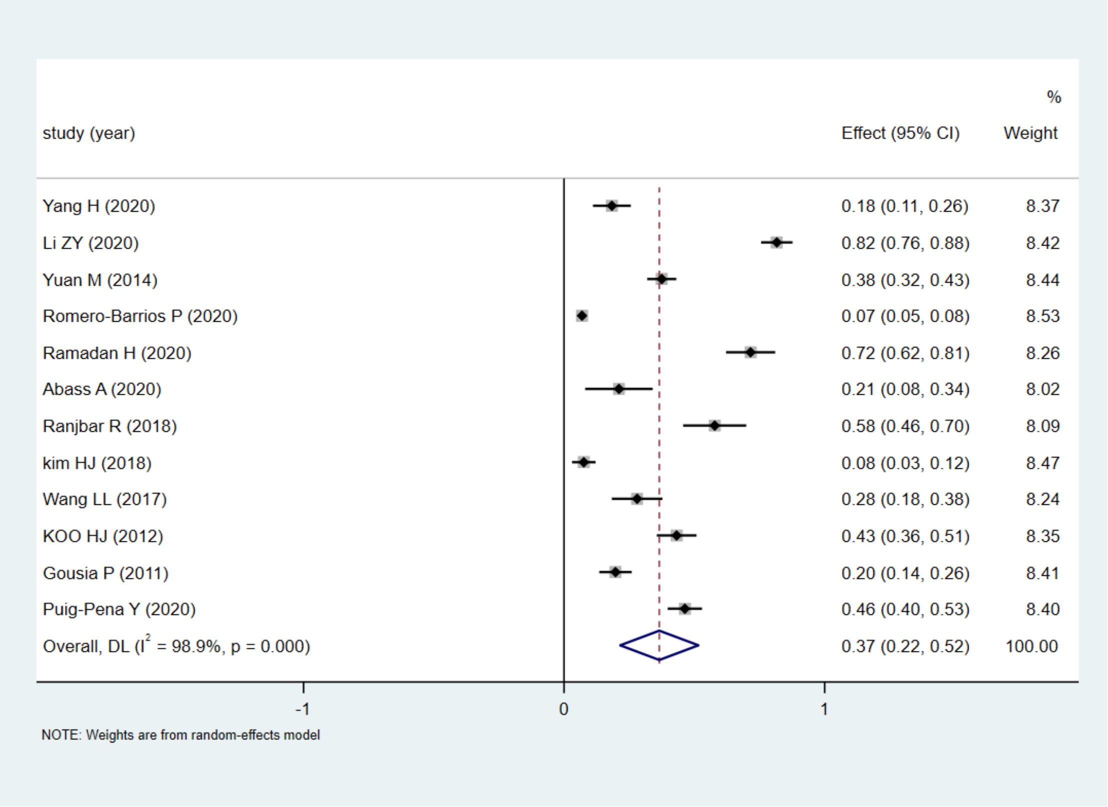

Figure 4. Fluoroquinolones resistance in *Escherichia coli* isolates from food

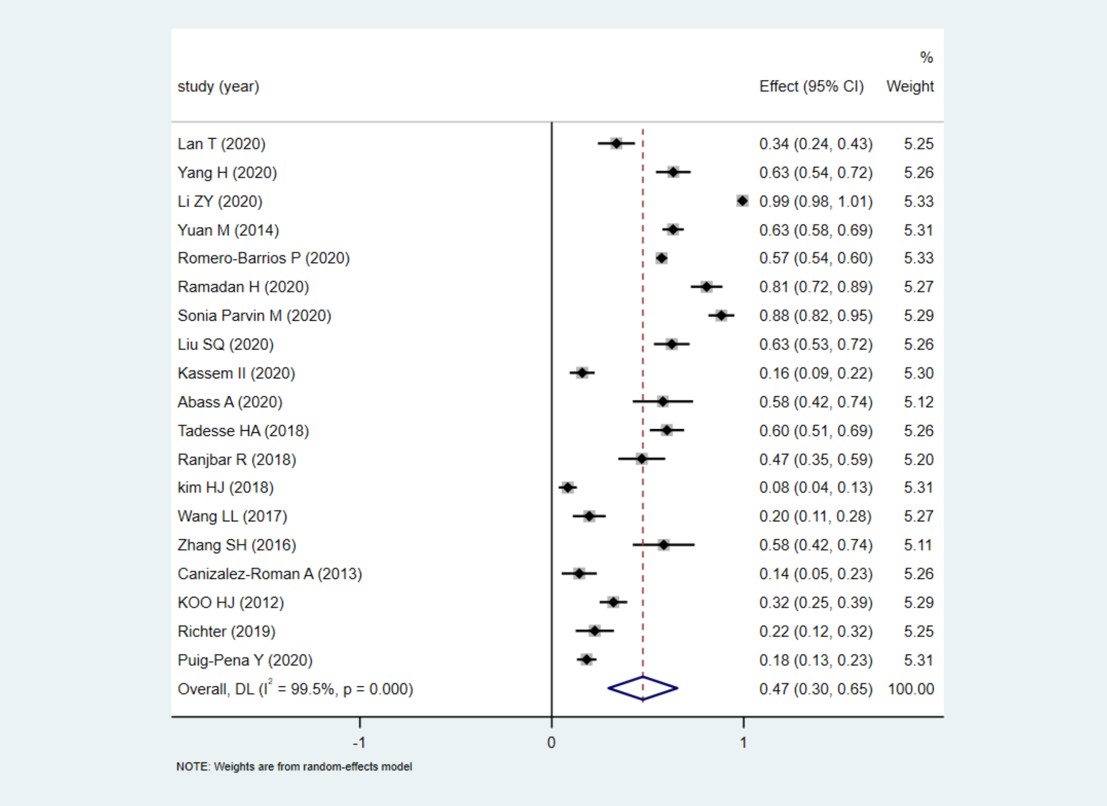

Figure 5. Sulfonamides resistance in *Escherichia coli* isolates from food

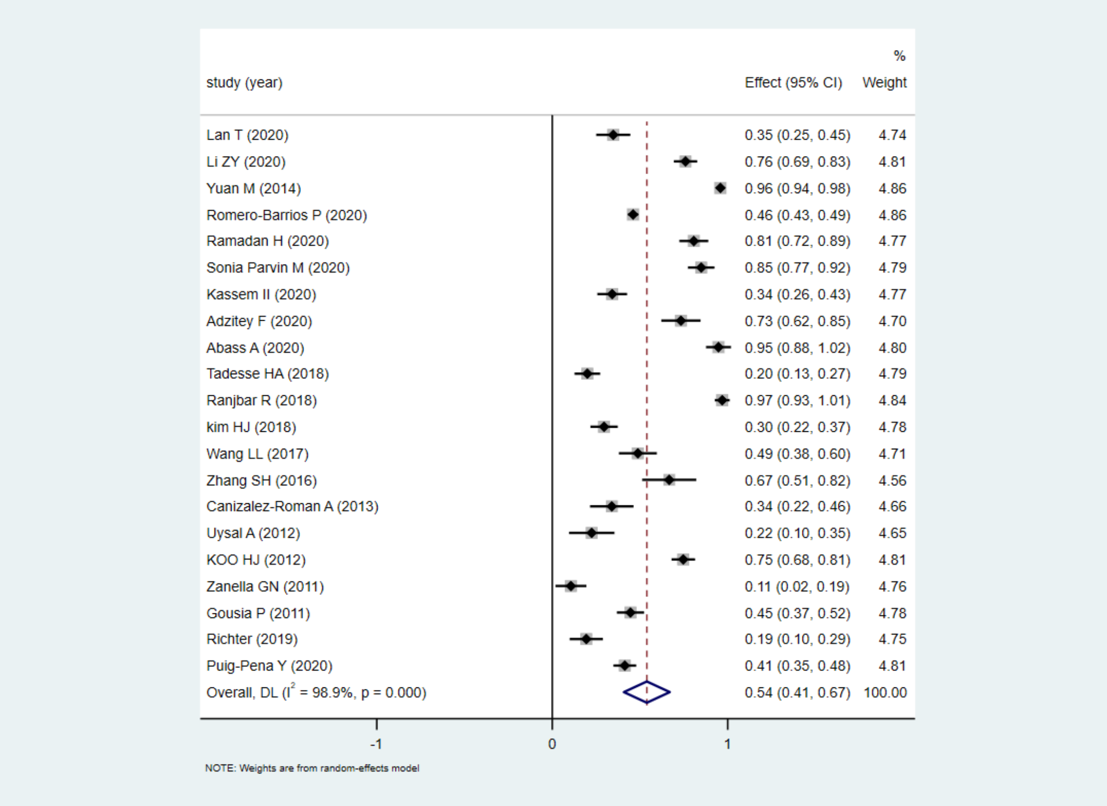

Figure 6. Tetracyclines resistance in *Escherichia coli* isolates from food

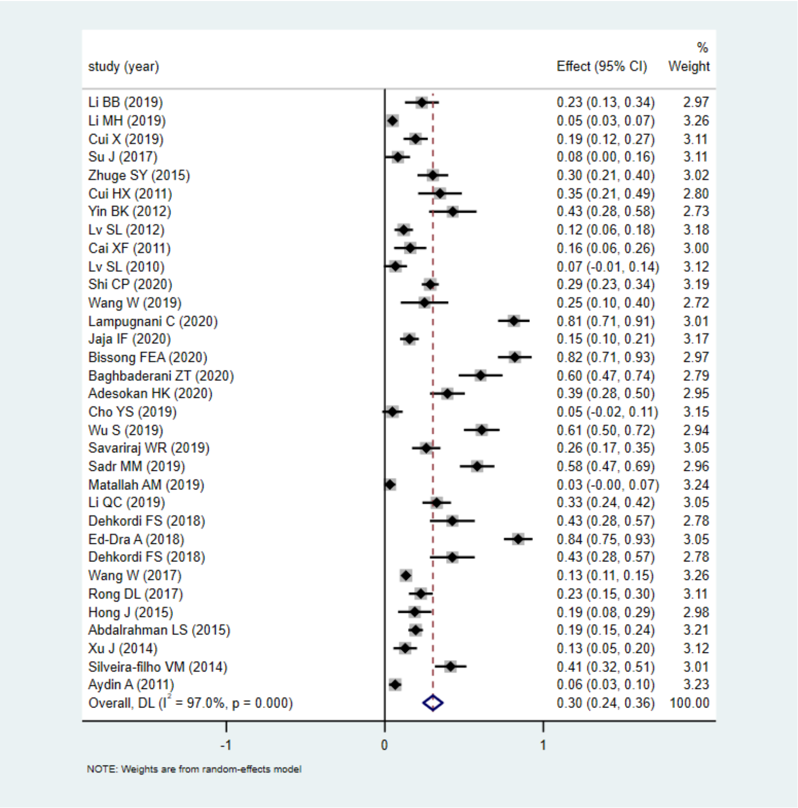

Figure 7. Aminoglycosides resistance in Staphylococcus aureus isolates from food

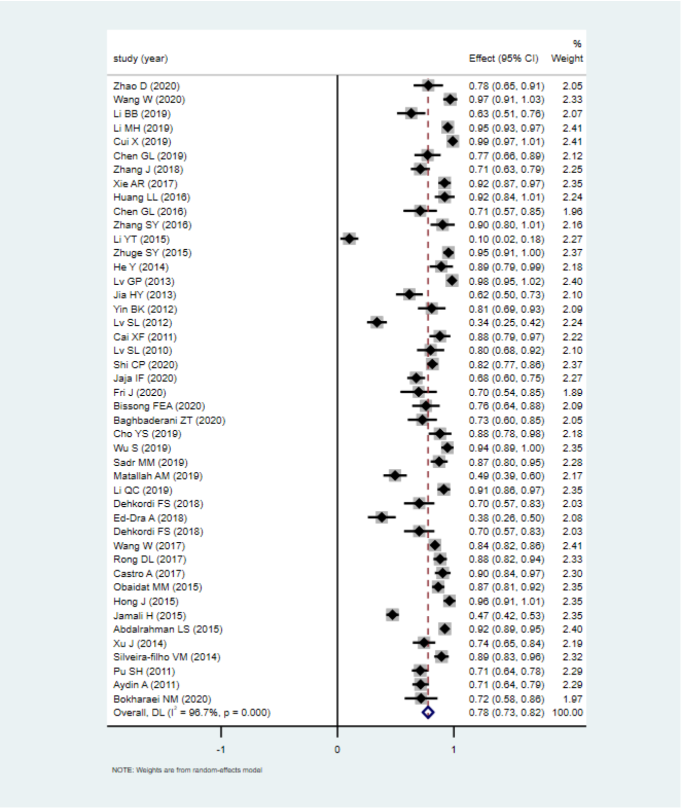

Figure 8. β-Lactams resistance in *Staphylococcus aureus* isolates from food

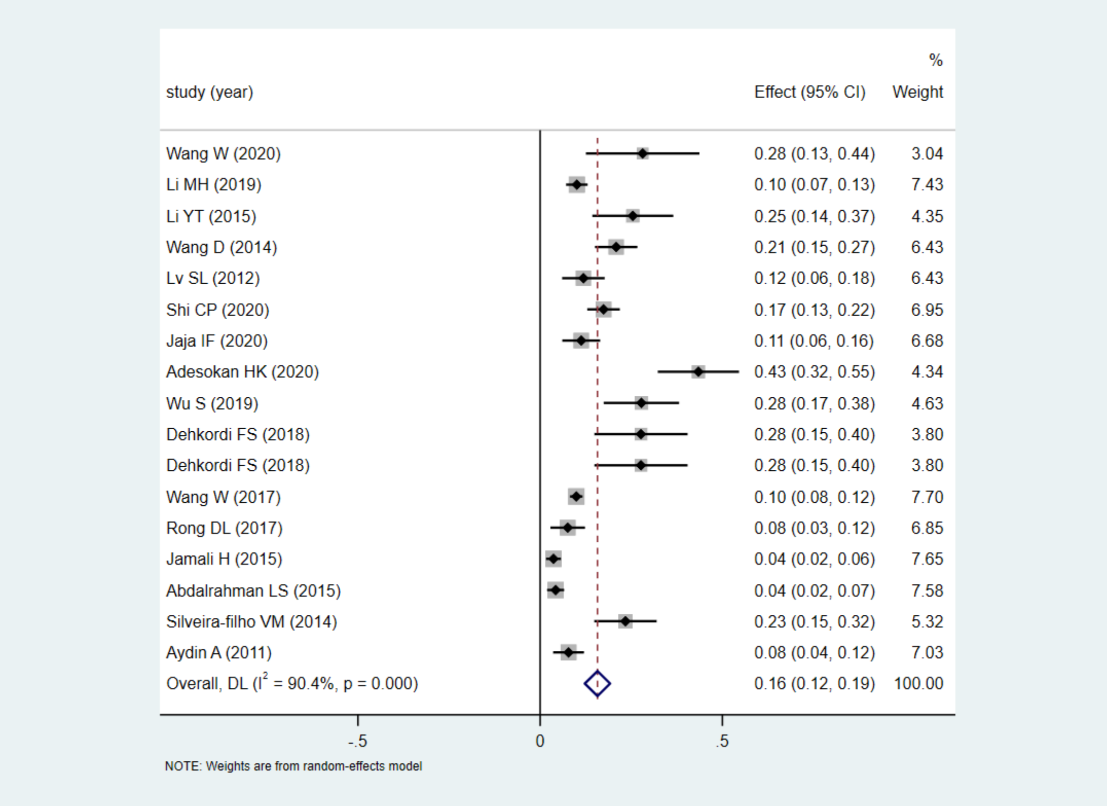

Figure 9. Chloramphenicol resistance in *Staphylococcus aureus* isolates from food

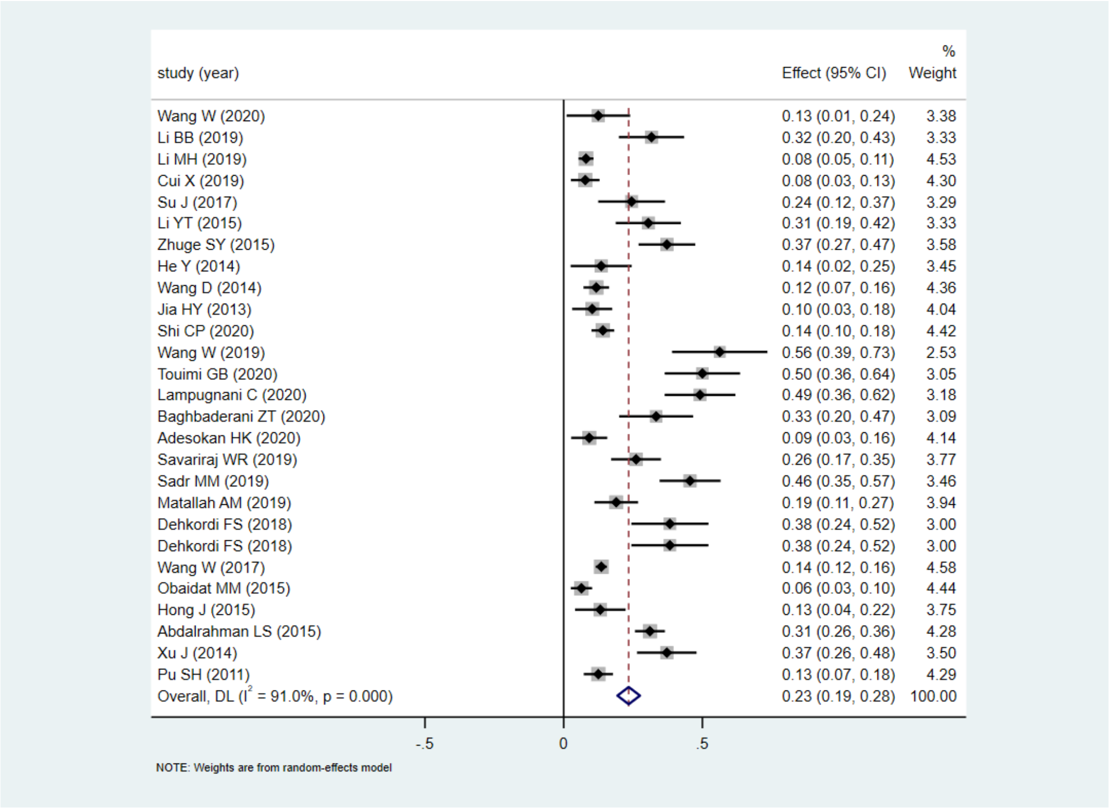

Figure 10. Fluoroquinolones resistance in *Staphylococcus aureus* isolates from food

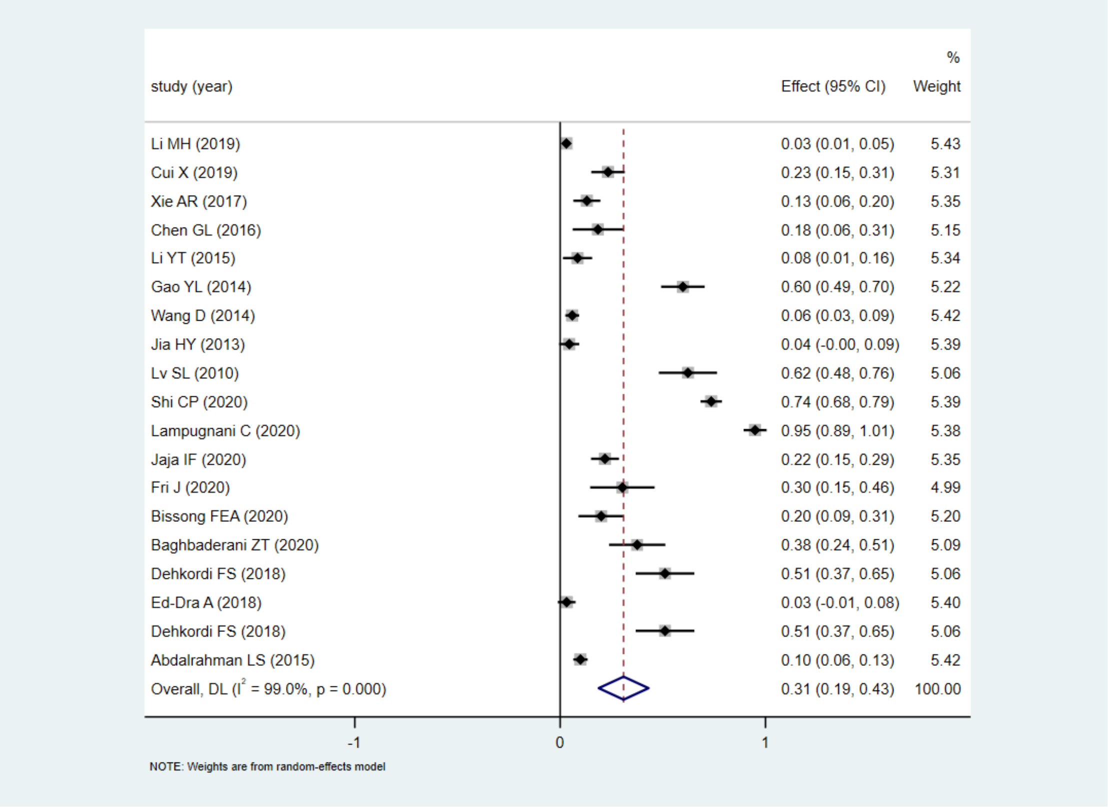

Figure 11. Sulfonamides resistance in *Staphylococcus aureus* isolates from food

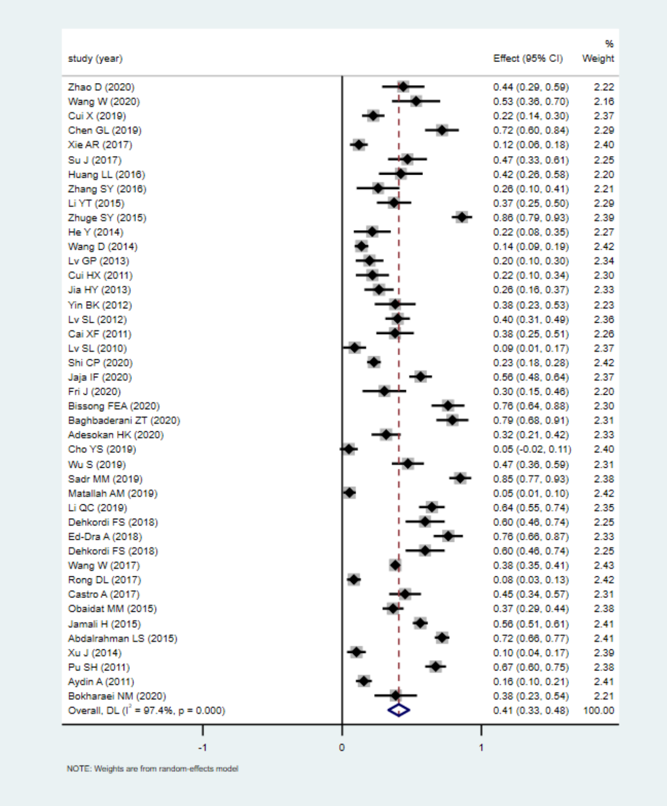

Figure 12. Tetracyclines resistance in *Staphylococcus aureus* isolates from food

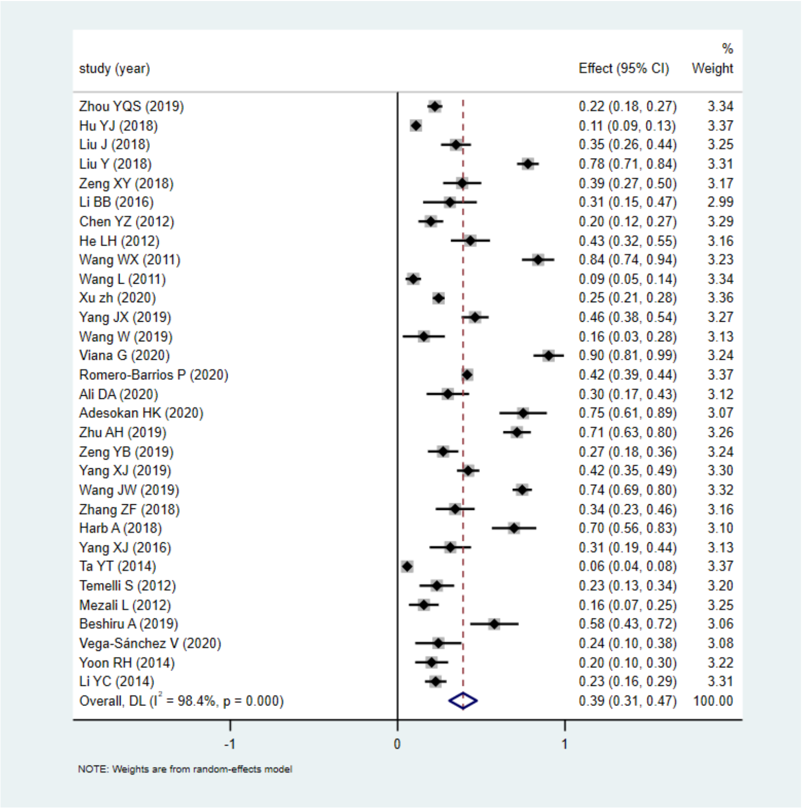

Figure 13. Aminoglycosides resistance in *Salmonella* isolates from food

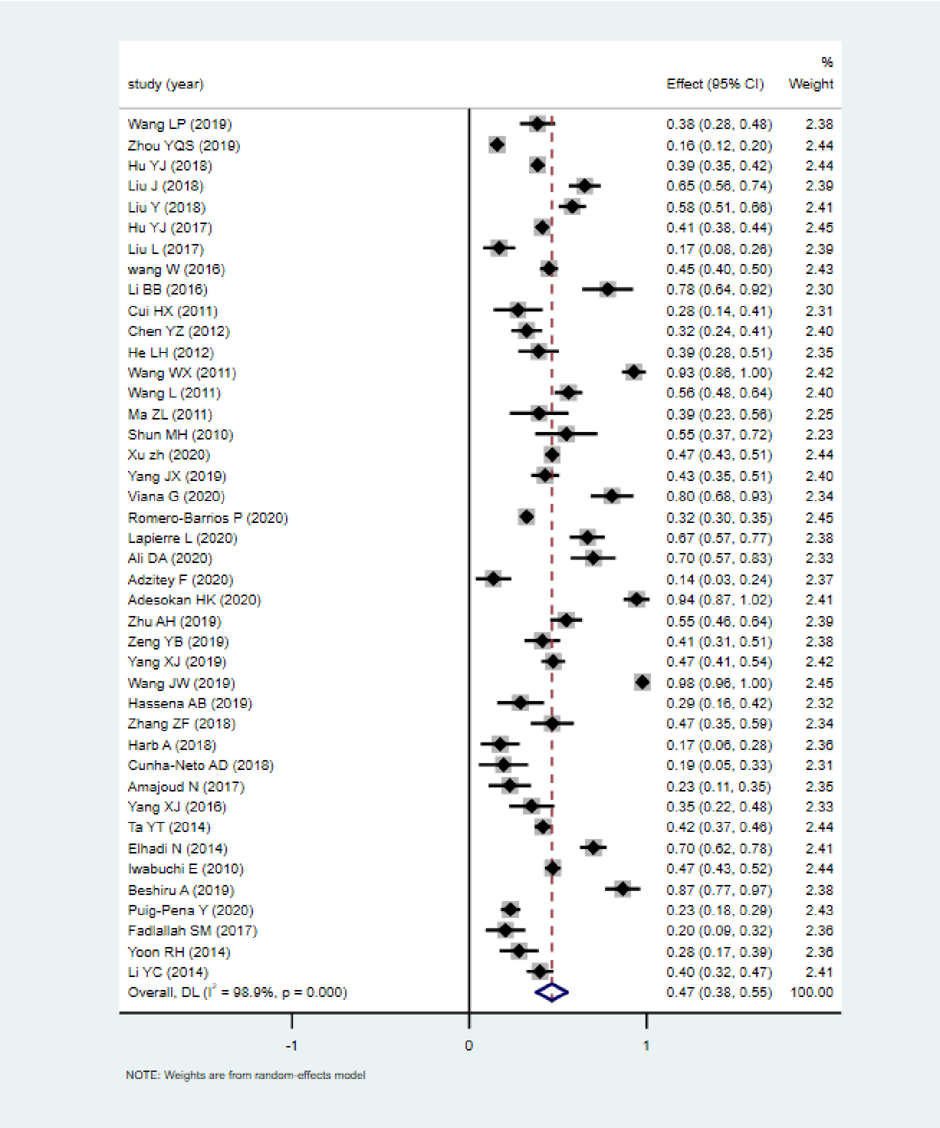

Figure 14. β-Lactams resistance in *Salmonella* isolates from food

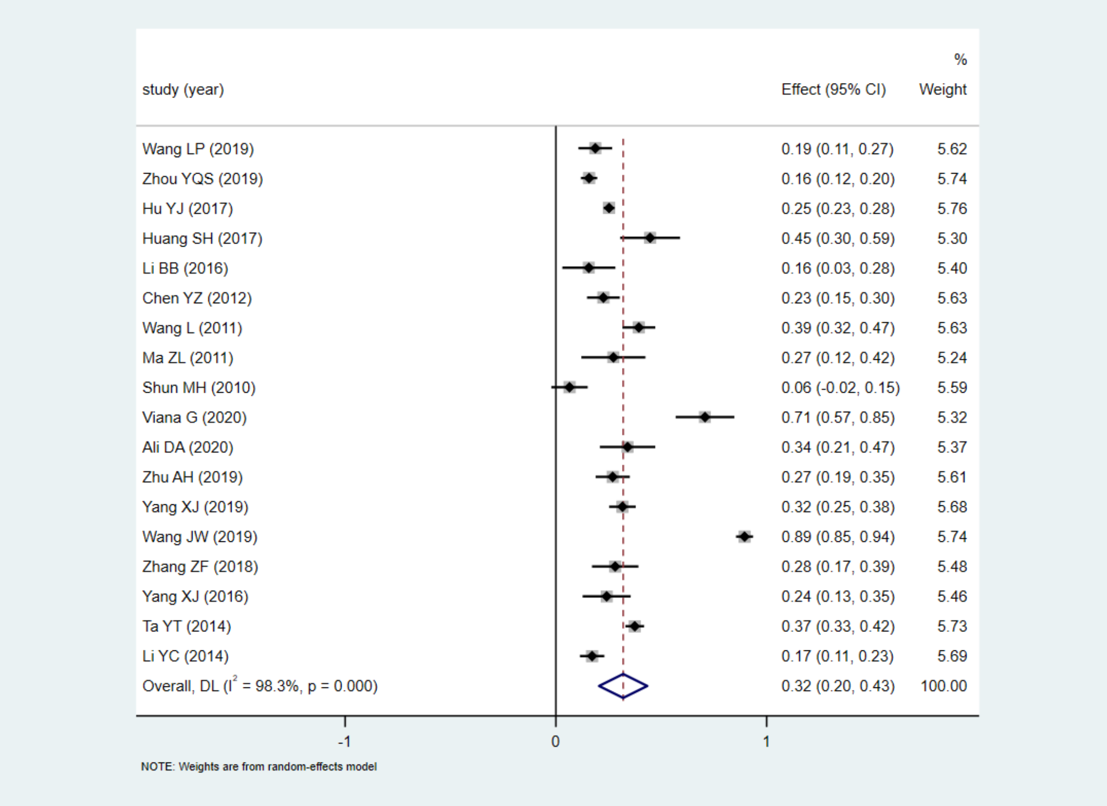

Figure 15. Chloramphenicol resistance in *Salmonella* isolates from food

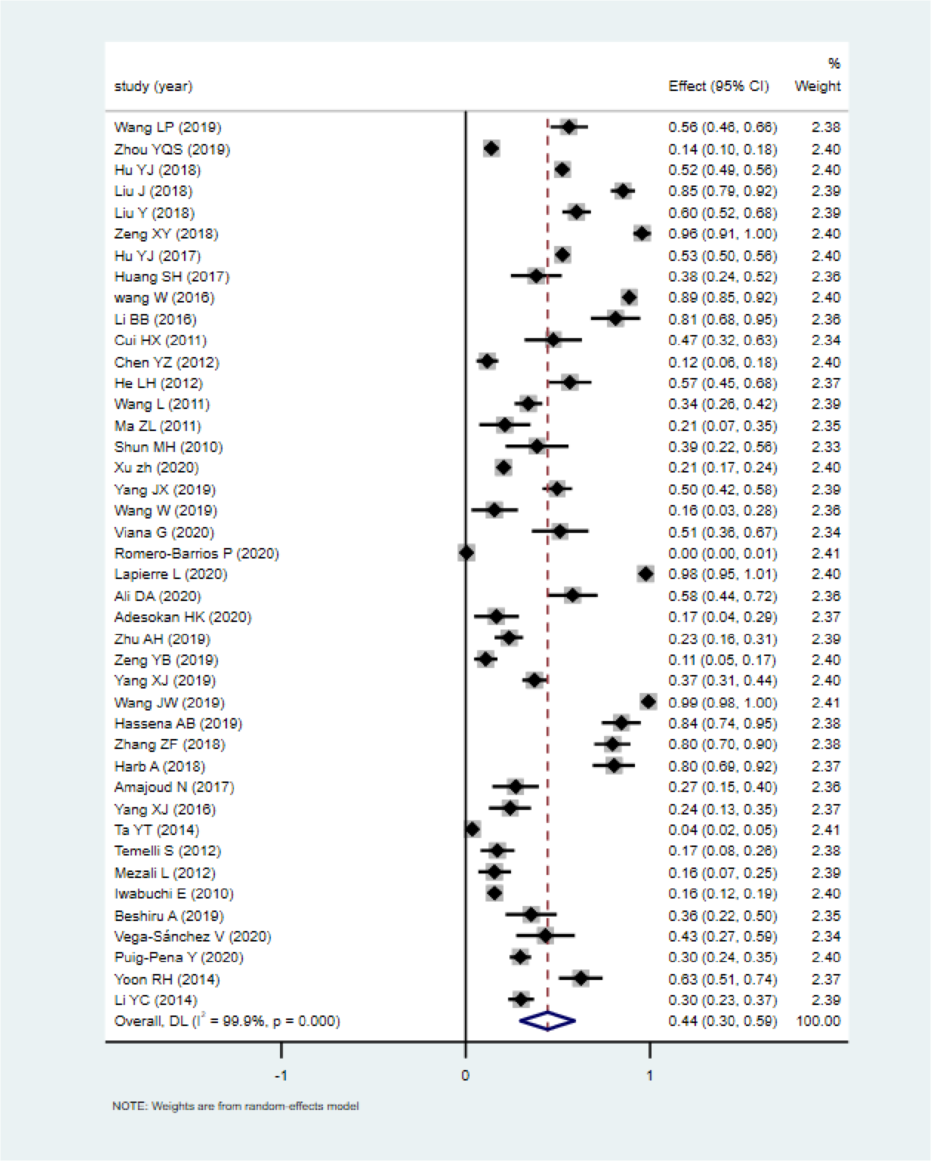

Figure 16. Fluoroquinolones resistance in *Salmonella* isolates from food

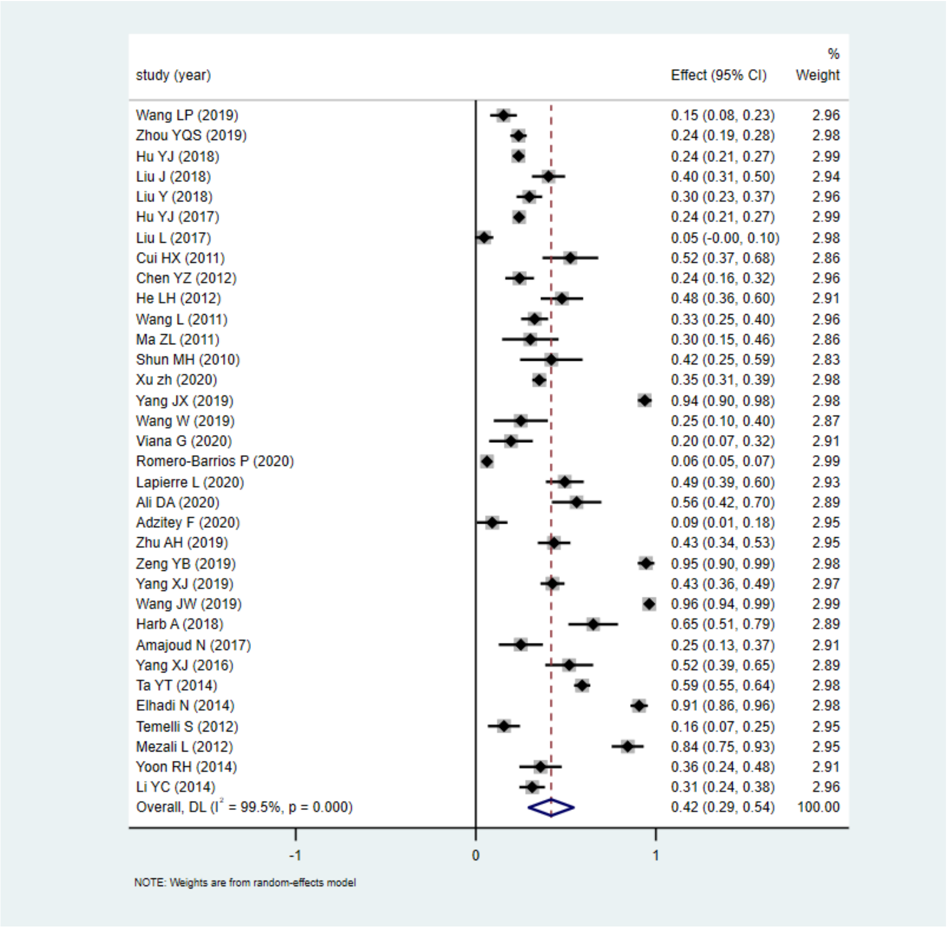

Figure 17. Sulfonamides resistance in *Salmonella* isolates from food

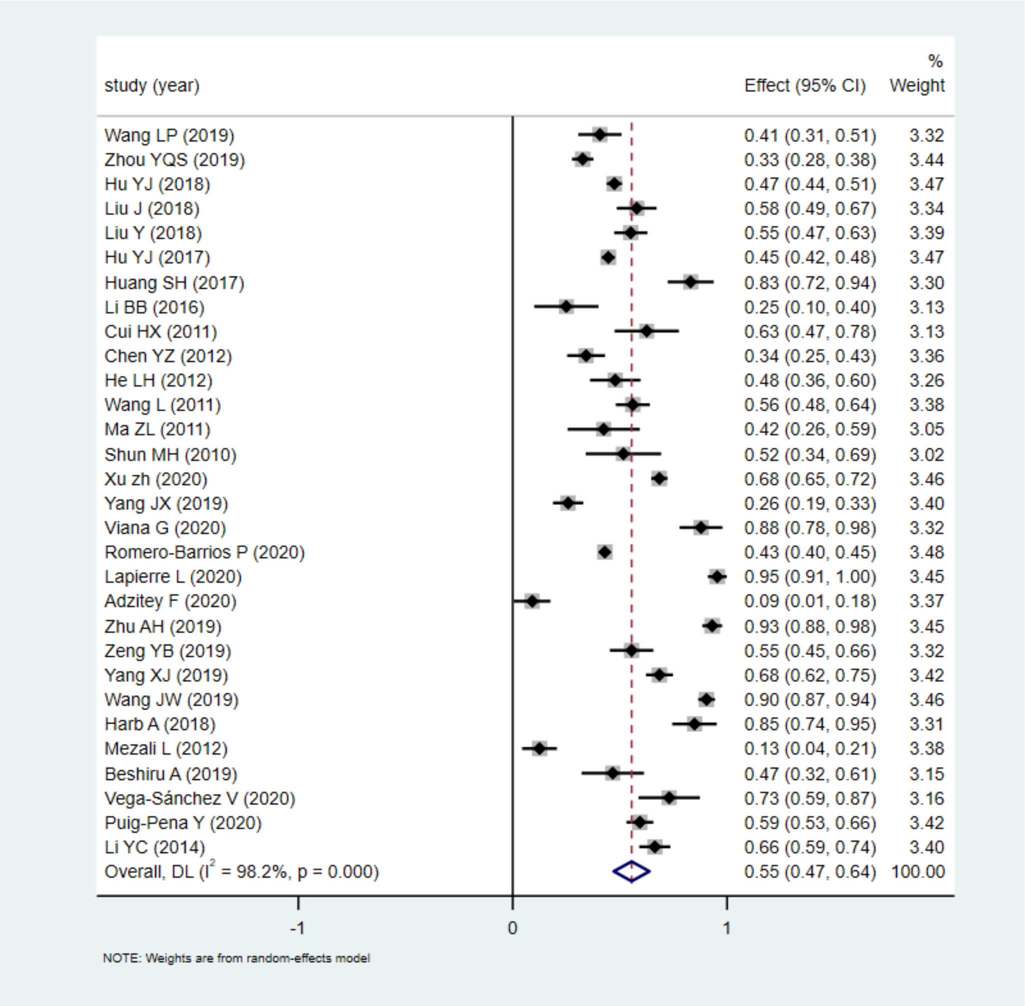

Figure 18. Tetracyclines resistance in *Salmonella* isolates from food

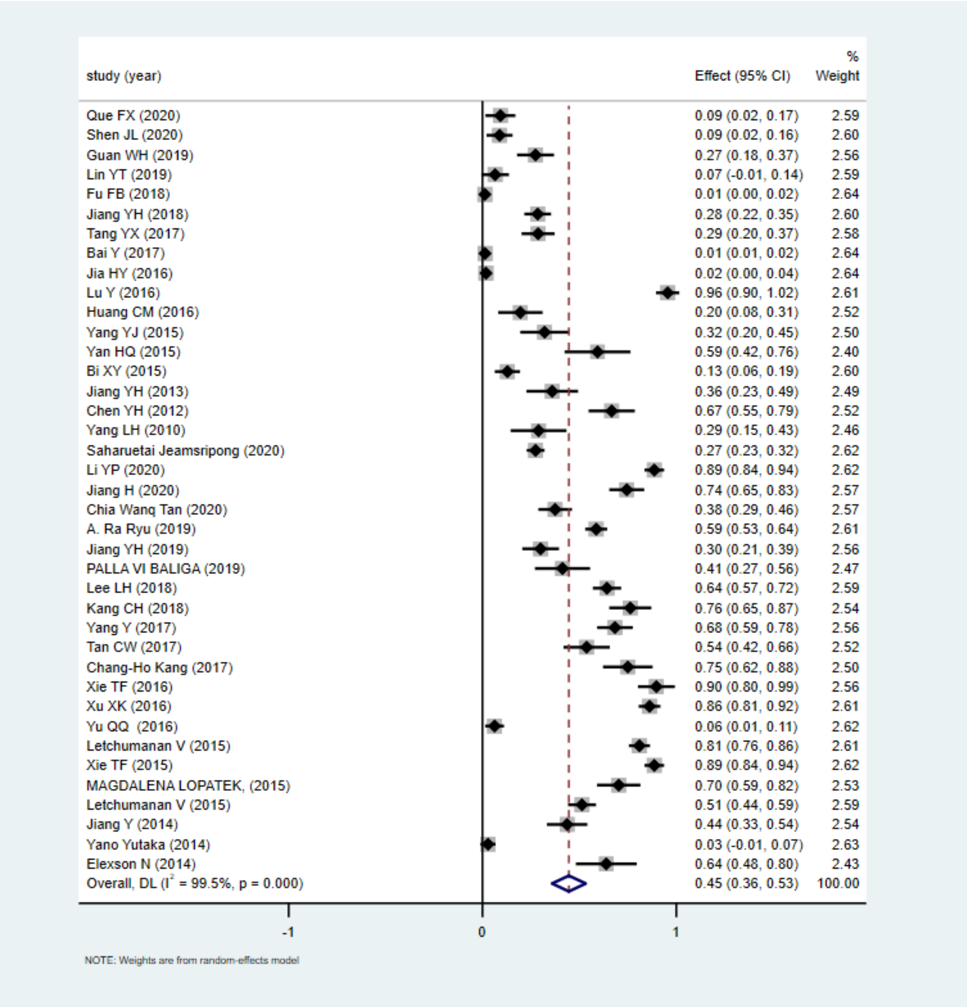

Figure 19. Aminoglycosides resistance in *Vibrio parahaemolyticus* isolates from food

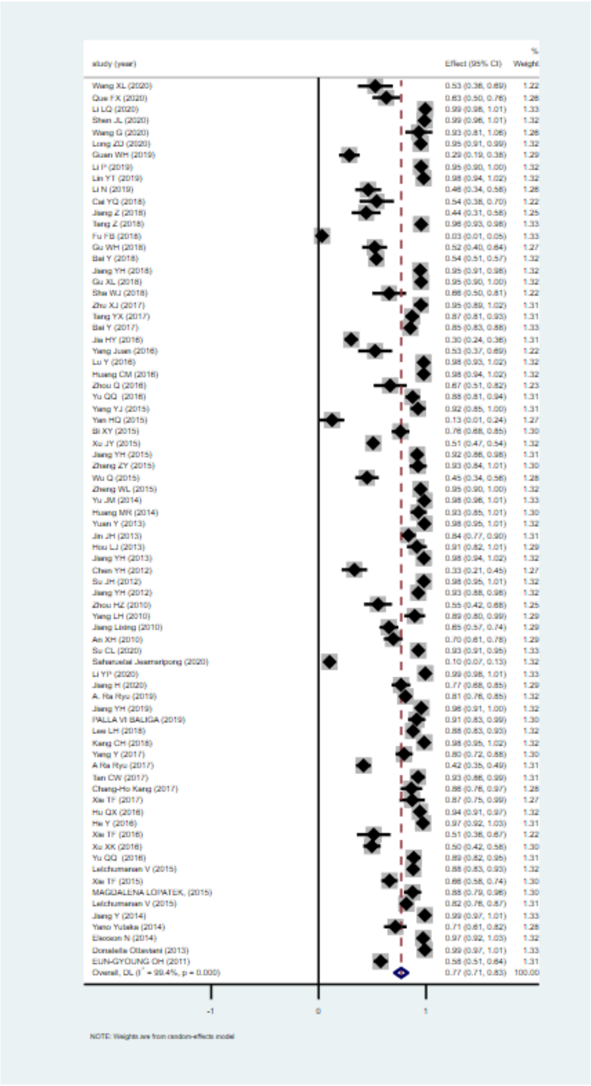

Figure 20. β-Lactams resistance in *Vibrio parahaemolyticus* isolates from food

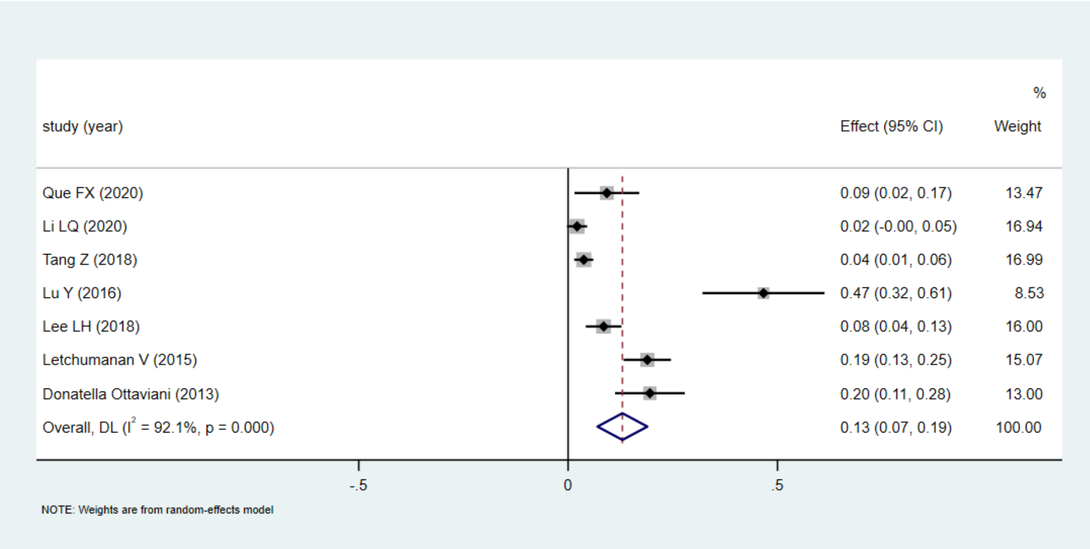

Figure 21. Fluoroquinolones resistance in *Vibrio parahaemolyticus* isolates from food

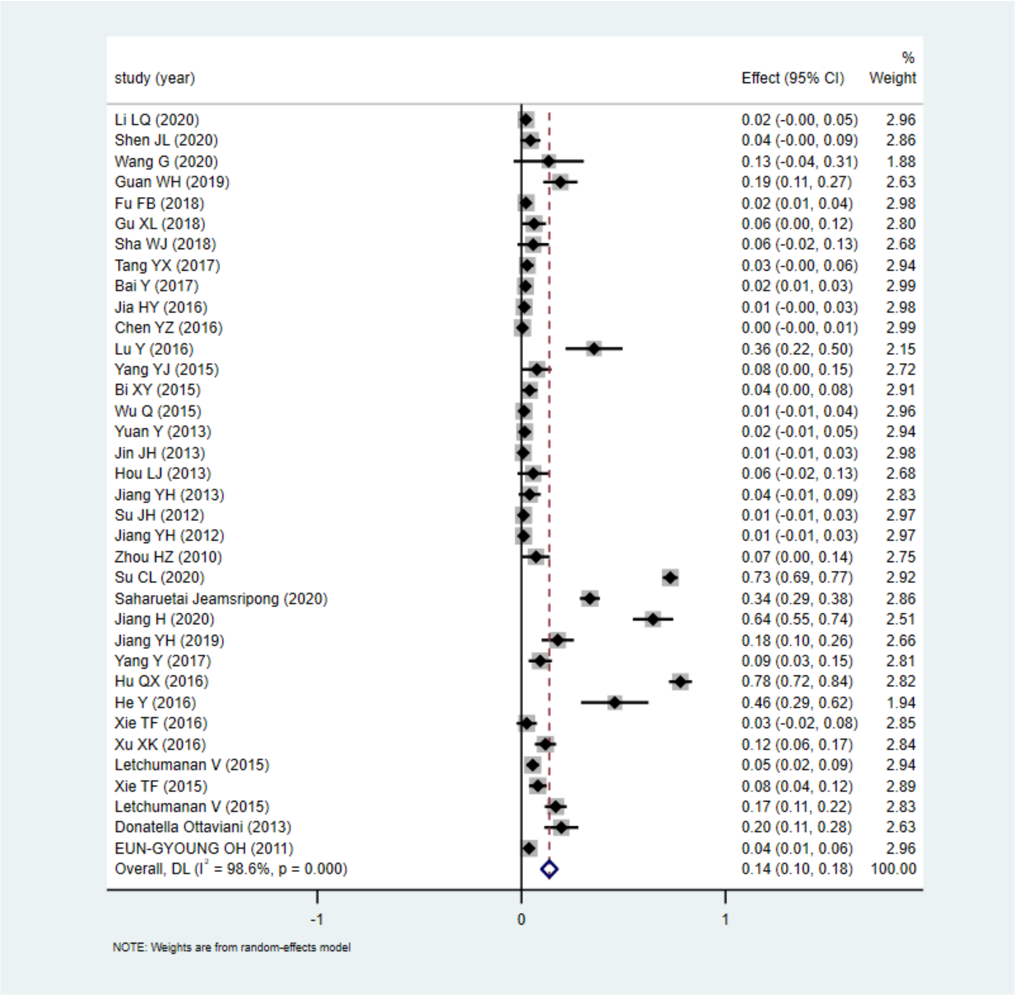

Figure 22. Sulfonamides resistance in *Vibrio parahaemolyticus* isolates from food

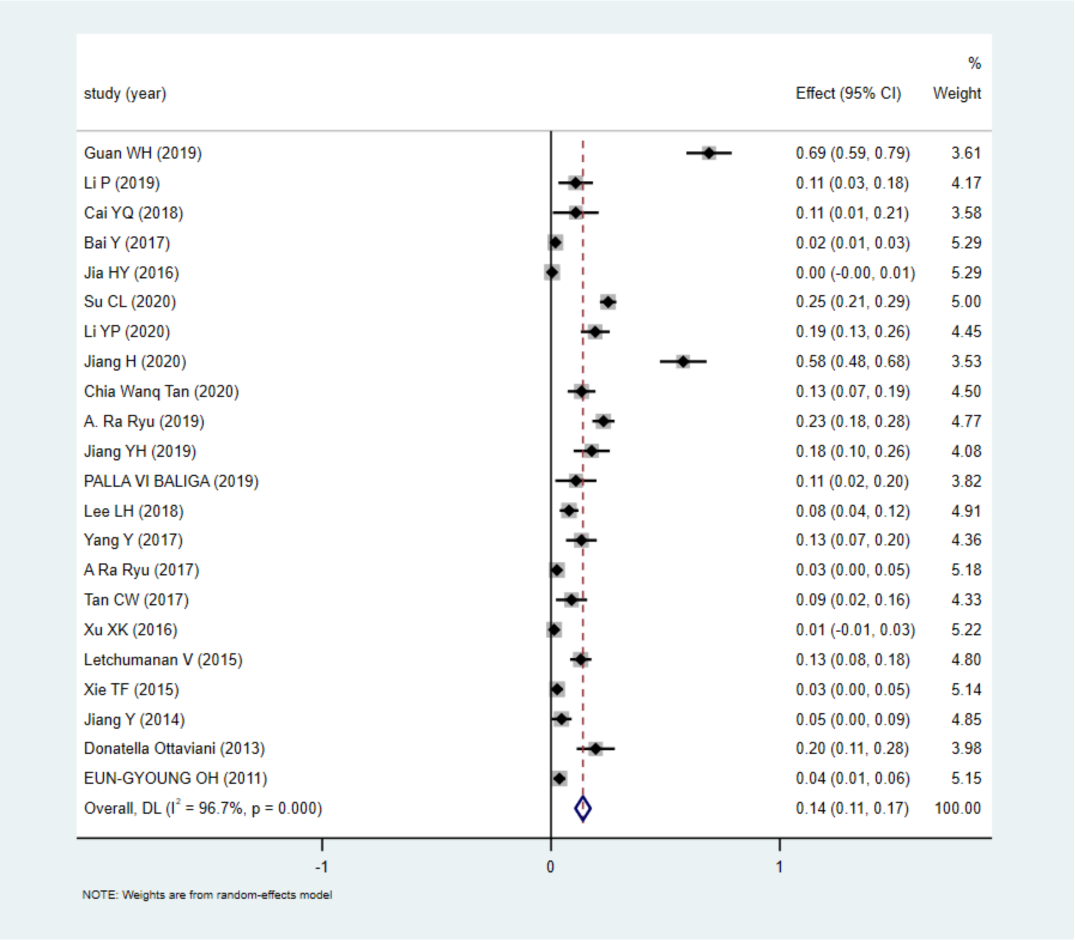

Figure 23. Tetracyclines resistance in *Vibrio parahaemolyticus* isolates from food

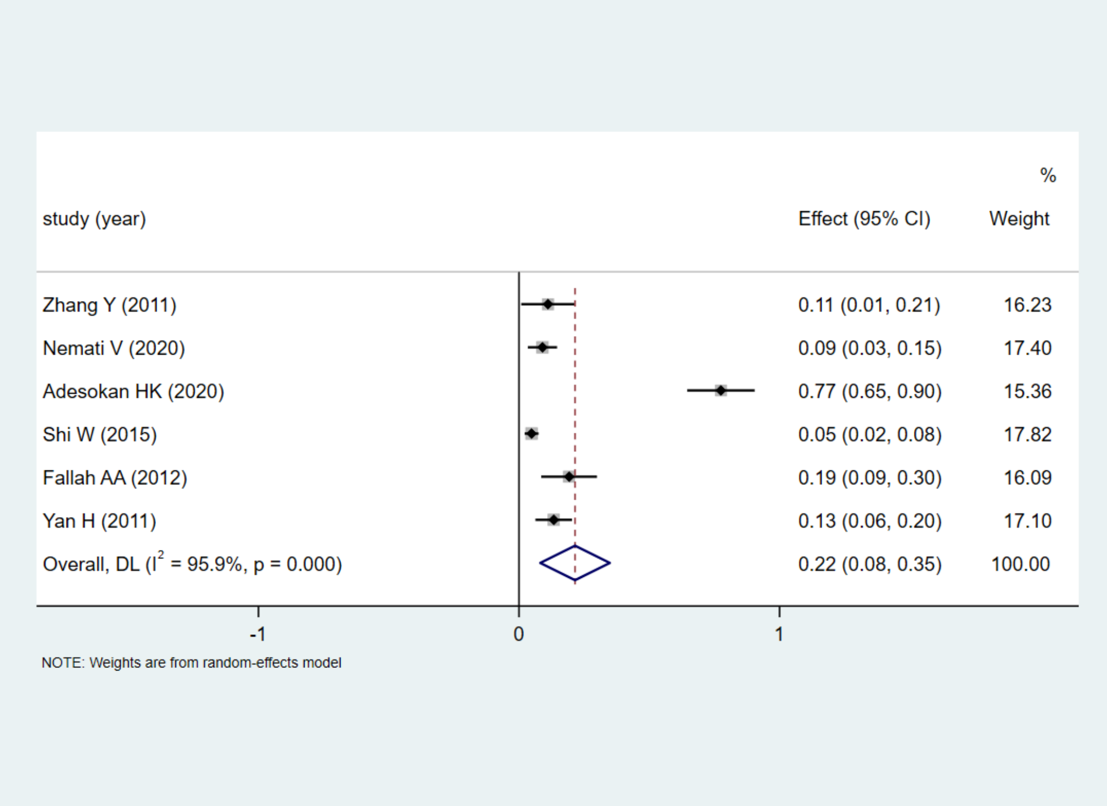

Figure 24. Aminoglycosides resistance in *Listeria monocytogenes* isolates from food

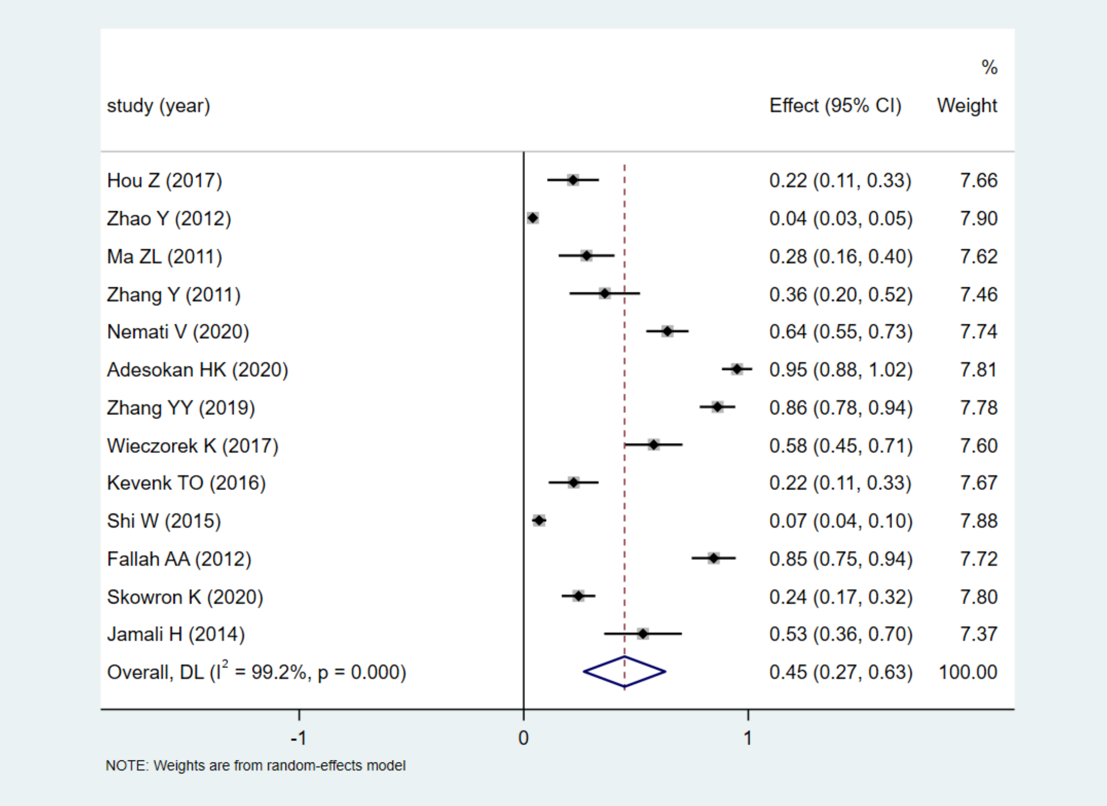

Figure 25. β-Lactams resistance in *Listeria monocytogenes* isolates from food

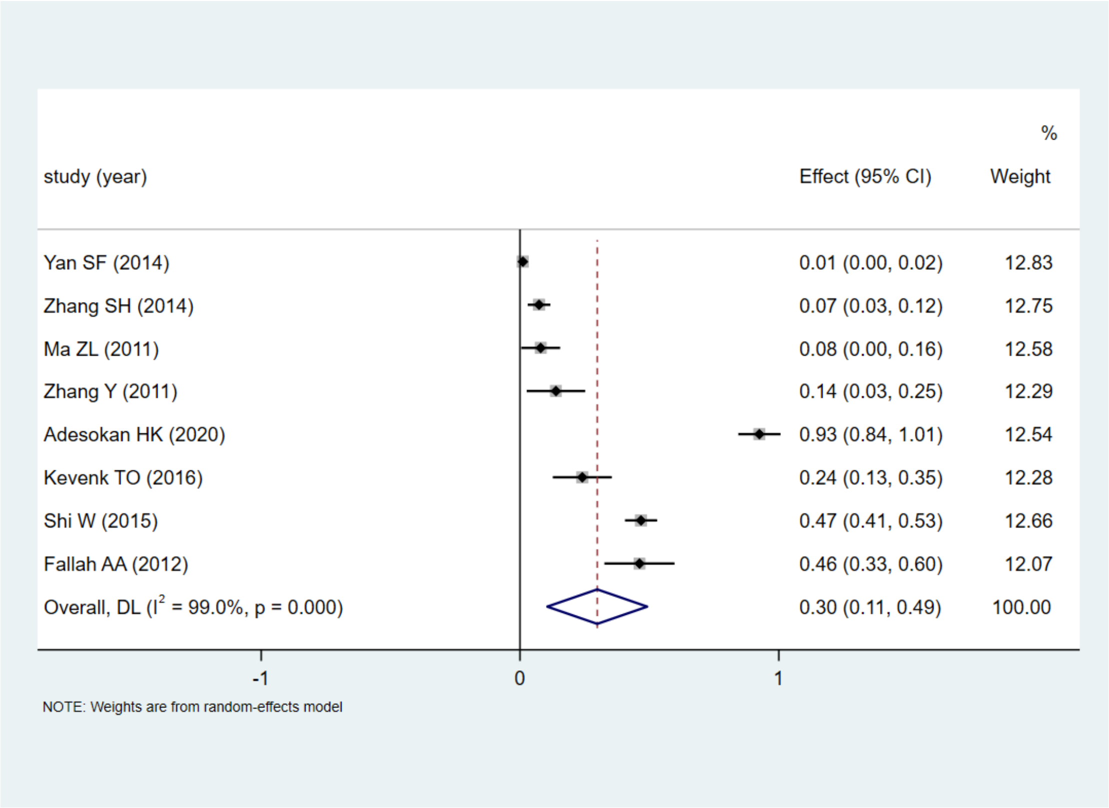

Figure 26. Chloramphenicol resistance in *Listeria monocytogenes* isolates from food

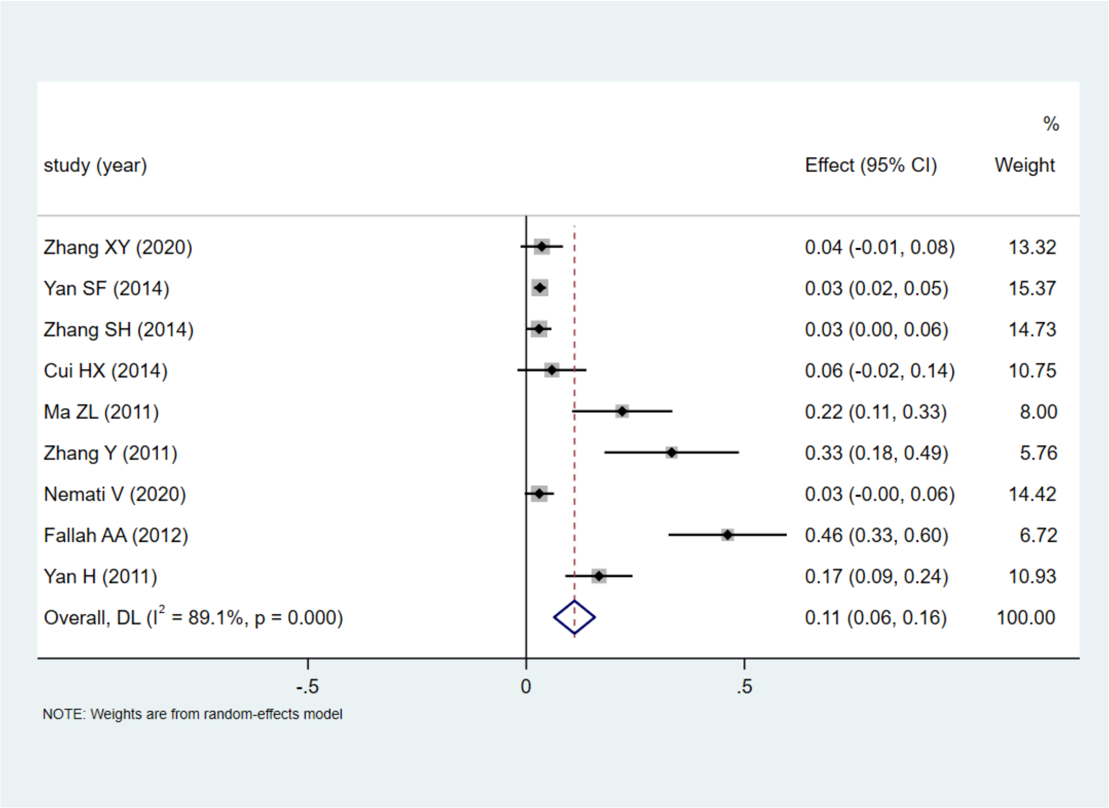

Figure 27. Fluoroquinolones resistance in *Listeria monocytogenes* isolates from food

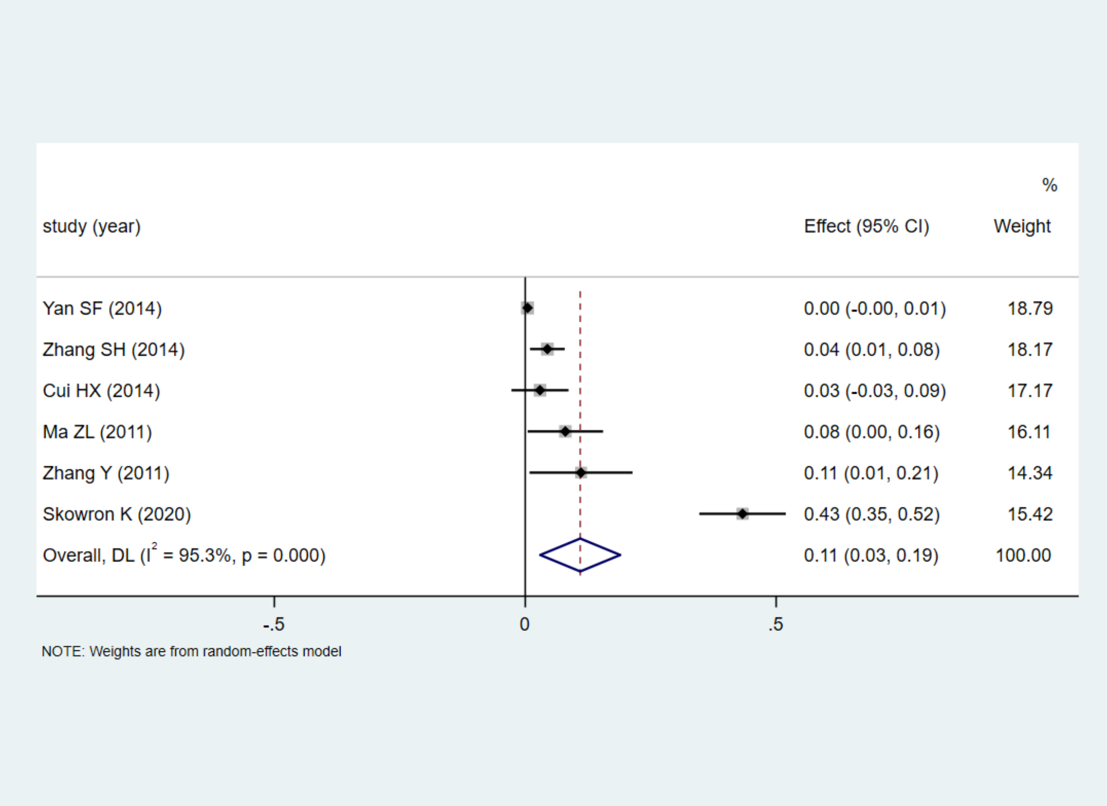

Figure 28. Sulfonamides resistance in *Listeria monocytogenes* isolates from food

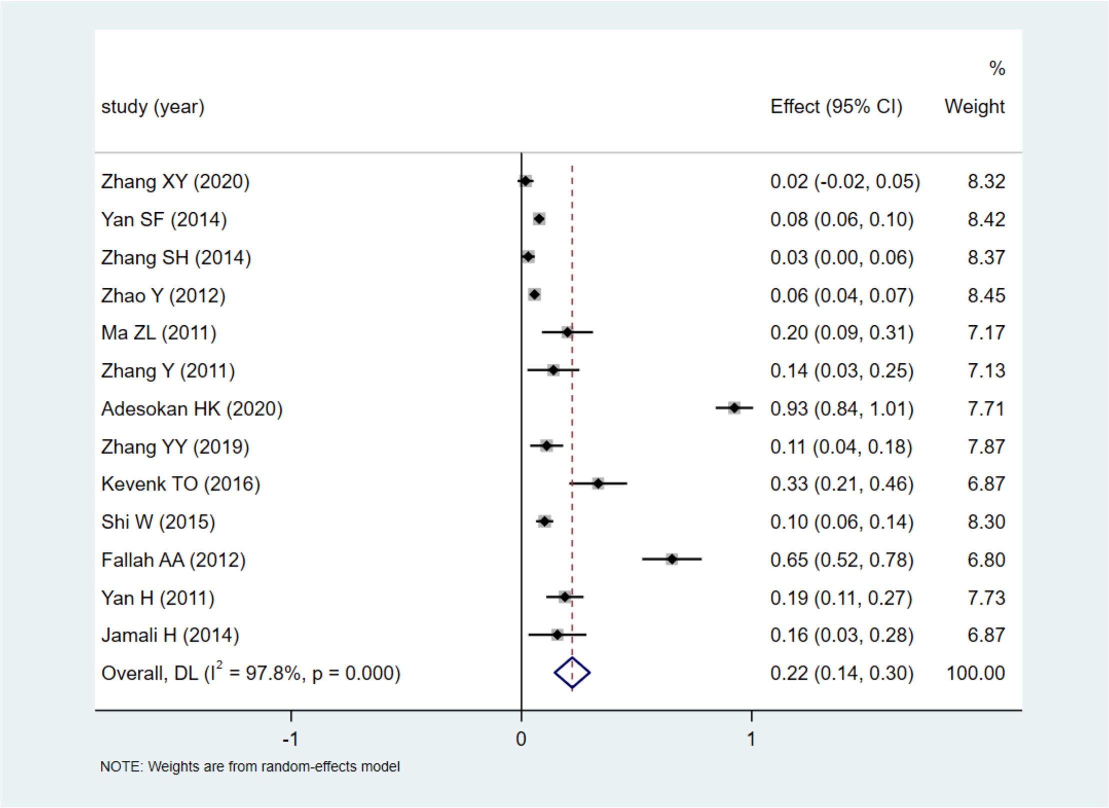

Figure 29. Tetracyclines resistance in *Listeria monocytogenes* isolates from food

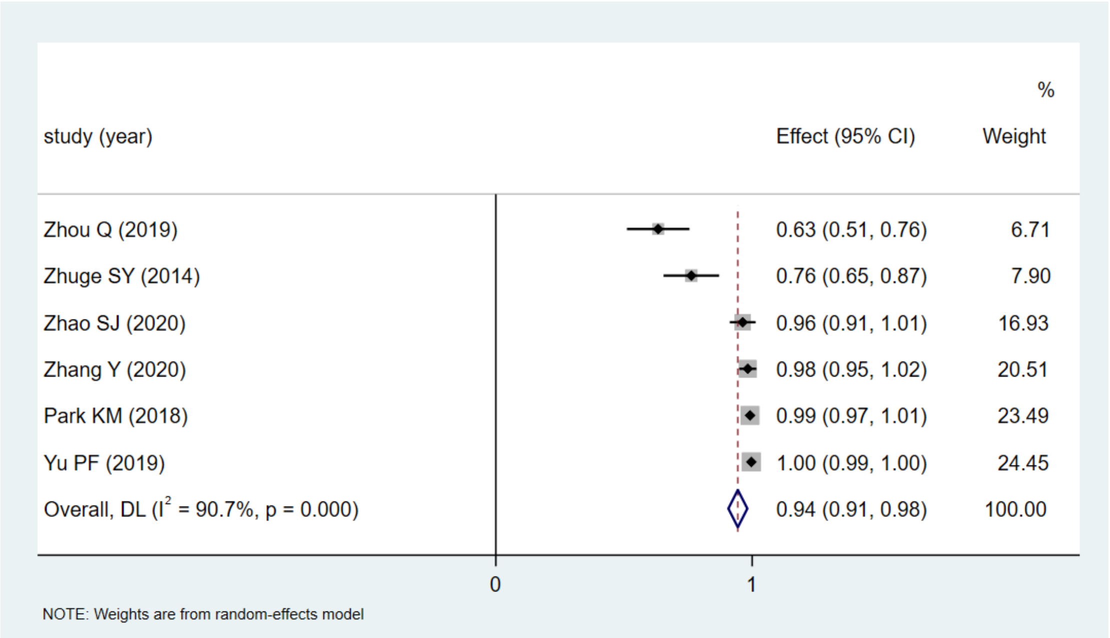

Figure 30. β-Lactams resistance in *Bacillus cereus* isolates from food

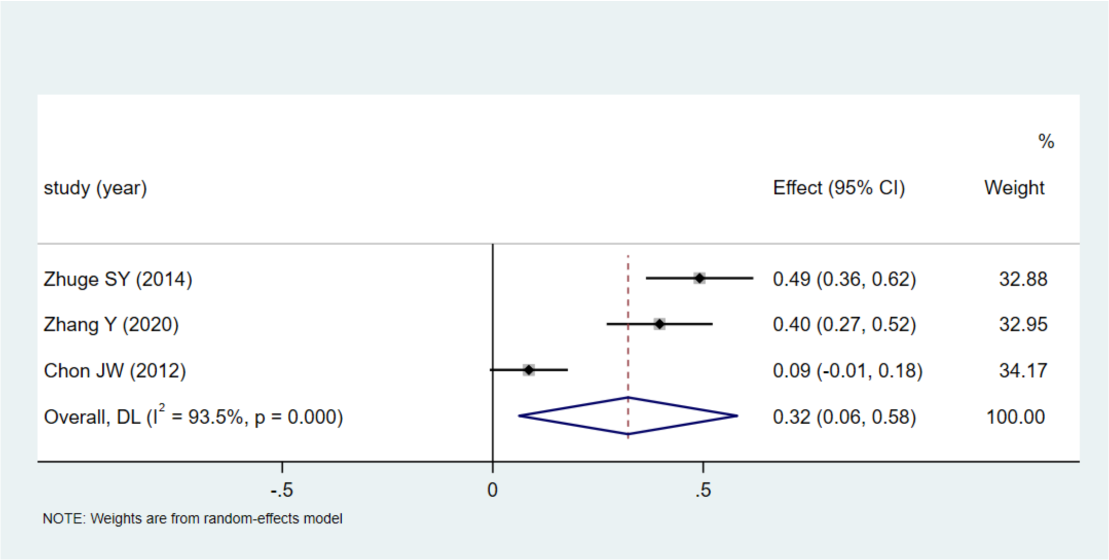

Figure 31. Sulfonamides resistance in *Bacillus cereus* isolates from food

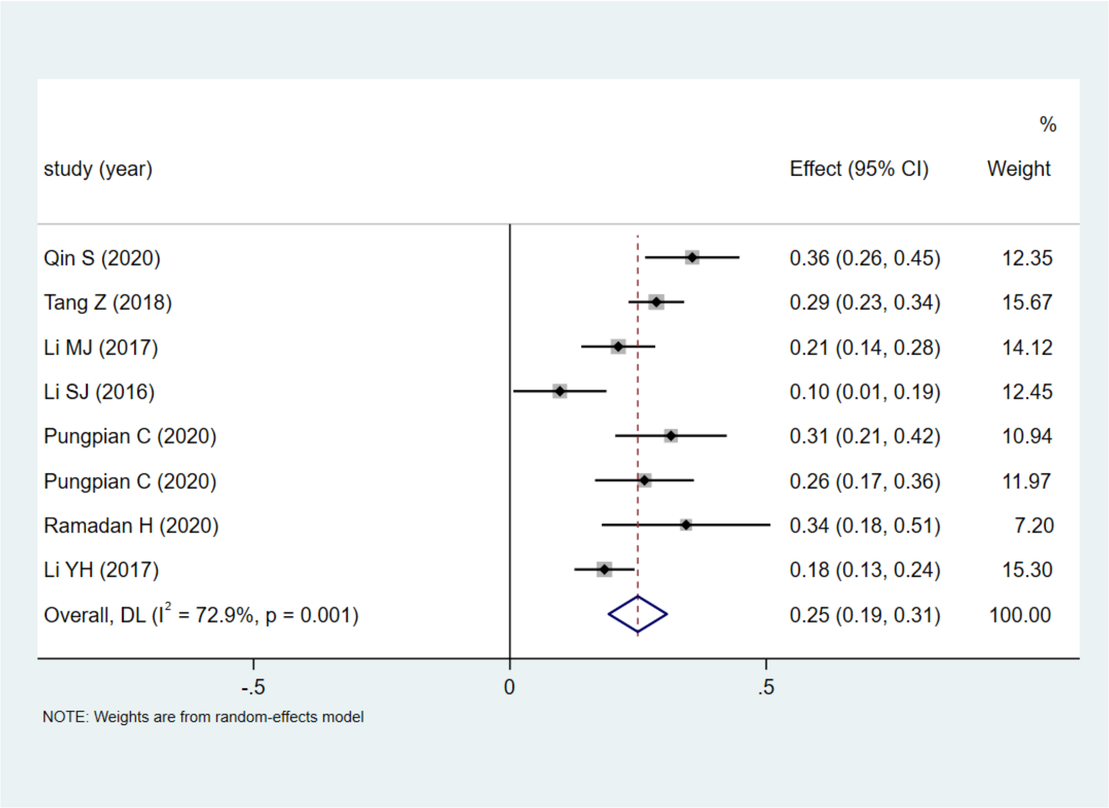

Figure 32. Aminoglycosides resistance in *Escherichia coli* isolates from human

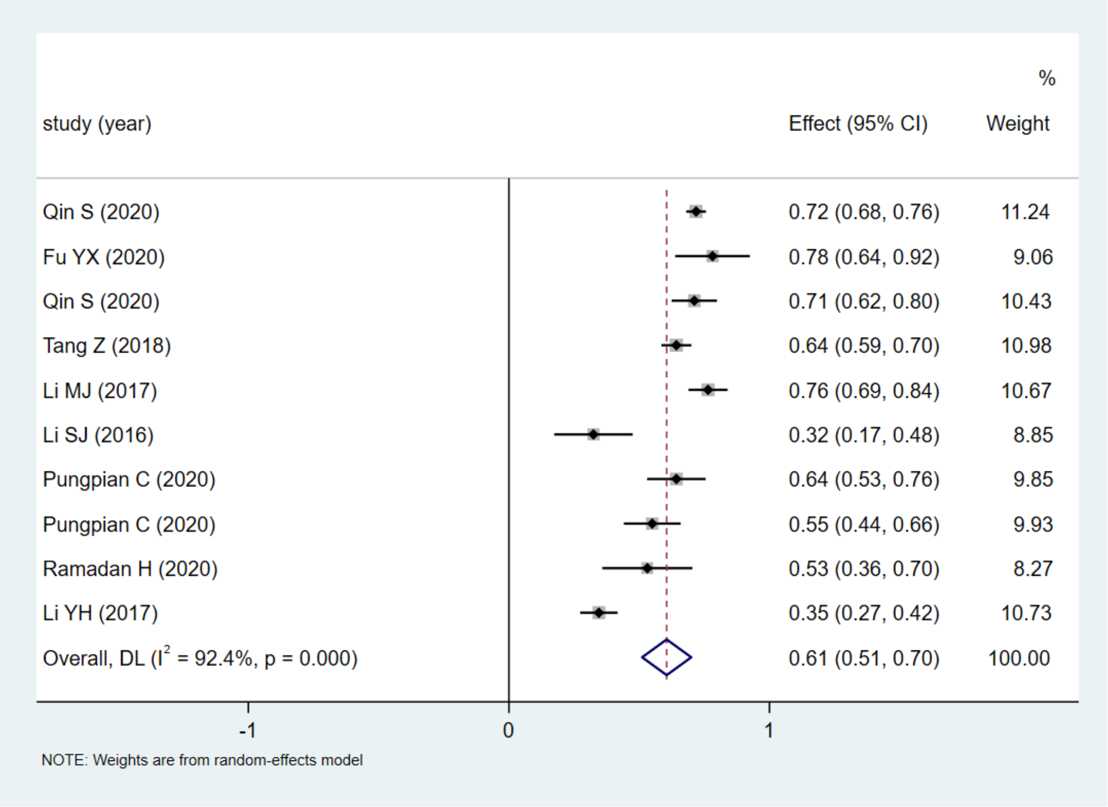

Figure 33. β-Lactams resistance in *Escherichia coli* isolates from human

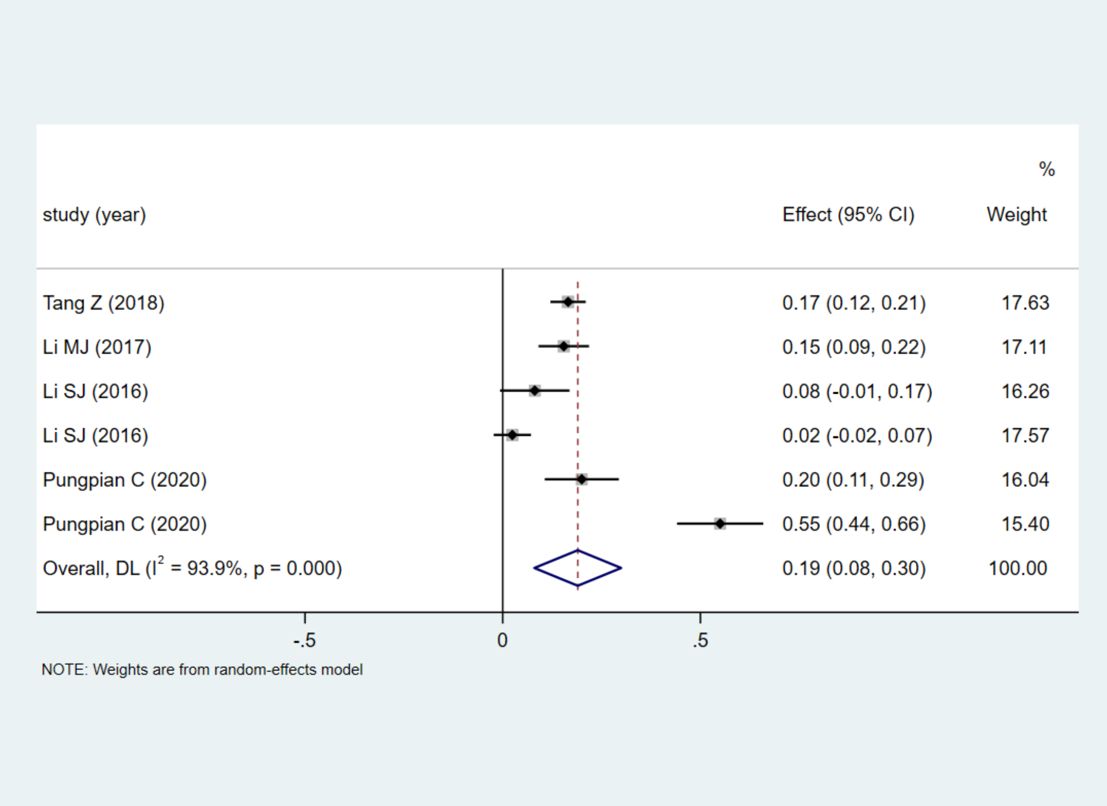

Figure 34. Chloramphenicol resistance in *Escherichia coli* isolates from human

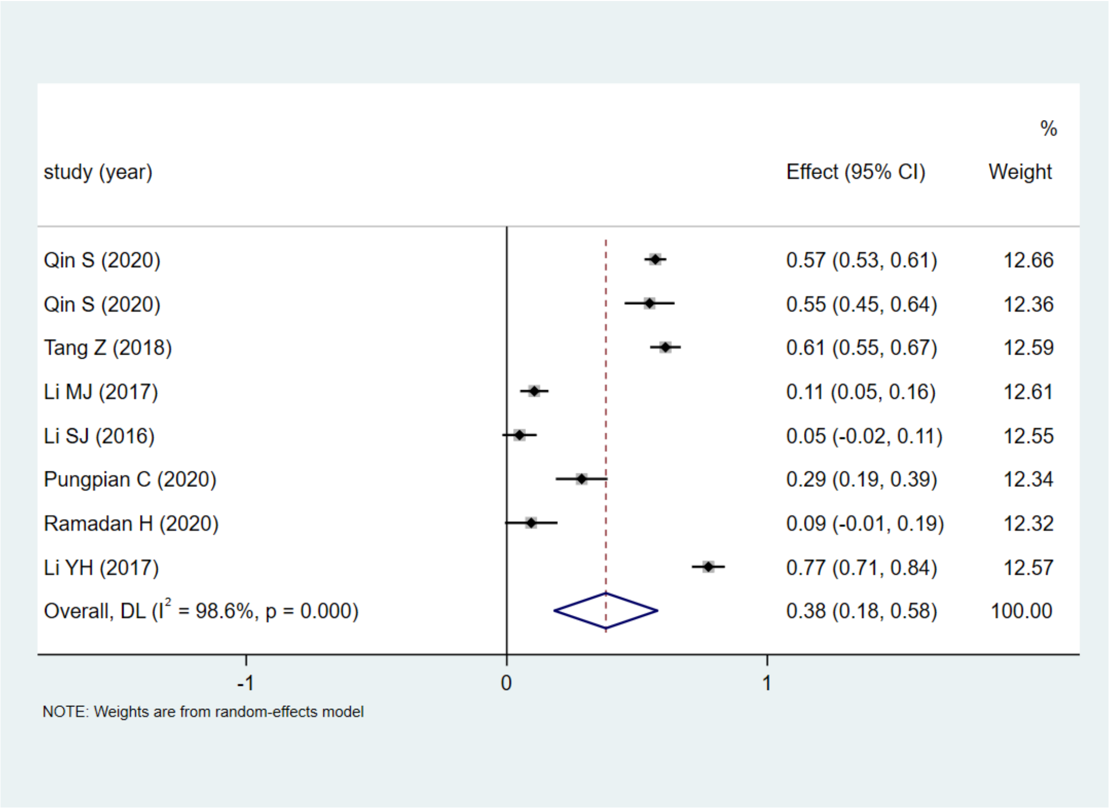

Figure 35. Fluoroquinolones resistance in *Escherichia coli* isolates from human

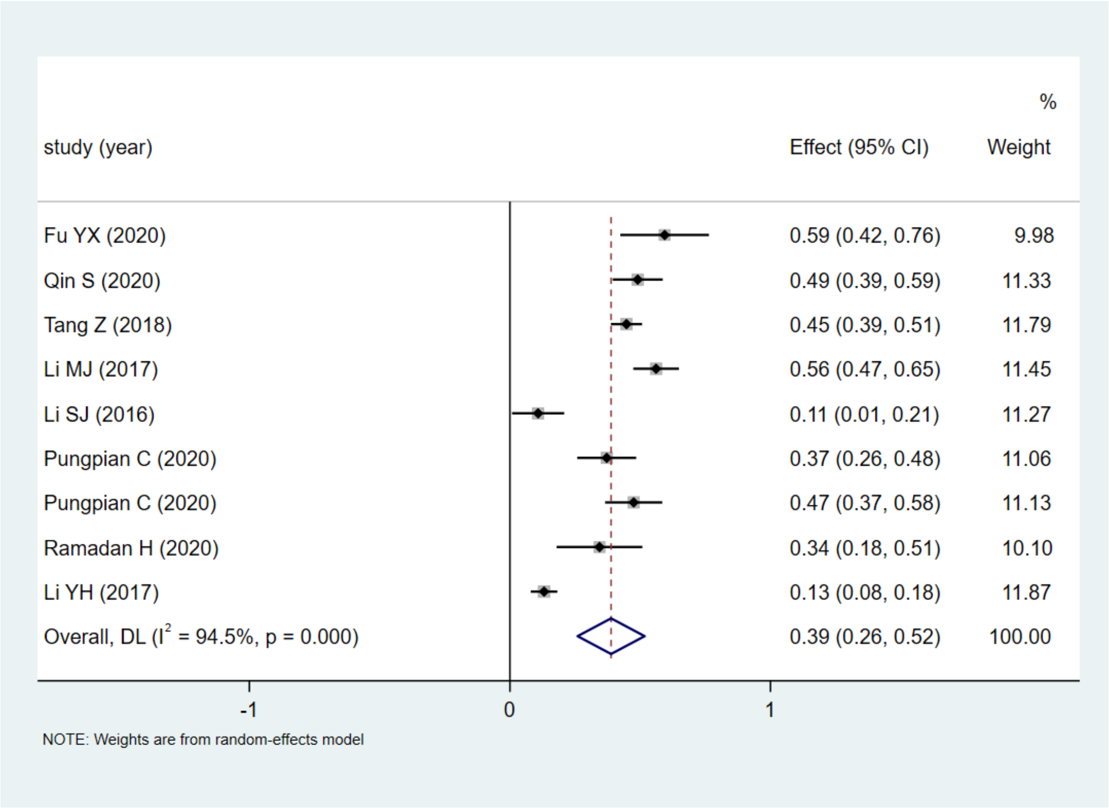

Figure 36. Sulfonamides resistance in *Escherichia coli* isolates from human

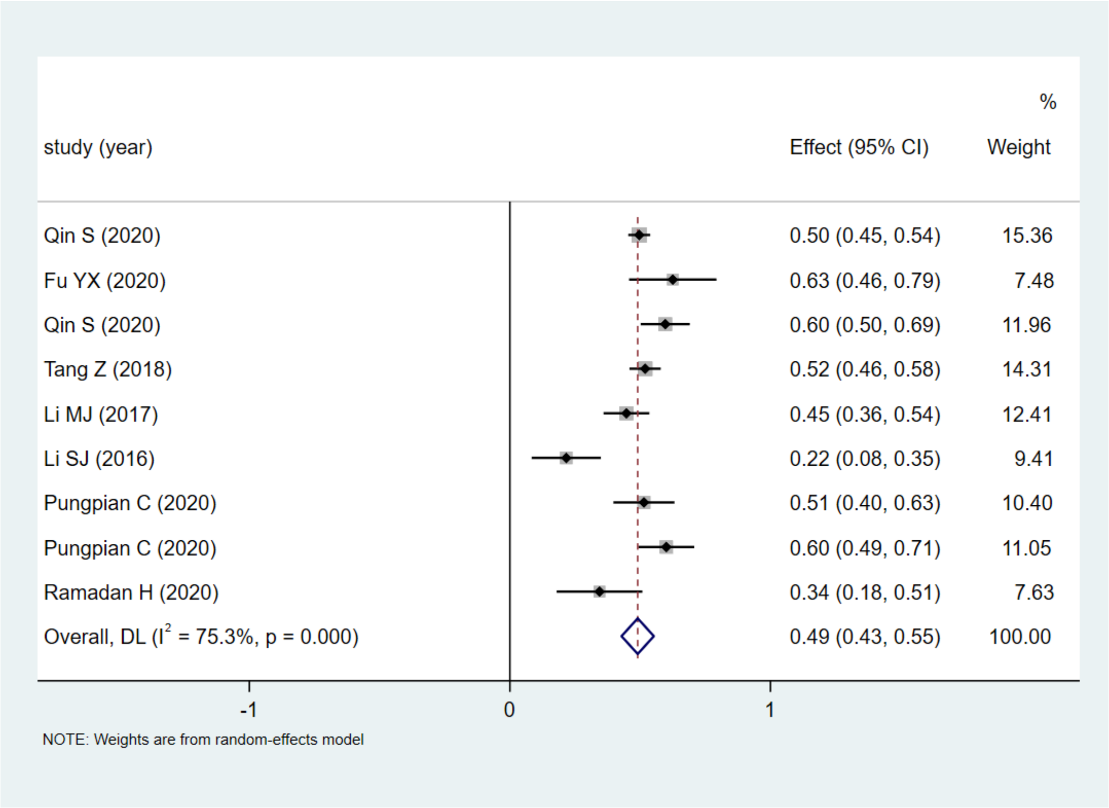

Figure 37. Tetracyclines resistance in *Escherichia coli* isolates from human

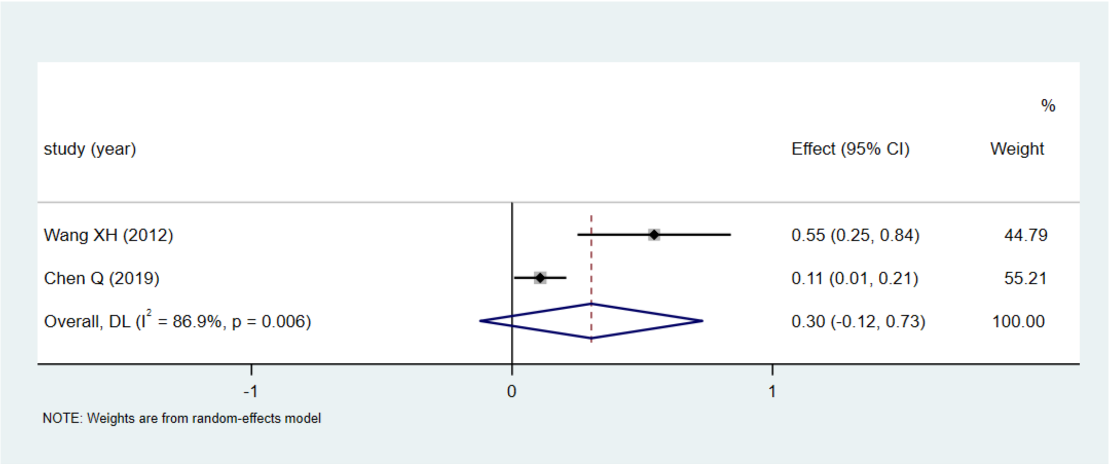

Figure 38. Aminoglycosides resistance in *Staphylococcus aureus* isolates from human

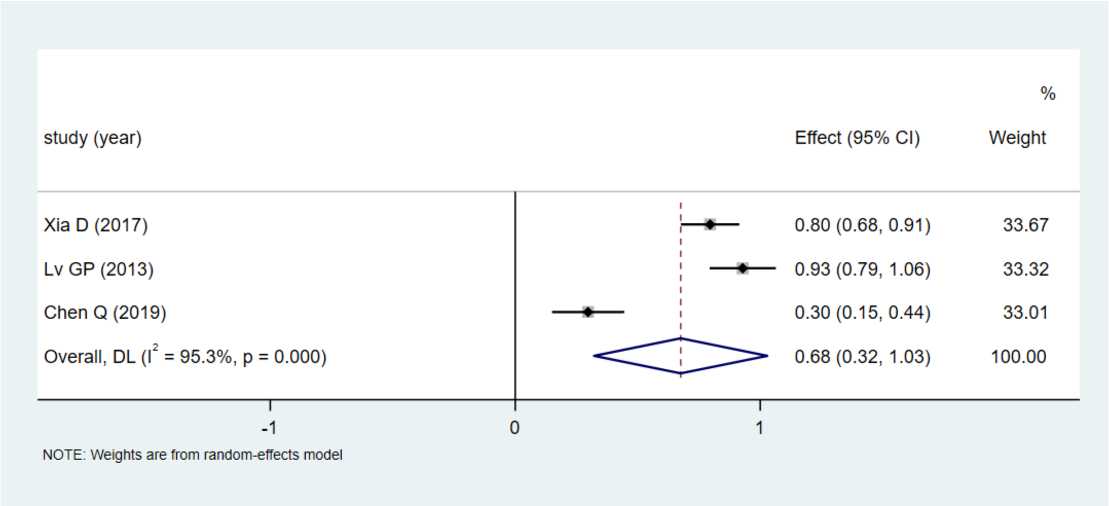

Figure 39. β-Lactams resistance in *Staphylococcus aureus* isolates from human

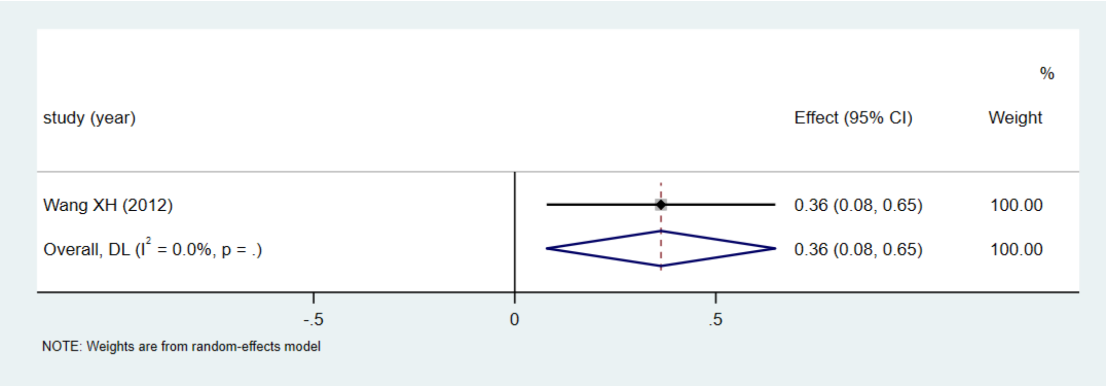

Figure 40. Fluoroquinolones resistance in *Staphylococcus aureus* isolates from human

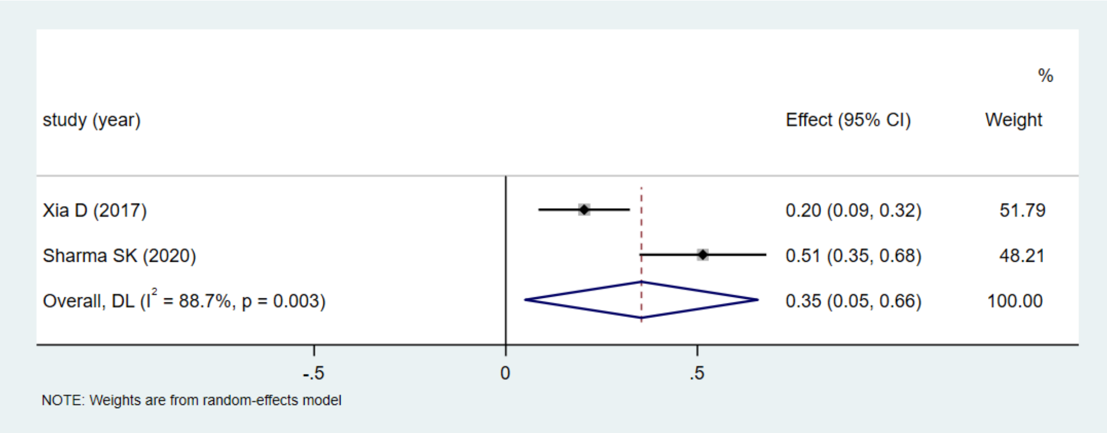

Figure 41. Sulfonamides resistance in *Staphylococcus aureus* isolates from human

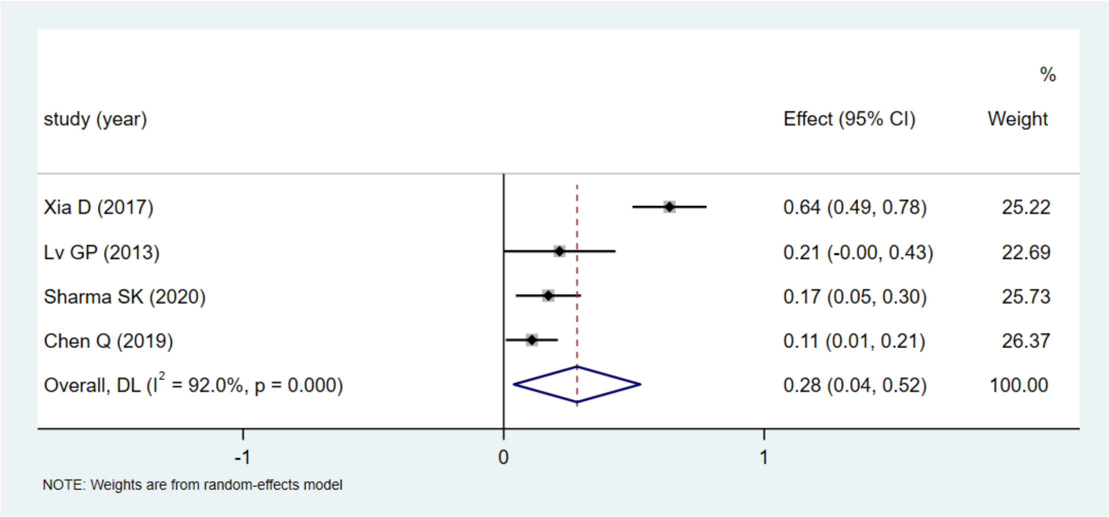

Figure 42. Tetracyclines resistance in *Staphylococcus aureus* isolates from human

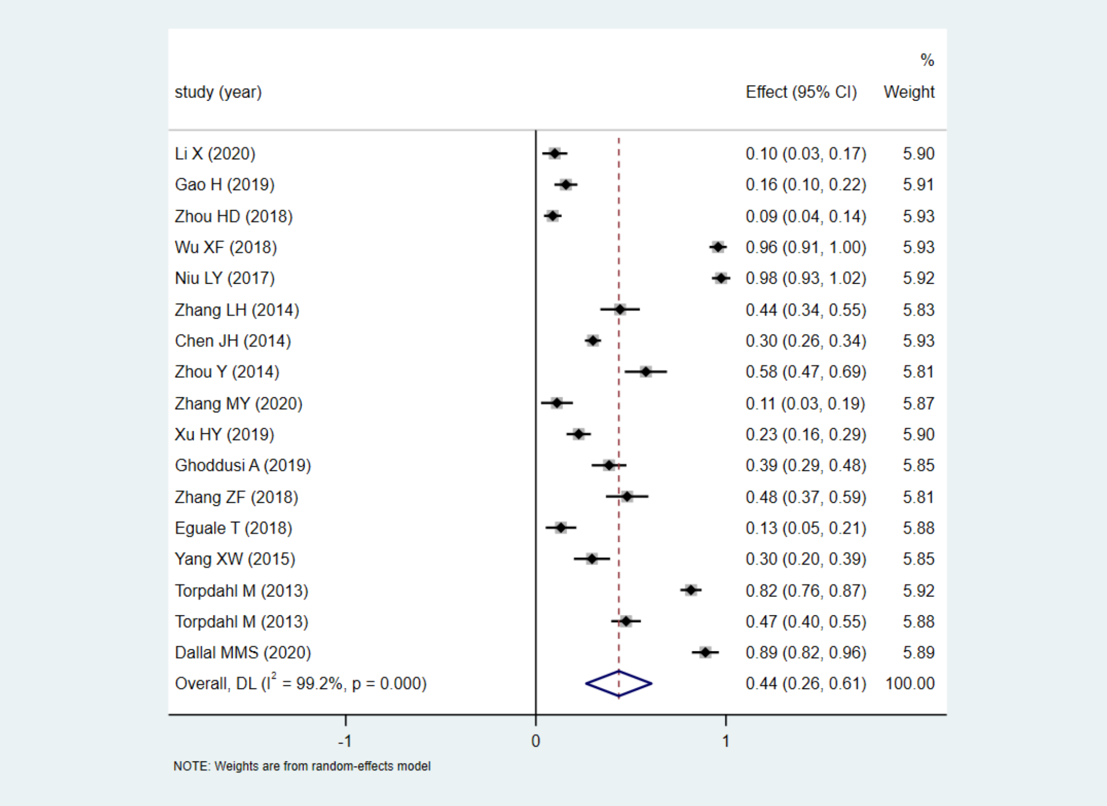

Figure 43. Aminoglycosides resistance in *Salmonella* isolates from human

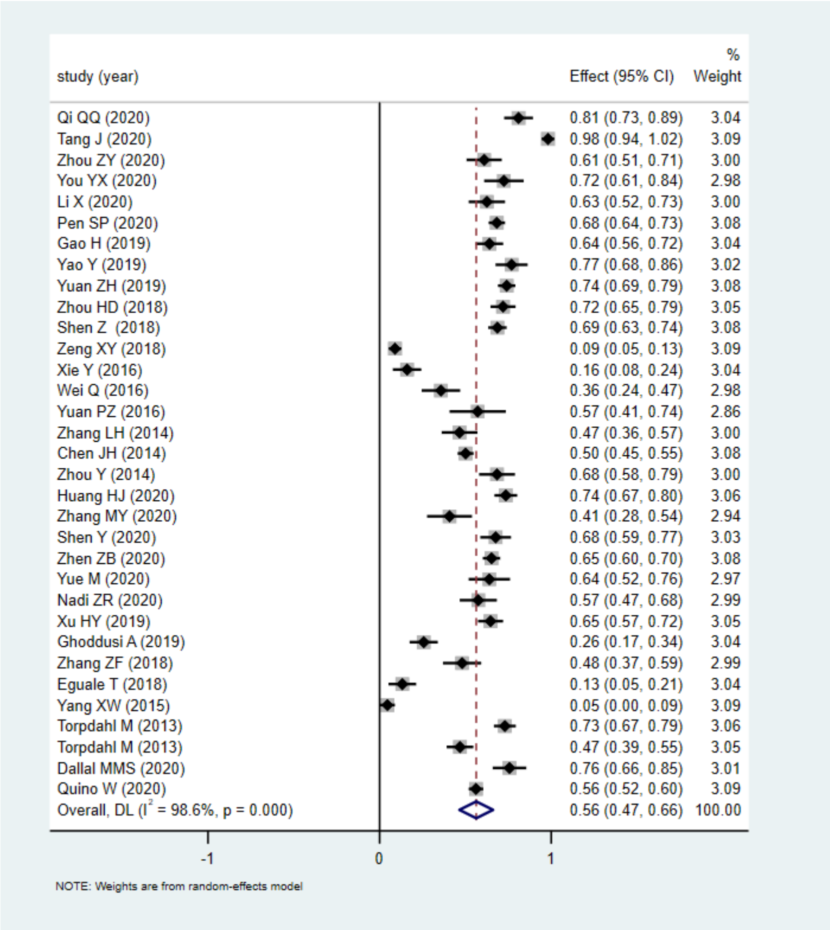

Figure 44. β-Lactams resistance in *Salmonella* isolates from human

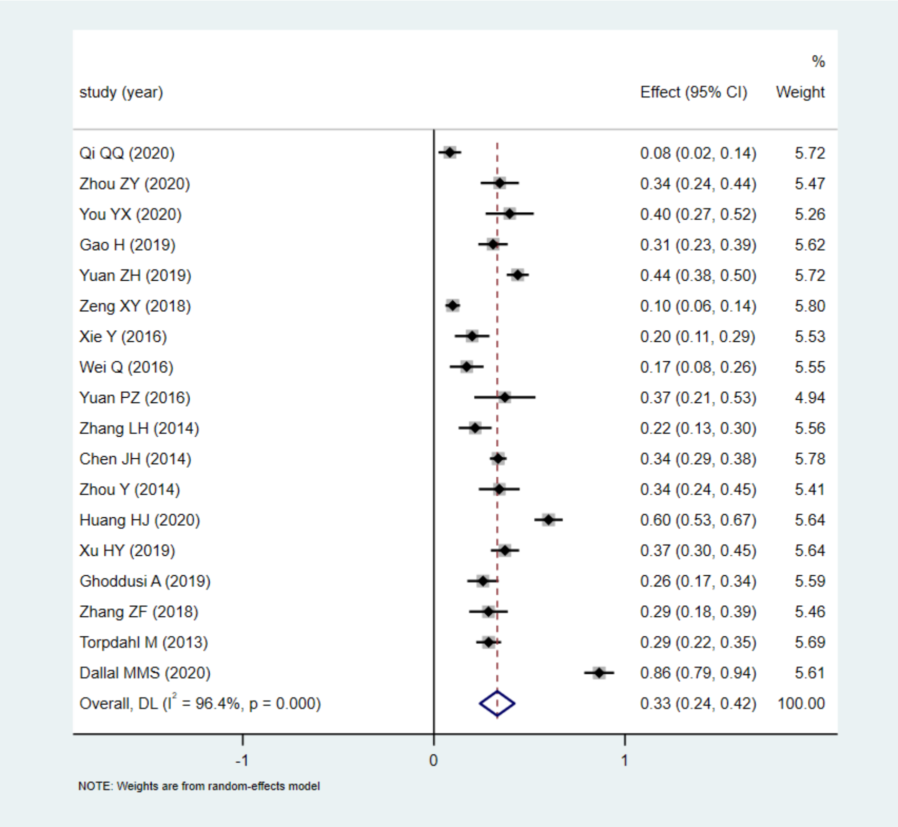

Figure 45. Chloramphenicol resistance in *Salmonella* isolates from human

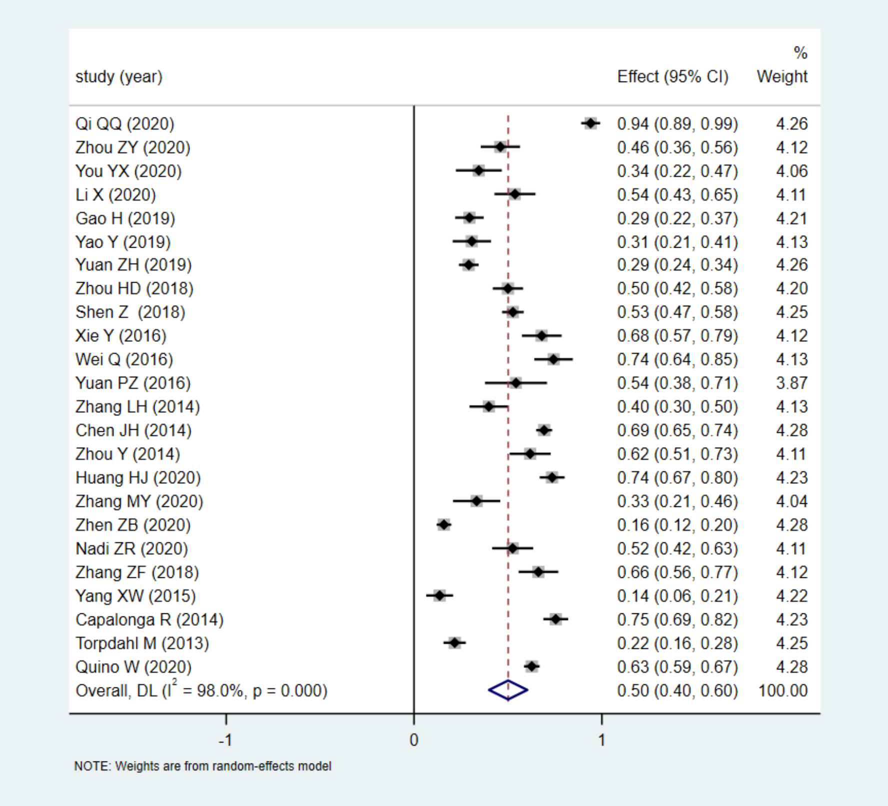

Figure 46. Fluoroquinolones resistance in *Salmonella* isolates from human

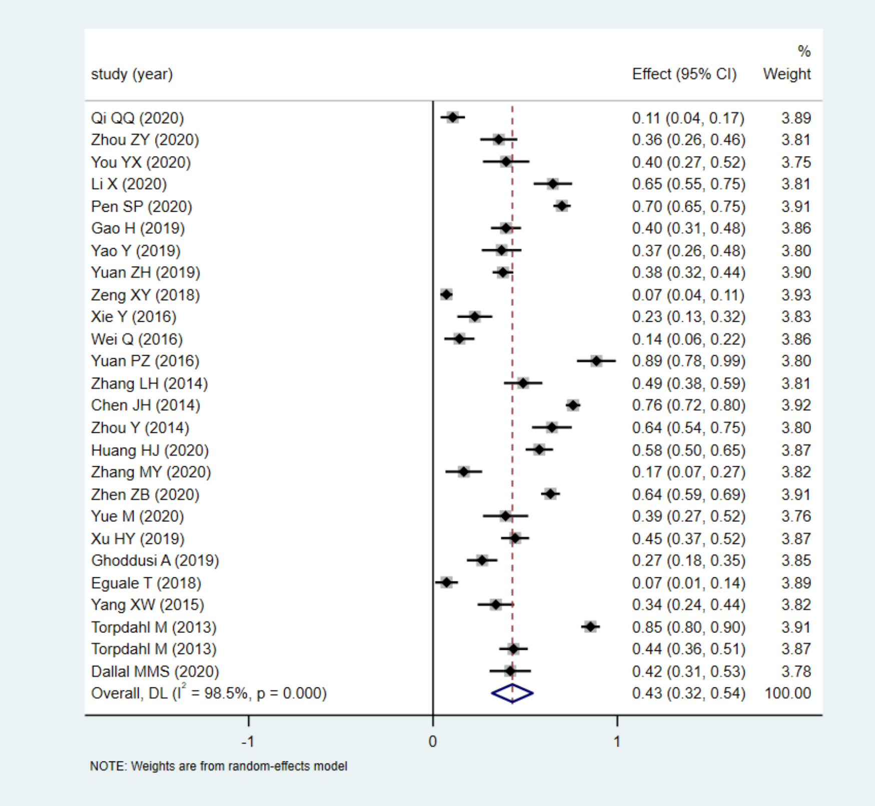

Figure 47. Sulfonamides resistance in *Salmonella* isolates from human

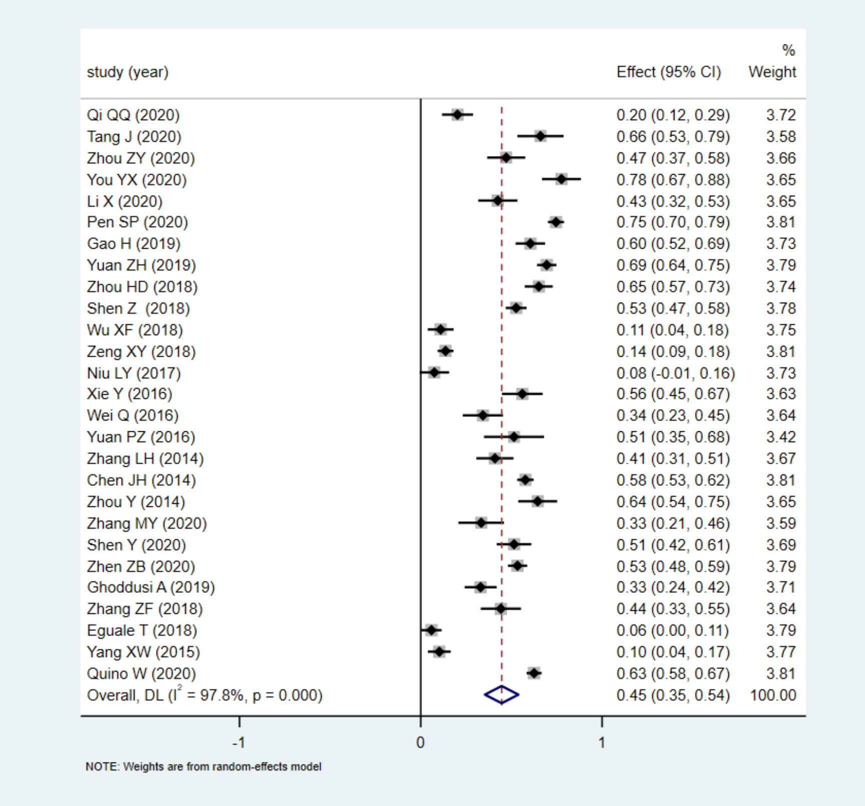

Figure 48. Tetracyclines resistance in *Salmonella* isolates from human

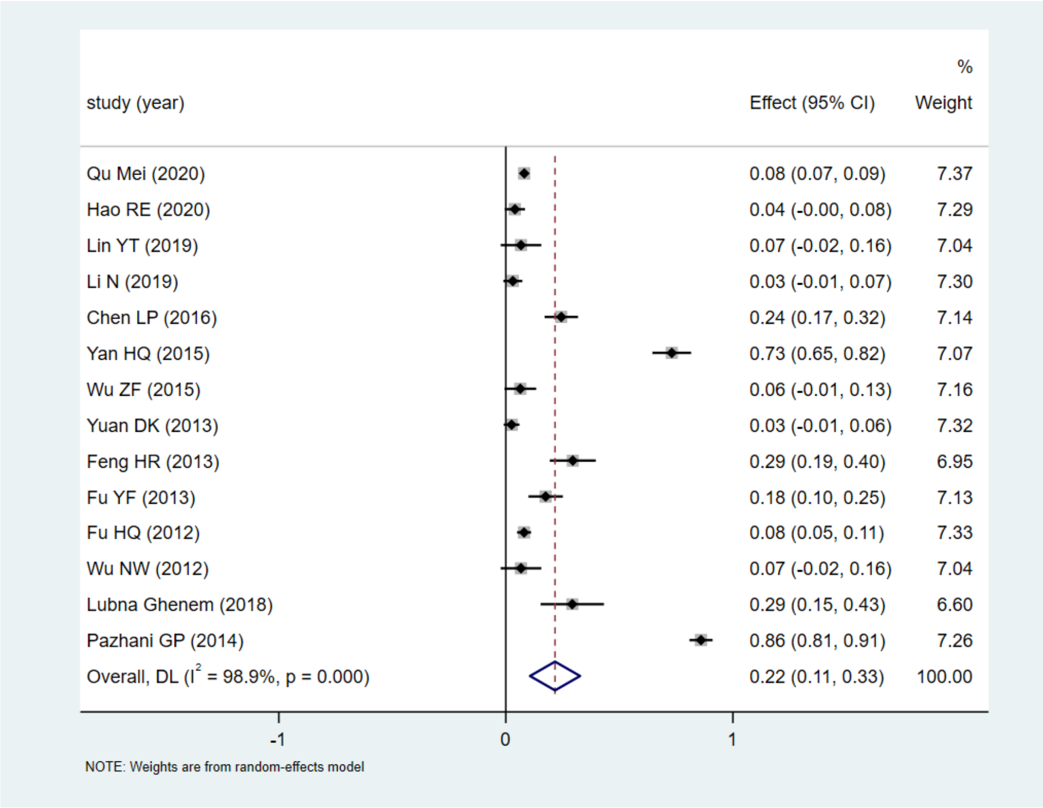

Figure 49. Aminoglycosides resistance in *Vibrio parahaemolyticus* isolates from human

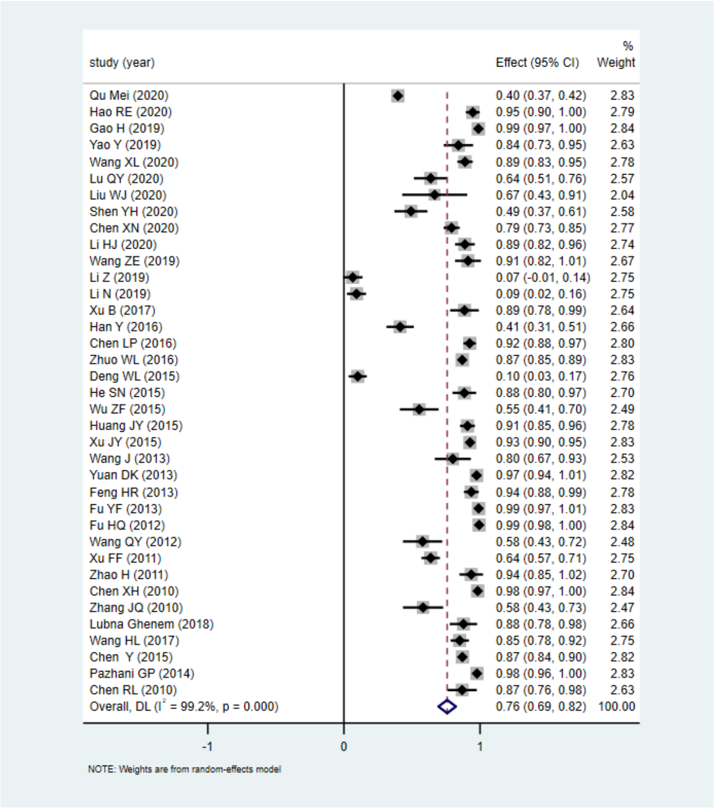

Figure 50. β-Lactams resistance in *Vibrio parahaemolyticus* isolates from human

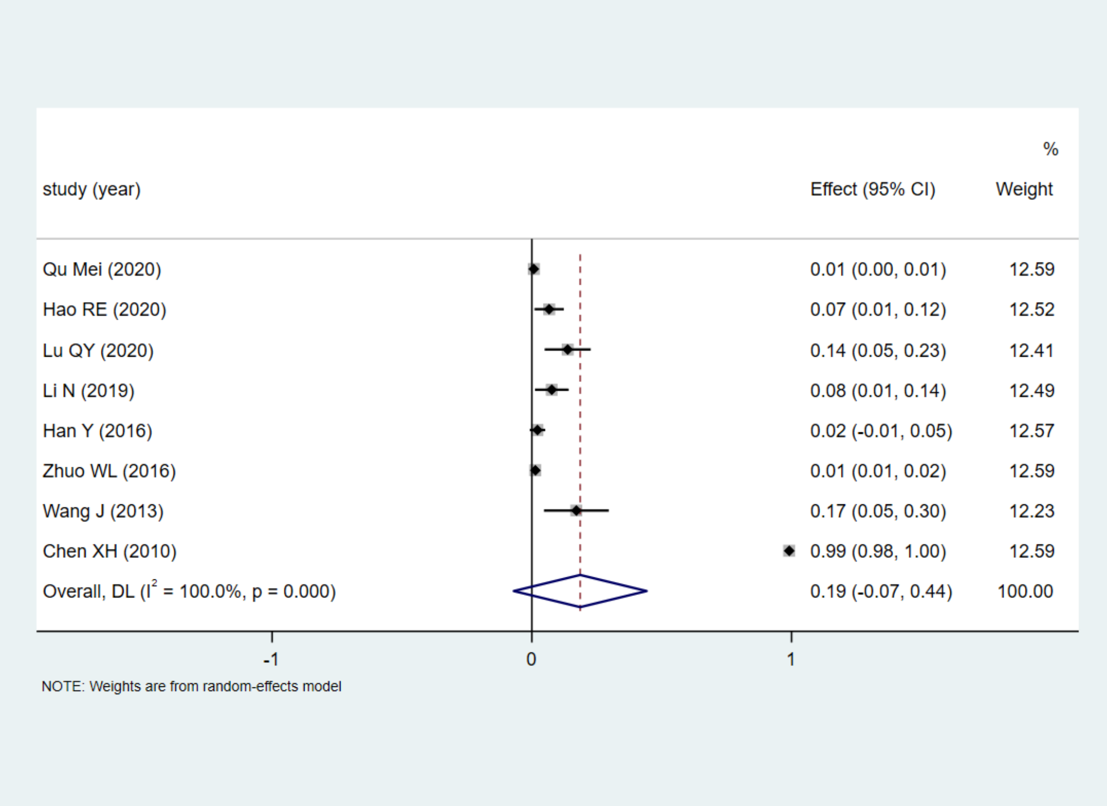

Figure 51. Fluoroquinolones resistance in *Vibrio parahaemolyticus* isolates from human

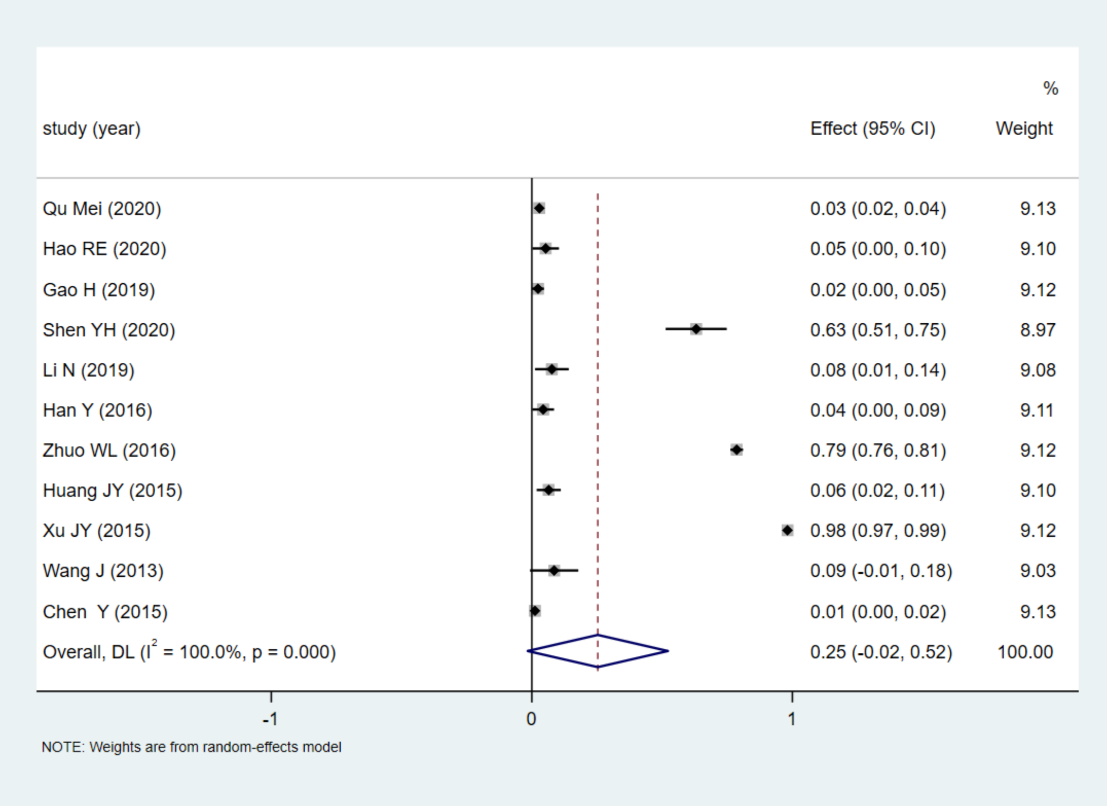

Figure 52. Sulfonamides resistance in *Vibrio parahaemolyticus* isolates from human

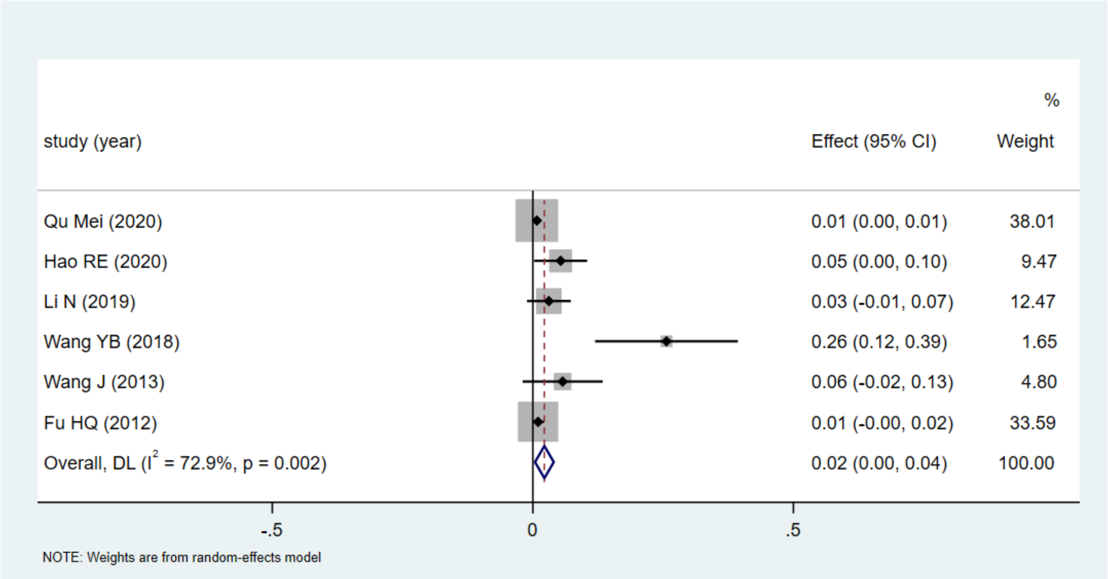

Figure 53. Tetracyclines resistance in *Vibrio parahaemolyticus* isolates from human

Supplementary Funnel plots

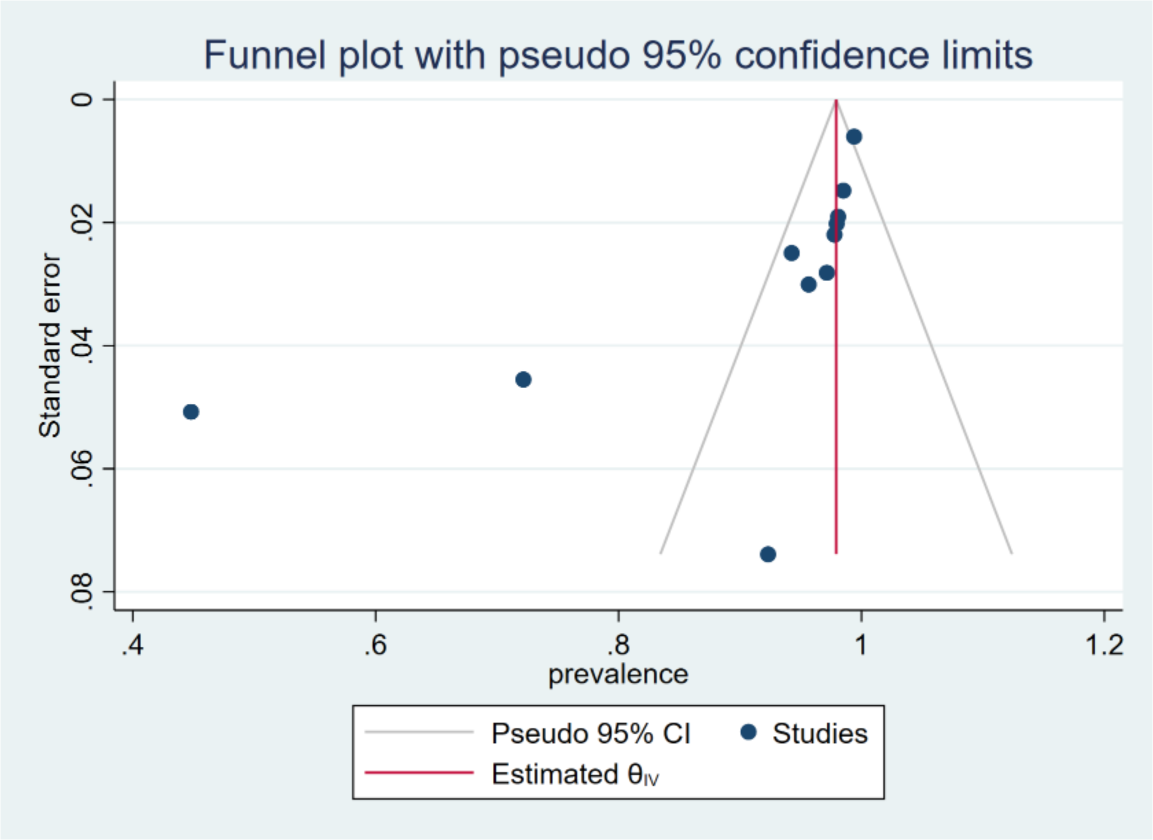

Figure 54. Funnel plot of the meta-analysis of biofilm formation rate in foodborne pathogen retrieved from food samples. (11 studies included. Egger's test for small study effect P<.01). ES,

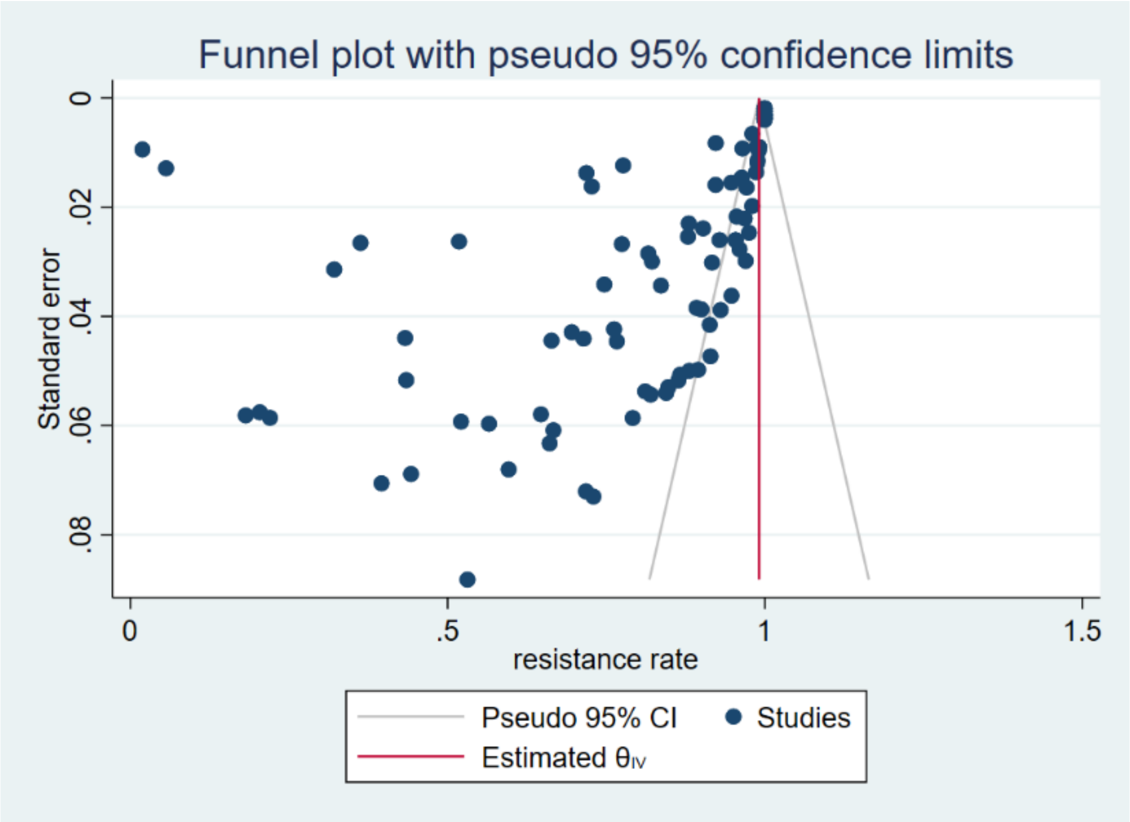

Figure 55. Funnel plot of the meta-analysis of resistance rate of food isolate.

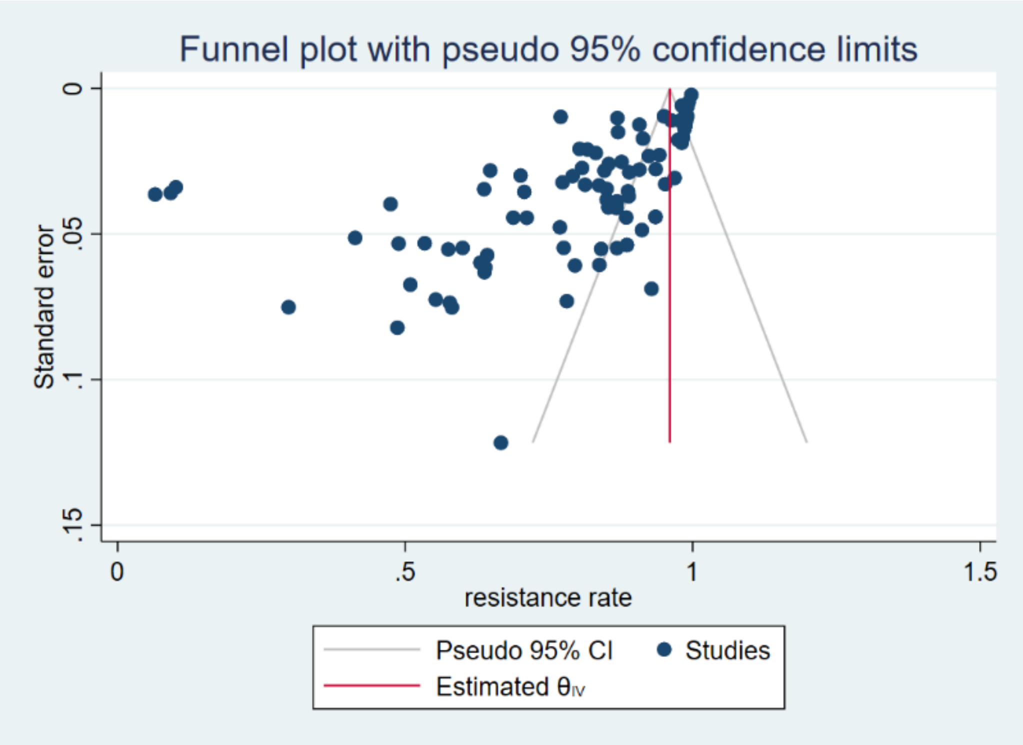

Figure 56. Funnel plot of the meta-analysis of resistance rate of humans isolate.

References for included studies

1. Abass, A., Adzitey, F., and Huda, N. (2020). *Escherichia coli* of Ready-to-Eat (RTE) Meats Origin Showed Resistance to Antibiotics Used by Farmers. *Antibiotics-basel.* 9(12). doi: 10.3390/antibiotics9120869
2. Abdalrahman, L. S., Stanley, A., Wells, H., and Fakhr, M. K. (2015). Isolation, Virulence, and Antimicrobial Resistance of Methicillin-Resistant *Staphylococcus aureus* (MRSA) and Methicillin Sensitive *Staphylococcus aureus* (MSSA) Strains from Oklahoma Retail Poultry Meats. *Int. J. Env. Res. Pub. He*. 12(6), 6148-6161. doi: 10.3390/ijerph120606148
3. Adesokan, H. K., Funso-Adu, K., and Okunlade, O. A. (2020). Foodborne Pathogens on Meat Stored in Major Central Cold Rooms in Ibadan and their Susceptibility to Antimicrobial Agents. *Folia. Veterinaria*. 64(2), 1-10. doi: 10.2478/fv-2020-0011
4. Adzitey, F., Peprah, P. A., Teye, G. A., Somboro, A. M., Kumalo, H. M., and Amoako, D. G. (2020). Prevalence and Antimicrobial Resistance of *Escherichia coli* Isolated from Various Meat Types in the Tamale Metropolis of Ghana. *Int. J. Food. Sci*. 8877196. 10.1155/2020/8877196
5. Adzitey, F., Teye, G. A., and Amoako, D. G. (2020). Prevalence, phylogenomic insights, and phenotypic characterization of *Salmonella* enterica isolated from meats in the Tamale metropolis of Ghana. *Food. Sci. Nut*. 8(7), 3647-3655. doi: 10.1002/fsn3.1647
6. Ali, D. A., Tadesse, W., and Aragaw, E. (2020). Prevalence and Antibiotic Resistance Pattern of *Salmonella* Isolated from Caecal Contents of Exotic Chicken in Debre Zeit and Modjo, Ethiopia. *Int. J. Microbiol.* 2020, 1910630. doi: 10.1155/2020/1910630
7. Amajoud, N., Bouchrif, B., Maadoudi, M. E., Senhaji, N. S., and Harsal, A. E. (2017). Prevalence, serotype distribution, and antimicrobial resistance of *Salmonella* isolated from food products in Morocco. *J. Infect. Dev. Countr.* 11(2), 136-142. doi: 10.3855/jidc.8026
8. An, X. H., Ning, X. B., and Li, Tao. (2010). Contamination,Virulence Genes and Drug Resistance of *Vibrio parahaemolyticus* from Aquatic Products. *Food. Sci.* 31(03), 209-212.
9. Aydin, A., Muratoglu, K., Sudagidan, M., Bostan, K., Okuklu, B., and Harsa, S. (2011). Prevalence and Antibiotic Resistance of Foodborne *Staphylococcus aureus* Isolates in Turkey. *Foodborne Pathog. Dis.* 8(1), 63-69. 10.1089/fpd.2010.0613
10. Baghbaderani, Z. T., Shakerian, A., and Rahimi, E. (2020). Phenotypic and Genotypic Assessment of Antibiotic Resistance of *Staphylococcus aureus* Bacteria Isolated from Retail Meat. *Infect Drug Resist*, 13, 1339-1349. 10.2147/IDR.S241189
11. Bai, Y., Shu-Yao, Y. E., Yan, S. F., Han, H. H., Yu, D. M., Li, Z. G., et al. (2017). Virulence and antimicrobial characteristics of foodborne *Vibrio parahaemolyticus* strains in China in 2015. *J. Food Saf. Qual*. 8(06), 2318-2324.
12. Bai, Y., Zhao, Y. Y., Ye, S. Y., Jiang, T., Wang, W., Pei, X. Y., et al. (2018). Antimicrobial resistance and genetic characteristics of *Vibrio parahaemolyticus* isolated from aquatic products in China. *Chinese Journal of Food Hygiene*, 30(03), 229-234. doi: 10.13590/j.cjfh.2018.03.002
13. Baliga, P. V., Shekar, M., Ahamed, S. T., and Venugopal, M. (2019). Antibiotic resistance pattern and its correlation to the presence of tdh gene and CRISPR-Cas system in *Vibrio parahaemolyticus* strains isolated from seafood. *Indian J. Fish.* 66(2), 100-108. doi: 10.21077/ijf.2019.66.2.86920-14

14. Beshiru, A., Igbinosa, I. H., and Igbinosa, E. O. (2019). Prevalence of Antimicrobial Resistance and Virulence Gene Elements of *Salmonella* Serovars From Ready-to-Eat (RTE) Shrimps. *Front. Microbiol.* 10. doi: 10.3389/fmicb.2019.01613
15. Bi, X. Y. (2015). The Analysis of *Vibrio parahaemolyticus* in Market shellfish of Lianyungang. *Soochow University*.
16. Bissong, M. E. A., Tahnteng, B. F., Ateba, C. N., and Akoachere, J. F. D. K. (2020). Pathogenic Potential and Antimicrobial Resistance Profile of *Staphylococcus aureus* in Milk and Beef from the Northwest and Southwest Regions of Cameroon. *BioMed Res. Int.* 2020(3), 1-12. doi: 10.1155/2020/6015283
17. Bokharaei, N. M., Dallal, M., and Pourmand, M. R. (2020). Rajabi Z. Antibiotic Resistance Pattern and Detection of mecA Gene in *Staphylococcus aureus* Isolated from Iranian Hamburger Samples. *J. Food Qual. Hazards Control*. 7(4), 188-195. doi: 10.18502/jfqhc.7.4.4847
18. Cai, X. F., Cao, B. S., Liu, Y. Q., Hou， C. Y., Zhang, F., and Lu, S. J. (2011). Enterotoxin property and drug resistance analysis of 50 strains of food-borne *Staphylococcus aureus*. *Chinese Journal of Health Laboratory Technology*. 21(02), 419-421.
19. Cai, Y. Q., and Zhi-Long, M. A. (2018). Comparative study on virulence genes and antimicrobial resistance of *Vibrio parahaemolyticus* isolated from different sources in Taizhou. *Modern Preventive Medicine*, 2018, 45(23), 4356-4360.
20. Canizalez-Roman, A., Gonzalez-Nunez, E., Vidal, J.E., Flores-Villasenor, H., and Leon-Sicairos, N. (2013). Prevalence and antibiotic resistance profiles of diarrheagenic *Escherichia coli* strains isolated from food items in northwestern Mexico. *Int. J. Food Microbiol.* 164(1), 36-45. doi: 10.1016/j.ijfoodmicro.2013.03.020
21. Capalonga, R., Ramos, R. C., Both, J. M. C, Soeiro, M. L. T.， Longaray, S. M.， Haas, S., et al. (2014). *Salmonella* serotypes, resistance patterns, and food vehicles of salmonellosis in southern Brazil between 2007 and 2012. *J. Infect Dev. Countr.* 8(07), 811-817. doi: 10.3855/jidc.3791
22. Castro, A., Palhau, C., Cunha, S., Camarinha, S., Silva, J., and Teixeira, P. (2017). Virulence and Resistance profile of *Staphylococcus aureus* isolated from Food. *Acta Alimentaria*. 46(2), 231-237. doi: 10.1556/066.2017.46.2.13
23. Chen, Y., Chen, X., Yu, F., Wu, M., and Li, L. (2016). Serology, virulence, antimicrobial susceptibility and molecular characteristics of clinical Vibrio parahaemolyticus strains circulating in southeastern China from 2009 to 2013. *Clin. Microbiol. Infect.* 22(3), 258.e9-258.e16. doi: 10.1016/j.cmi.2015.11.003
24. Chen, G. L., Ya-Ying, W. U., Jiang, Y. H., Mu, W. T., Guan, L., and Zhang, Y. (2016). Analysis on enterotoxin and antibiotic resistance in *Staphylococcus aureus* isolated from food samples. *J. Med. Pest Control*. 32(07), 757-759+762.
25. Chen, J. H., Jian-Ming, O. U., Yang, J. S., Chen, C. L., Chen W., and Ran, L. (2014). Analysis on the Distribution of Serotypes and Drug Resistance with *Salmonella* Strains, Fujian Province, 2006-2011. *Preventive Medicine Tribune*, 20(02), 81-83+87.
26. Chen, Q., and Xie, S. (2019). Genotypes, Enterotoxin Gene Profiles, and Antimicrobial Resistance of *Staphylococcus aureus* Associated with Foodborne Outbreaks in Hangzhou, China. *Toxins*. 11(6). doi: 10.3390/toxins11060307
27. Chen, X. N., Zhang, X. H., Mu, W. W., and Lin, X. R. (2020). Etiological analysis of *Vibrio*

parahaemolyticus in patients with diarrhea in Ouhai District, Wenzhou. *Chinese Journal of Health Laboratory Technology*. 30(08), 925-927.

28. Chen, Y. H., Jing, L. I, and Tang, Y. D. (2012). Surveillance on Contamination, *Viroluence* and Drug Resistance of *Vibrio parahaemolyticus* in Summer and Autumn Seafood in Nanjing. *Practical Preventive Medicine*, 19(04), 500-502.
29. Chen, Y. Z. (2016). Biological Polymorphism and Antibiotic Resistance Research of *Vibrio parahaemolyticus* in Shandong Province. *Shandong University*.
30. Chen, Y. Z., Shao, K., Guan, B., Hou, P. B., and Zhang, N. H. An analysis on the serotypes and antibiotic sensitivities of foodborne Salmonella in Shandong province from 2003 to 2010. *Chinese Journal of Food Hygiene*. 2012, 24(01), 9-13. doi: 10.13590/j.cjfh.2012.01.016
31. Chen, Z. Q., Bai, J., Wang, S. J., Zhang, X. B., Zhan, Z. Q., Shen, H. Y., et al. (2020). Prevalence, Antimicrobial Resistance, Virulence Genes and Genetic Diversity of Salmonella Isolated from Retail Duck Meat in Southern China. *Microorganisms*, 8(3). doi: 10.3390/microorganisms8030444
32. Cho, Y. S, Lee, M. K., and Hwang, S. H. (2019). Toxin gene profiles, genetic diversity, antimicrobial resistance, and coagulase type of *Staphylococcus aureus* from cream-filled bakery products. *Food Sci. Nutr.* 7(5). doi: 10.1002/fsn3.1011
33. Chon, J. W., Kim, J. H., Lee, S. J., Hyeon, J. Y., and Seo, K. H. (2012). Toxin profile, antibiotic resistance, and phenotypic and molecular characterization of *Bacillus cereus* in Sunsik. *Food Microbiol.* 32(1), 217-222. doi: 10.1016/j.fm.2012.06.003
34. Cui, H. X., Zhang, X. L., Liao, X. G., Yin, G. J., Cui, Y., and Dian, H. U. (2013). Analysis on antibiotic susceptibility of foodborne Staphylococcus aureus and Salmonella in Henan province in 2010. *Modern Preventive Medicine*, 40(02), 320-323. doi: 10.1142/S0219091500000273
35. Cui, H. X., Li, W. J., Zhang, X. L., Y.C, and Li, Y. F. (2014). Analysis of serotypes and drug resistance of food-borne *Listeria monocytogenes* in Henan in 2012. *Chinese Journal of Health Laboratory Technology*, 24(12), 1800-1803.
36. Cui, X., Zhang, X. A., Wang, D., Zhang, Y., Liu, Y. Z., and Chen, Q. (2019). Surveillance on antibiotic resistance of food-borne *Staphylococcus aureus* in Beijing from 2015 to 2018. *J. Food Saf. Qual.* 10(07), 1821-1825.
37. Cunha-Neto, A. D., Carvalho, L. A., Carvalho, R. C. T., Rodrigues, D. D., Mano, S. B., Figueiredo, E. E. D., et al. (2018). *Salmonella* isolated from chicken carcasses from a slaughterhouse in the state of Mato Grosso, Brazil: antibiotic resistance profile, serotyping, and characterization by repetitive sequence-based PCR system - ScienceDirect. *Poult. Sci.* 97(4), 1373-1381. doi: 10.3382/ps/pex406
38. Dallal, M., Motalebi, S., Hossein, M. A., Yazdi, S., Kazem, M., and Abbas, R. F. (2020). Antimicrobial investigation on the multi-state outbreak of salmonellosis and shigellosis in Iran. *Med. J. Islam. Repub. Iran*. 34, 49. doi: 10.34171/mjiri.34.49.
39. Dehkordi, F. S., Basti, A. A., Gandomi, H., Misaghi, A., and Rahimi, E. (2018). Pathogenic *Staphylococcus aureus* in hospital food samples; prevalence and antimicrobial resistance properties. *J. Food Saf.* 38(6). doi: 10.1111/jfs.12501
40. Ed-Dra, A., Filali, F. R., Bouymajane, A., Faouzia, B., Abdellah, E. A., Abdellah, C. et al. (2018). Antibiotic Susceptibility profile of *Staphylococcus aureus* isolated from sausages

in Meknes, Morocco. *Vet. World*. 2018, 11(10). 1459-1465, doi: 10.14202/vetworld.2018.1459-1465

41. Eguale, T., Asrat, D., Alemayehu, H., Nana, I., Gebreyes, W. A., Gunn, J. S., and Engidawork, E. (2018). Phenotypic and genotypic characterization of temporally related nontyphoidal *Salmonella* str ains isolated from humans and food animals in central Ethiopia. *Zoonoses and Public Health*, 65(7), 766-776. doi: 10.1111/zph.12490
42. Elexson, N., Afsah-Hejri, L., Rukayadi, Y., Soopna, P., Lee, H. Y., Zainazor, T. C. T., et al. (2014). Effect of detergents as antibacterial agents on biofilm of antibiotics-resistant *Vibrio parahaemolyticus* isolates. *Food Control*, 2014, 35(1), 378-385. doi: 10.1016/j.foodcont.2013.07.020
43. Elhadi N. (2014). Prevalence and antimicrobial resistance of *Salmonella spp*. in raw retail frozen imported freshwater fish to Eastern Province of Saudi Arabia. *Asian Pacific Journal of Tropical Biomedicine*. 4(3), 234-238. doi: 10.1016/S2221-1691(14)60237-9
44. Fadlallah, S. M., Chehab, M., Cheaito, K., Saleh, M., Ghosn, N., Ammar, W., et al. (2017). Molecular epidemiology and antimicrobial resistance of *Salmonella* species from clinical specimens and food Items in Lebanon. *J. Infect. Dev. Countr.* 11(1), 19-27. doi: 10.3855/jidc.7786
45. Fallah, A. A., Saei-Dehkordi, S. S., Rahnama, M., Tahmasby, H., and Mahzounieh, M. (2012). Prevalence and antimicrobial resistance patterns of *Listeria species* isolated from poultry products marketed in Iran. *Food Control*, 28(2), 327-332. doi: 10.1016/j.foodcont.2012.05.014
46. Fang, W. J, and Tang, Q. L. (2016). Distribution of infectious diarrhea pathogens in Fengxian district of Shanghai. *Chinese Journal of Disinfection*, 33(11), 1090-1092.
47. Farhooumand, P., Soltanpour, M. S, Aminzare, M., and Abbasi, Z. (2020). Prevalence, genotyping and antibiotic resistance of *Listeria monocytogenes* and *Escherichia coli* in fresh beef and chicken meats marketed in Zanjan, Iran. *SSRN Electronic Journal*, 2020, 12(6), 537-546. doi: 10.18502/ijm.v12i6.5028
48. Feng, H. R, Zhao, W., Qin, M., Wen, X. X., Yang, J. Y., Gen, R., et al. (2013). Analysis of the Distribution and Drug Resistance of Acute Diarrhea Pathogenic Bacteria in Fengtai District, Beijing. *Practical Preventive Medicine*, 20(08), 900-903.
49. Fri, J., Njom, H. A., Ateba, C. N., and Ndip, R. N. (2020). Antibiotic Resistance and Virulence Gene Characteristics of Methicillin-Resistant *Staphylococcus aureus* (MRSA) Isolated from Healthy Edible Marine Fish. *Int. J. Microbiol.* 2020(3), 1-9. doi: 10.1155/2020/9803903
50. Fu, F. B., Zhang, X. M., Ma, D., Xu, L. R., Wei, H. Y., Wei, Y. X., and Zeng, J. (2018). Analysis on drug-resistance of *Vibrio parahaemolyticus* isolated from entry and exit aquatic products in Beijing during 2008-2017. *J. Food Saf. Qual.* 9(14), 3835-3840.
51. Fu, H. Q., Su, J. H., Wang, W. Q., Huang, H., and Zhang, H. H. (2018). Analysis of epidemiology and pathogenic characteristics of *Vibrio parahaemolyticus* in diarrhea patients. *Chinese Journal of Health Laboratory Technology*, 22(10), 2457-2459+2463.
52. Fu, Y. F., Sun, Q., Zhu, W. P., Zhu, L. Y., Fei, Y., Ye, C. C., et al. (2013). Analysis of pathogen spectrum surveillance of diarrheal patients in Pudong New Area, Shanghai. *Chinese Journal of Disease Control and Prevention*, 17(03), 246-250.
53. Fu, Y. X., Chen, W. W., Ye, S. Z., Ye, M. H., and Pan, J. R. (2020). Infection status and

etiological characteristics of diarrheogenic *Escherichia coli* among diarrhea patients in sentinel hospitals of Fujian Province in 2019. *Chinese Journal of Food Hygiene*. 32(05), 539-543. doi: 10.13590/j.cjfh.2020.05.013

54. Gao, H., Zhang, D., Zhang, D. Y., Yang, Y. B., and Ye, S. (2019). Analysis of specific bacterial etiology and drug resistance for active monitoring of foodborne diseases in Ningbo in 2017. *Chinese Journal of Health Laboratory Technology*. 29(24), 3049-3052+3055.
55. Gao, Y. L., Dong, P., Li, J. L., Di, Y. R., Ban, F. G., and Zhou, H. X. (2014). Study on Isolation and Drug Resistance of *Staphylococcus aureus* from Dairy Farm. *Animal Husbandry and Feed Science*, doi: 10.16003/j.cnki.issn1672-5190.2014.09.068
56. Ghoddusi, A., Fasaei, B. N., Salehi, T. Z., and Akbarein, H. (2019). Prevalence and characterization of multidrug resistance and variant *Salmonella* genomic island 1 in *Salmonella* isolates from cattle, poultry and humans in Iran. *Zoonoses and Public Health*. 66(6), 587-596, doi: 10.1111/zph.12608
57. Gousia, P., Economou, V., Sakkas, H., and Leveidiotou, S. (2011). Antimicrobial Resistance of Major Foodborne Pathogens from Major Meat Products. *Foodborne pathogens and disease*. doi: 10.1089/fpd.2010.0577
58. Gu, W. H., and Liu, J. (2018). Contamination status of food-borne pathogens in foods sold in Tianjin during 2011-2014. *Henan Journal of Preventive Medicine*. 29(07), 561-564. doi: 10.13515/j.cnki.hnjpm.1006-8414.2018.07.030
59. Guan, W. H., Situ, H. Y., Fang, Z. J., Kong, H. P., and Yang, L. J. (2019). Contamination and drug resistance of *Vibrio parahaemolyticus* in prawns and oyster products in Zhanjiang markets. *Chinese Journal of Health Laboratory Technology*. 29(22), 2689-2691+2694.
60. Han, Y., and Sha, D. (2016). Etiological analysis of *Vibiro parahaemolyticus* from different sources in Wuxi during 2012-2014. *Chinese Journal of Food Hygiene*. 28(06), 795-799. doi: 10.13590/j.cjfh.2016.06.024
61. Harb, A., Habib, I., Mezal, E. H., Kareem, H. S., Laird, T., O'Dea, M., et al. (2018). Occurrence, antimicrobial resistance and whole-genome sequencing analysis of *Salmonella* isolates from chicken carcasses imported into Iraq from four different countries. *Int. J. Food Microbiol*. 284, 84-90. doi: 10.1016/j.ijfoodmicro.2018.07.007
62. Hassena, A. B., Siala, M., Guermazi, S., Zormati, S., Gdoura, R., and Sellami, H. (2019). Occurrence and Phenotypic and Molecular Characterization of Antimicrobial Resistance of *Salmonella* Isolates from Food in Tunisia. *J. food Prot.* 82(7), 1166-1175. doi: 10.4315/0362-028X.JFP-18-607
63. He, L. H., Wu, P. F., Shi, X. L., Chen, M. L., and Fu, Q. H. (2012). Characterization of drug resistance of *Salmonella* from foodborne disease surveillance in Shenzhen. *Chinese Journal of Health Laboratory Technology*. 22(03), 581-583.
64. He, S. N., Chen, M., and Jing, H. Y. (2015). Study on biological characteristics and drug resistance of *Vibrio parahaemolyticus* isolated from patients. *Chinese Journal of Disinfection*. 32(09), 877-878.
65. He, Y., Jin L., Sun, F, Hu, Q. X., and Chen, L. M. (2016). Antibiotic and heavy-metal resistance of *Vibrio parahaemolyticus* isolated from fresh shrimps in Shanghai fish markets, China. *Environ. Sci. Pollut. Res.* 23(15), 15033-15040. doi: 10.1007/s11356-016-

6614-4
66. He, Y., Wang, H., Liu, X. P, and Wang, W. Z. (2014). Survey and study on the pollution and drug resistance of *golden staphylococcus* in fresh milks of Chongqing. *Chinese Journal of Health Laboratory Technology*. 24(19), 2858-2859.
67. Hong, J., Kim, Y., Kim, J., Heu, S., Kim, S.R., Kim, K.P., et al. (2015). Genetic Diversity and Antibiotic Resistance Patterns of *Staphylococcus aureus* Isolated from Leaf Vegetables in Korea. *J. Food Sci.* 80(7), M1526-M1531. doi: 10.1111/1750-3841.12909
68. Hou, L. J. (2013). *Vibrio parahaemolyticus* pollution investigation and virulence isolates factors and drug sensitivity studies in sea Food in Chongqing. *Southwest University*.
69. Huo, Z., Wang, C., Xu, J., Gao, B., and Cao, W. (2017). Study on the homologous and antibiotic susceptibility of foodborne *Listeria monocytogens* in a district of Beijing. *Chinese Journal of Food Hygiene*. 29(03), 289-293. doi: 10.13590/j.cjfh.2017.03.007
70. Hu, Q. X., and Chen, L. (2016). Virulence and Antibiotic and Heavy Metal Resistance of *Vibrio parahaemolyticus* Isolated from Crustaceans and Shellfish in Shanghai, China. *J. Food Protect.* 79(8), 1371-1377. doi: 10.4315/0362-028X.JFP-16-031
71. Hu, Y. J., Liu, C., Wang, M. M., Gan, X., Xu. J., Li, F. Q., et al. (2018). Resistance characteristic analysis for foodborne *Salmonella* isolates from China, 2016. *Chinese Journal of Food Hygiene*. 30(05), 456-461. doi: 10.13590/j.cjfh.2018.05.002.
72. Hu, Y. J., Wang, W., Yan, S. F., Gan, X. L., Yu, H. Y., Liu, C., et al. (2017). Resistance analysis of 1070 *Salmonella* strains isolated from food sample in mainland China,2015. *Chinese Journal of Food Hygiene*. 29(06), 64652. doi: 10.13590/j.cjfh.2017.06.003.
73. Huang, C. M., Nan-Wei, W. U., and Deng, Y. (2016). Analysis of the serotype, virulence gene, drug resistance of *Vibrio parahemolyticus* in Sanya area. *Modern Preventive Medicine*. 43(09), 1665-1669. doi: CNKI:SUN:XDYF.0.2016-09-033.
74. Huang, H. J., Peng, S. L., Zhou, H. D., Liu, H. D., Liu, D. F., Liu, C. W., et al. (2020). Prevalence and drug resistance of *Salmonella* isolated from diarrhea patients in Jiangxi Province in 2016-2018. *Modern Preventive Medicine*. 47(24), 4452-4455. doi: CNKI:SUN:XDYF.0.2020-24-011.
75. Huang, J. Y., Bai-Sheng, L. I., Tan, H. L., Bi-Xia, K. E., Dong-Mei, H. E., and Chang-Wen, K. E. (2015). Pathogenic characteristics of *Vibrio parahaemolyticus* isolated from foodborne diseases in Guangzhou,2010—2013. *Guangdong Medical Journal*. 36(12), 1912-1916. doi: 10.13820/j.cnki.gdyx.2015.12.032.
76. Huang, J. Y.， and Deng, X. (2010). Isolation and resistance analysis of 208 *Bacillus cereuses* from clinic. *Modern Preventive Medicine,* 83-88. doi: CNKI:SUN:XDYF.0.2010-16-057.
77. Huang, M. R., Na, L. I., Huan-Huan, Y. E., and Zhou, B. Y. (2014). Analysis of serotype and drug resistance of *Vibrio parahaemolyticus* isolates from raw pickled seafood. *Chinese Journal of Health Laboratory Technology*. 24(01), 135-137. doi: CNKI:SUN:ZWJZ.0.2014-01-047.
78. Iwabuchi, E., Yamamoto, S., Endo, Y., Ochiai, T., and Hirai, K. (2011). Prevalence of *Salmonella* isolates and antimicrobial resistance patterns in chicken meat throughout Japan. *J. Food Protect.* 74(2), 270-273. doi: 10.4315/0362-028X.JFP-10-215
79. Jaja, I. F., Jaja, C., Chigor, N. V., Anyanwu, M. U., Maduabuchi, E. K., Oguttu, J.W., et al. (2020). Antimicrobial Resistance Phenotype of *Staphylococcus aureus* and *Escherichia*

coli Isolates Obtained from Meat in the Formal and Informal Sectors in South Africa. *BioMed Res. Int.* 2020(11), 1-11. doi: 10.1155/2020/3979482

80. Jamali, H., and Thong, K. L. (2014). Genotypic characterization and antimicrobial resistance of *Listeria monocytogenes* from ready-to-eat foods. *Food Control*. 44, 1-6. doi: 10.1016/j.foodcont.2014.03.038
81. Jamali, H., Paydar, M., Radmehr, B., Ismail, S., and Dadrasnia, A. (2015). Prevalence and antimicrobial resistance of *Staphylococcus aureus* isolated from raw milk and dairy products. *Food Control*. 54, 383-388. doi: 10.1016/j.foodcont.2015.02.013
82. Jeamsripong, S., Khant, W. and Chuanchuen, R. (2020). Distribution of phenotypic and genotypic antimicrobial resistance and virulence genes in *Vibrio parahaemolyticus* isolated from cultivated oysters and estuarine water. *FEMS Microbiol. Ecol.* 96(8):8. doi: 10.1093/femsec/fiaa081
83. Jia, H. Y., Wang, L., Chen, S., Zhang, H., Liang, J. J., Liu, X. G., et al. (2016). Contamination status and pathogenic characteristics of *Vibrio parahaemolyticus* in aquatic products sold in cities of Hunan Province,2010-2013. *Practical Preventive Medicine*. 23(12), 1433-1435. doi: CNKI: SUN: SYYY.0.2016-12-008.
84. Jia, H. Y., Wang, L., and Zhan, H. U. (2013). Contamination Status of Food Borne Pathogenic Bacteria in Instant Food in Hunan and Their Antibiotic Resistance. *Practical Preventive Medicine*. 20(01), 16-18. doi: CNKI: SUN: SYYY.0.2013-01-006.
85. Jiang, H., Yu, T., Yang, Y. T., Yu, S. T., Wu, J. C., Lin, R. M., et al. (2020). Co-occurrence of Antibiotic and Heavy Metal Resistance and Sequence Type Diversity of *Vibrio parahaemolyticus* Isolated From Penaeus vannamei at Freshwater Farms, Seawater Farms, and Markets in Zhejiang Province, China. *Front. Microbiol.* 11-1294. doi: 10.3389/fmicb.2020.01294
86. Jiang, L. X., Yang, M., and Deng, K. J. (2010). Contamination Status and Drug Resistance of *Vibrio parahaemolyticus* in Aquantic Products in Shenzhen. *Occupation and Health*. 26(03), 287-288. doi: 10.13329/j.cnki.zyyjk.2010.03.026.
87. Jiang, Y., Yao, L., Li, F., Tan, Z., Zhai, Y., and Wang, L. (2014). Characterization of antimicrobial resistance of Vibrio parahaemolyticus from cultured sea cucumbers (Apostichopus japonicas). *Lett. Appl. Microbiol.* 2014, 59(2), 147-154. doi: 10.1111/lam.12258
88. Jiang, Y. H., Chu, Y. B., Xie, G., Li, F., Wang, L., Huang, J., et al. (2019). Antimicrobial resistance, virulence and genetic relationship of *Vibrio parahaemolyticus* in seafood from coasts of Bohai Sea and Yellow Sea, China. *Int. J. Food microbiol.* 290, 116-124. doi: 10.1016/j.ijfoodmicro.2018.10.005
89. Jiang, Y. H., Chu, Y. B., Wang, L. Z., Feng-Ling, L. I., Zhai, Y. X., Yao, L. (2018). Analysis of virulence gene and antimicrobial resistance of *Vibrio parahaemolyticus* in fresh shellfish from areas of the Bohai Sea and the Yellow Sea. *Chinese Journal of Health Laboratory Technology*. 28(07) 769-773. doi: CNKI: SUN: ZWJZ.0.2018-07-001.
90. Jiang, Y. H., Yao, L., Jun, L. I., Feng-Ling, L. I., Yi-Guang, L. I., Zhai, Y. X., et al. (2015). Pathogenicity and antimicrobial resistance of *Vibrio parahaemolyticus* from maricultured shellfish. *J. Food Saf. Qual.* 6(9), 3474-3479. doi: 10.19812/j.cnki.jfsq11-5956/ts.2015.09.033.
91. Jiang, Y. H., Yao, B., Li, F. L., Wang, L. Z., Song, C. L., and Zhai, Y. X. (2013). Isolation

and antimicrobial resistance of *Vibrio parahaemolyticus* In farmed marine shrimps from Qingdao markets. *Chinese Journal of Zoonoses*. 29(05), 516-519. doi: CNKI: SUN: ZRSZ.0.2013-05-022.
92. Jiang, Y. H., Lin, Y., Song, C. L., Yuan, H. N., Zhai, Y. X., and Wang, L. Z. (2012). Contamination status of *Vibrio parahaemolyticus* in shellfish from Qingdao markets and their antimicrobial resistance. *Chinese Journal of Health Laboratory Technology*. 22(02), 375-377. doi: CNKI:SUN:ZWJZ.0.2012-02-074.
93. Jiang, Z., Song, Y., Wu, H. Y., Xiao-Wen, X. U., Gong, C. B., and Leng, Q. Y. (2018). Analysis of the pathogenic characteristics and PFGE patterns of *Vibrio parahemolyticus* isolated from different sources in the City of Yantai. *J. Pathogen Biol*. 13(08), 877-881. doi: 10.13350/j.cjpb.180817.
94. Jin, J. H., Li, Z. Y., Zhu, F. Y., Zhang, Q., and Liu, J. Y. (2013). Investigation on contamination of *Vibrio parahaemolyticus* in aquatic products in Gongshu district of Hangzhou city. *Chinese Journal of Health Laboratory Technology*. 23(06), 1596-1598. doi: CNKI:SUN:ZWJZ.0.2013-06-094.
95. Kang, C. H., Shin, Y., and Jang, S. C. (2017). Characterization of *Vibrio parahaemolyticus* isolated from oysters in Korea: Resistance to various antibiotics and prevalence of virulence genes. *Mar. Pollut. Bull.* 118(1-2), 261. doi: 10.1016/j.marpolbul.2017.02.070.
96. Kang, C. H., Shin, Y. J., Yu, H. S., Kim, S., An, S., Park, K., and So, J. S. (2018). Antibiotic and heavy-metal resistance of *Vibrio parahaemolyticus* isolated from oysters in Korea. *Mar. Pollut. Bull.* 135, 69-74. doi: 10.1016/j.marpolbul.2017.02.070
97. Kassem, I. I., Nasser, N. A., and Salibi, J. (2020). Prevalence and Loads of Fecal Pollution Indicators and the Antibiotic Resistance Phenotypes of *Escherichia coli* in Raw Minced Beef in Lebanon. *Foods*, 9(11). doi: 10.3390/foods9111543
98. Kevenk, T. O., and Gulel, G. T. (2016). Prevalence, Antimicrobial Resistance and Serotype Distribution of *Listeria monocytogenes* Isolated from Raw Milk and Dairy Products. *J. Food Saf.* 36(1), 11-18. doi: 10.1111/jfs.12208
99. Kizil, S. (2020). Extended Spectrum Beta-Lactamase (ESβL), AmpC and carbapenemase activities and colistin resistance of Salmonella spp. isolated from food poisoning cases in Turkey. *Turkish J. Vet. Animal Sci.* 44(4), 821-829. doi: 10.3906/vet-2001-31
100. Koo, H. J., and Woo, G. J. (2012). Characterization of Antimicrobial Resistance of *Escherichia coli* Recovered from Foods of Animal and Fish Origin in Korea. *Journal of Food Protect.* 75(5), 966-972. doi: 10.4315/0362-028X.JFP-11-003.
101. Lampugnani, C., Montanhini, M.Taís., Maziero, C. G., Maria, E. M., Nero, L. A., and Bersot, L. S. (2019). Enterotoxins production, biofilm formation and antimicrobial resistance of *Staphylococcus aureus* strains isolated from refrigerated raw cow milk. *Acta Sci-Technol*. 2019, 42: e45231. doi: 10.4025/actascitechnol.v42i1.45231.
102. Lan, T., Liu, H. M., Meng, L., Xing, M. M., Dong, L., Gu, M., et al. (2020). Antimicrobial susceptibility, phylotypes, and virulence genes of *Escherichia coli* from clinical bovine mastitis in five provinces of China. *Food Agric Immunol.* 31(1), 406-423. doi: 10.1080/09540105.2020.1736009.
103. Lapierre, L., Cornejo, J., Zavala, S., Galarce, N., Sánchez, F., Benavides, M. B., et al. (2020). Phenotypic and Genotypic Characterization of Virulence Factors and Susceptibility to Antibiotics in *Salmonella* Infantis Strains Isolated from Chicken Meat:

First Findings in Chile. *Animals*, 10(6), 1049. doi: 10.3390/ani10061049.
104. Lee, L. H., Mutalib, N., Law, W. F., Law, J. W. F., Wong, S. H., and Letchumanan, V. (2018) Discovery on Antibiotic Resistance Patterns of *Vibrio parahaemolyticus* in Selangor Reveals Carbapenemase Producing *Vibrio parahaemolyticus* in Marine and Freshwater Fish. *Front. Microbiol.* 9, 2513. doi: 10.3389/fmicb.2018.02513.
105. Lee, N., Sun, J. M., Kwon, K. Y., Kim, H. J., Koo, M., and Chun, H. S. (2012). Genetic diversity, antimicrobial resistance, and toxigenic profiles of *Bacillus cereus* strains isolated from Sunsik. *J. Food Protect.* 75(2), 225-230. doi: 10.1016/j.ijfoodmicro.2011.02.029.
106. Letchumanan, V., Pusparajah, P., Tan, L. T. H., Yin, W. F., Lee, L. H., and Chan, K.G. (2015). Occurrence and Antibiotic Resistance of *Vibrio parahaemolyticus* from Shellfish in Selangor, Malaysia. *Front. Microbiol.* 6, 1417. doi: 10.3389/fmicb.2015.01417.
107. Li, B. B., Liu, L., Li, S. Z., Jin, J., Liu, C. C., and Zhao, H, R. (2019). Prevalence and characterization of *Staphylococcus aureus* in meats of animal origin in Huai'an. *Chinese Journal of Food Hygiene*. 31(03), 217-221. doi: 10.13590/j.cjfh.2019.03.005.
108. Li, B. B., Liu, C. C., Xing, Y. D., Liu，L., and Hou, H. Y. (2016). Pathogens and their drug resistance in 233 samples of raw meat and 152 samples of freshwater crayfish, Huaian. *Modern Preventive Medicine*. 43(17), 3134-3138. DOI: CNKI:SUN:XDYF.0.2016-17-019.
109. Li, H. J., Chen, Z. H., Ye, B., Ge, R. Y., and Wang, H. M. (2020). Detection and results analysis of fecal *Vibrio parahaemolyticus* in patients with foodborne diarrhea. *Chinese Journal of Health Laboratory Technology*. 30(02), 154-157. DOI: CNKI:SUN:ZWJZ.0.2020-02-008.
110. Li, L. Q., Yao, L., Lin, C. Y., Li, F. L., Qu, M., Wang, L. Z., et al. (2020). Analysis of virulence genes and antimicrobial resistance of *Vibrio parahaemolyticus* isolated from shellfish in 2018. *Chinese Journal of Food Hygiene*. 32(04)， 364-370. doi: 10.13590/j.cjfh.2020.04.003.
111. Li, M. H., Li, Y., and Yan, L. (2019). Antimicrobial resistance and enterotoxigenicity characteristics of foodborne *Staphylococcus aureus* isolates in China. *Chinese Journal of Public Health*. 35(05), 574-578. doi: 10.11847/zgggws1123779.
112. Li, N. (2019). Analysis of Antimicrobial Resistance Phenotype and Gene Distributicon Characterization of *Vibrio parahaemolyticus*. *Shanghai Jiao Tong University.*
113. Li, P., Huang, H., and Zhong, W. B. (2019). Investigation of *Vibrio parahaemolyticus* contamination，drug resistance and virulence genes in shellfish products sold in Haikou . *Chinese Journal of Food Hygiene.* 31(04), 366-370. doi: 10.13590/j.cjfh.2019.04.013.
114. Li, Q. C., Li, Y., Tang, Y. Y., Meng, C., Ingmer, H., and Jiao X. N. (2019). Prevalence and characterization of *Staphylococcus aureus* and *Staphylococcus argenteus* in chicken from retail markets in China. *Food Control,* 96, 158-164. doi: 10.1016/j.foodcont.2018.08.030
115. Li, S. J., Wang, S. D., Cao, T. H., Yi, D. X., Chen, S. Z., and Liu G. H. (2016). Detection and result analysis of 41 strains to *E. coli* bacteria's drug susceptibility test. *Chinese J. Pub. Health Engineer*. 15(02), 127-129. DOI: CNKI:SUN:ZGWX.0.2016-02-010.
116. Li, S. J. (2016). Detection and drug resistance of pathogenic bacteria in feces of foodborne disease patients in Tonghua. *Chinese J. Microecol*. 28(12), 1389-1392. DOI： 10.13381/j.cnki.cjm.201612007.

117. Li, X., Yu, J. L., Qiao, X. F., Wu, J. J., An, N., Xia, Y.J., et al. (2020). Distribution and pathogenic characteristics of *Salmonella* in food and foodborne diseases. *Practical Preventive Medicine*. 27(07), 801-806. DOI: CNKI:SUN:SYYY.0.2020-07-010.
118. Li, Y. C., Pan, Z. M., Kang, X. L., Geng, S. Z., Liu, Z. Y., Cai, Y. Q.,et al. (2014). Prevalence, Characteristics, and Antimicrobial Resistance Patterns of *Salmonella* in Retail Pork in Jiangsu Province, Eastern China. *J. Food protect.* 77(2), 236-245. doi: 10.4315/0362-028X.JFP-13-269
119. Li, Y. H., Luo, Q., Shi, X., Lin, Y. M., Qiu, Y. Q., Lv, D. Y., et al. (2017). Phenotypic and Genotypic Characterization of Clinical Enterotoxigenic *Escherichia coli* Isolates from Shenzhen, China. *Foodborne Pathogens Dis.* 14(6), 333-340. doi: 10.1089/fpd.2016.2233
120. Li, Y. P., Xie, T. F., Pang, R., Wu, Q. P., Zhang, J. M., Lei, T., et al. (2017). Food-Borne *Vibrio parahaemolyticus* in China: Prevalence, Antibiotic Susceptibility, and Genetic Characterization. *Front. Microbiol.* 11, e1670. doi: 10.3389/fmicb.2020.01670
121. Li, Y. T., Gong, Y. W., and Liu, G. H. (2015). Analysis of drug resistance of foodborne *Staphylococcus aureus* in Jilin in 2014. *Chinese Journal of Health Laboratory Technology*. 25(21), 3772-3774. DOI: CNKI:SUN:ZWJZ.0.2015-21-064.
122. Li, Z., Li, Y., Jing, H. B., Wang Y. B., Zhu, M. J., and Zhang, H. (2019). Analysis on drug resistance of human *Vibrio parahaemolyticus* isolated from Shunyi during 2015-2016. *Capital J. Public Health*. 13(01), 33-36. DOI: 10.16760/j.cnki.sdggws.2019.01.018.
123. Lin, Y. T., and Mo, L. J. (2019). Analysis of serotype distribution and drug resistance of *Vibrio parahaemolyticus* in Sanya, Hainan, 2015-2017. *China Tropical Medicine*.19(05), 486-488. DOI: 10.13604/j.cnki.46-1064/r.2019.05.20.
124. Liu, J., Wu, Y., and Zhang, J. (2018). Serotype and drug resistance of food-borne *Salmonella* in 342 commercial available raw meat in Zibo. *Modern Preventive Medicine*. 45(08), 1508-1511+1527. DOI: CNKI:SUN:XDYF.0.2018-08-044.
125. Li, L., Han, X., Wang, Z. W., Wang, Q., Zhao, X. J., Chen, X., et al. (2017). Analysis of antimicrobial resistance of isolated *Salmonella* spp. from import and export food in Beijing, 2004-2012. *Chinese Journal of Food Hygiene*. 29(06), 653-657. DOI: 10.13590/j.cjfh.2017.06.004.
126. Liu, S., Kilonzo-Nthenge, A., Nahashon, S. N., Pokharel, B., Mafiz, A., and Nzomo, M. (2020). Prevalence of Multidrug-Resistant Foodborne Pathogens and Indicator Bacteria from Edible Offal and Muscle Meats in Nashville, Tennessee. *Foods*. 9(9), 1190. doi: 10.3390/foods9091190.
127. Liu, W. J., Song, Y., Xu, Y. C., Li, Y., Wang, Y. Q., Zhou, X. N., et al. (2020). Pathogenic characteristics and traceability analysis of *Vibrio parahaemolyticus* in foodborne diseases in Yantai. *J. Food Saf. Qual.* 11(09), 2986-2991. DOI: 10.19812/j.cnki.jfsq11-5956/ts.2020.09.056.
128. Liu, Y., Gu, Q. F., Liu, C., Zhu, Y. Y., Liu, H., Chen, M., et al. (2018). Serotyping, molecular subtyping and drug resistance patterns of *Salmonella* isolates from retail meat in Shanghai, 2016. *Chinese Journal of Food Hygiene*. 30(02), 132-138. DOI: 10.13590/j.cjfh.2018.02.002.
129. Long, Z. D., Jiang, Y. H., Chen, C. J., Pu, C. R., Jiang, Y., Du, J. L., et al. (2020). Antimicrobial resistance and multilocus sequence typing analysis of *Vibrio parahaemolyticus* from seafood in Chengdu. *Chinese Journal of Food Hygiene*. 32(01),

15-19. DOI: 10.13590/j.cjfh.2020.01.003.

130. Lopatek, M., Wieczorek, K., and Osek, J. (2015). Prevalence and Antimicrobial Resistance of *Vibrio parahaemolyticus* Isolated from Raw Shellfish in Poland. *J. Food Protect.* 2015, 78(5), 1029-1033. doi: 10.4315/0362-028X.JFP-14-437.
131. Lv, Q. Y., Liu, D. P., Luan, M. C., and Bo, Z. J. (2020). Analysis on drug resistance and virulence genes of *Vibrio parahaemolyticus*, Dalian city, 2018. *Preventive Medicine Tribune*. 26(09), 652-654. doi: 10.16406/j.pmt.issn.1672-9153.2020.09.004.
132. Lu, Y., Chen, W. Y., Liu, H. Q., Xie, J., Zhao, Y., Sun, X. H. (2016). Serveillance and analysis on drug- resistance of *Vibrio parahaemolyticus* isolated from the commercial aquatic products in markets of Shanghai. *Sci. Technol. Food Ind.* 37(19), 271-275. doi: 10.13386/j.issn1002-0306.2016.19.045.
133. Lv, G. P., Wang, X., Wei, X. P., Li, J. S., and Feng, P. (2013). Drug resistance and *mecA* gene analysis in foodborne *Staphylococcus aureus*. *J. Env. Health*. 30(12), 1089-1091. doi: 10.16241/j.cnki.1001-5914.2013.12.032.
134. Ma, Z. L., and Cai, Z. (2011). Analysis of bacterial spectrum and drug resistance of foodborne pathogens in commercial food in Taizhou. *Chinese Journal of Health Laboratory Technology*. 21(09), 2297-2299+2302. doi: CNKI:SUN:ZWJZ.0.2011-09-075.
135. Matallah, A. M., Bouayad, L., Boudjellaba, S., Mebkhout, F., Hamdi, T. M., and Ramdani-Bouguessa, N. (2019). *Staphylococcus aureus* isolated from selected dairies of Algeria: Prevalence and susceptibility to antibiotics. *Vet. World*. 12(2), 205-210. doi: 10.14202/vetworld.2019.205-210
136. Mezali, L., and Hamdi, T. M. (2012). Prevalence and Antimicrobial Resistance of *Salmonella* Isolated from Meat and Meat Products in Algiers (Algeria). *Foodborne Pathogens Dis.* 9(6), 522-529. doi: 10.1089/fpd.2011.1032.
137. Nadi, Z. R., Salehi, T. Z., Tamai, I. A., Foroushan, A. R., Sillanpaa, M., and Dallal, M. M. S. (2020). Evaluation of antibiotic resistance and prevalence of common *Salmonella* enterica serovars isolated from foodborne outbreaks. *Microchem. J.* 155, 104660. doi: 10.1016/j.microc.2020.104660
138. Nemati, V., Khomeiri, M., Mahoonak, A. S., and Moayedi, A. (2020). Prevalence and Antibiotic Susceptibility of Listeria Monocytogenes Isolated from Retail Ready-to-Eat Meat Products in Gorgan, Iran. *Nut. Food Sci. Res.* 7(1), 41-46. DOI: 10.29252/nfsr.7.1.41.
139. Niu, L. Y., Qin, L. Y., Xu, B. H., Wang, Y., Yang, X. Y., Xun, D. X., et al. (2017). Characterization of antimicrobial resistances and molecular characteristics of 40 strains of *Salmonella* from food poisoning in Shijiazhuang in 2011-2016. *Chinese Journal of Food Hygiene*. 29(05), 539-543. DOI: 10.13590/j.cjfh.2017.05.004.
140. Obaidat, M. M., Bani Salman, A. E., Lafi, S. Q., and Al-Abboodi A. R. (2015). Characterization of *Listeria monocytogenes* from three countries and antibiotic resistance differences among countries and Listeria monocytogenes serogroups. *Lett. Appl. Microbiol.* 60(6), 609-614. DOI: 10.1111/lam.12420.
141. Obaidat, M. M., Bani Salman, A. E., and Lafi, S. Q. (2015). Prevalence of *Staphylococcus aureus* in Imported Fish and Correlations between Antibiotic Resistance and Enterotoxigenicity. *J. food protect.* 78(11), 1999-2005. DOI: 10.4315/0362-028X.JFP-15-104.

142. Oh, E. G., Son, K. T., Yu, H., Lee, T. S., Lee, H. J., Shin, S., et al. (2011). Antimicrobial resistance of *Vibrio parahaemolyticus* and *Vibrio alginolyticus* strains isolated from farmed fish in Korea from 2005 through 2007. *J. Food Protect.* 74(3), 380-386. doi: 10.4315/0362-028X.JFP-10-307.
143. Ottaviani, D., Leoni, F., Talevi, G., Masini, L., Santarelli, S., Rocchegiani, E., et al. (2013). Extensive investigation of antimicrobial resistance in *Vibrio parahaemolyticus* from shellfish and clinical sources, Italy. *Int. J. Antimicrob. Agents*. 42(2), 191-193. DOI: 10.1016/j.ijantimicag.2013.05.003.
144. Park, K. M., Jeong, M., Park, K. J., and Koo, M. (2018). Prevalence, Enterotoxin Genes, and Antibiotic Resistance of *Bacillus cereus* Isolated from Raw Vegetables in Korea. *J. Food Protect.* 81(10), 1590-1597. doi: 10.4315/0362-028X.JFP-18-205.
145. Pazhani, G. P., Bhowmik, S. K., Ghosh, S., Guin, S., Dutta, S., Rajendran, K., et al. (2014). Trends in the Epidemiology of Pandemic and Non-pandemic Strains of *Vibrio parahaemolyticus* Isolated from Diarrheal Patients in Kolkata, India. *Plos Neglect. Trop. Dis.* 8(5), e2815. doi: 10.1371/journal.pntd.0002815.
146. Pen, S. P., Li, B., Liao, G. D., Xu, M. Q., and Huang, S. F. (2020). Serotyping and drug resistance of food-borne *Salmonella* in children in Maoming, Guangdong. *China Tropical Medicine*. 20(07), 661-665. doi: 10.13604/j.cnki.46-1064/r.2020.07.18.
147. Pu, S. H., Fei, W., and Ge, B. L. (2011). Characterization of toxin genes and antimicrobial susceptibility of *Staphylococcus aureus* isolates from Louisiana retail meats. Foodborne *Pathogens Dis.* 8(2), 299-306. doi: 10.1089/fpd.2010.0679.
148. Puig-Pea, Y., Leyva-Castillo, V., Tejedor-Arias, R., Illnait-Zaragozi, M. T., Abodela-Lopez, N., Camejo-Jardines, A., et al. (2020). Antimicrobial Resistance in Bacteria Isolated from Foods in Cuba. *MEDICC Rev*. 22(3), 40-45. Doi: 10.37757/MR2020.V22.N3.9.
149. Pungpian, C., Sinwat, N., Angkititrakul, S., Sinwat, N., Angkititrakul, S., Prathan, R.,et al. (2020). Presence and Transfer of Antimicrobial Resistance Determinants in *Escherichia coli* in Pigs, Pork, and Humans in Thailand and Lao PDR Border Provinces. *Microb. drug Resist.* 27(4), 571-584. doi: 10.1089/mdr.2019.0438.
150. Qi, Q. Q., Sha, D., Li., J., Zhao, X. F., Zheng, D. Y., and Guan, H. X. (2020). Drug resistance and molecular types of *Salmonella* Enteritidis in Wuxi, Jiangsu, 2012–2018. *Dis. Surveill*. 35(12),1146-1151. DOI: 10.3784/j.issn.1003-9961.2020.12.018.
151. Qin, S., Shen, Y., Ma, K., and Huo, X. (2020). Epidemiological characteristics and drug resistance of diarrheal *Escherichia coli* in foodborne diseases in Jiangsu, 2018-2019. *Mod. Prev. Med*. 47(21), 3884-3888. doi: CNKI:SUN:XDYF.0.2020-21-014.
152. Qin, S., Shen, Y., Zhou, Y. J., Ma, K., and Huo, X. (2020). Virulence genotype distribution and drug resistance characterization of diarrheagenic *Escherichia coli* from children with foodborne diseases in Jiangsu province in 2016. *J. Food. Saf. Food. Qual*. 2020, 11(06), 2019-2024. doi: 10.19812/j.cnki.jfsq11-5956/ts.2020.06.059.
153. Qu, M., Tian, Y., Huang, Y., Liu B. W. Zhang, X., Lv, B., et al. (2020). Serotype distribution and drug resistance analysis on clinical isolates of *Vibrio parahaemolyticus* in Beijing, 2010 — 2019. *Capital Journal of Public Health*. 14(06), 285-290. doi: 10.16760/j.cnki.sdggws.2020.06.003.
154. Que, F. X., Ye, Y. L., Yang, B. W., Zhong, H. M., Han, D. F., and Yu, D. D. (2020).

Analysis of pathogenic characteristics of *Vibrio parahaemolyticus* colonized in aquatic products from Jinshan Distric, Shanghai during 2017—2018. *Chinese Journal of Health Laboratory Technology*. 30(16), 1964-1966. doi: CNKI:SUN:ZWJZ.0.2020-16-013.

155. Quino, W., Hurtado, C. V., Meza, A. M., Zamudio, M. L., and Gavilan, R. G. (2020). Patrones de resistencia a los antimicrobianos en serovares de *Salmonella* enterica en Perú, 2012-2015. *Rev. Chil. Infectol*. 2020, 37(4), 395-401. http://dx.doi.org/10.4067/S0716-10182020000400395
156. Ramadan, H., Jackson, C. R., Frye, J. G., Hiott, L. M., Samir, M., Awad, A., et al. (2020). Antimicrobial Resistance, Genetic Diversity and Multilocus Sequence Typing of *Escherichia coli* from Humans, Retail Chicken and Ground Beef in Egypt. *Pathogens*. 9(5), 357. doi: 10.3390/pathogens9050357.
157. Ranjbar, R., Safarpoor Dehkordi, F., Sakhaei Shahreza, M., and Rahimi, E. (2018). Prevalence, identification of virulence factors, O-serogroups and antibiotic resistance properties of Shiga-toxin producing *Escherichia coli* strains isolated from raw milk and traditional dairy products. *Antimicrob. Resist. Infect. control*. 7, e53. DOI: 10.1186/s13756-018-0345-x.
158. Richter, L., Du Plesis, E., Duvenage, S., and Korsten, L. (2020). High prevalence of multidrug resistant *Escherichia coli* isolated from fresh vegetables sold by selected formal and informal traders in the most densely populated Province of South Africa. *J. Food. Sci*. 86(1), 161-168. doi: 10.1111/1750-3841.15534.
159. Romero-Barrios, P., Deckert, A., Parmley, E. J., and Leclair, D. (2020). Antimicrobial Resistance Profiles of *Escherichia coli* and *Salmonella* Isolates in Canadian Broiler Chickens and Their Products. *Foodborne Pathog*. 17(11), 672-678. doi: 10.1155/2021/6759046.
160. Rong, D. L., Wu, Q. P., Xu, M. F., Zhang, J. M., and Yu, S. B. (2017). Prevalence, Virulence Genes, Antimicrobial Susceptibility, and Genetic Diversity of *Staphylococcus aureus* from Retail Aquatic Products in China. *Front. Microbiol*. 8, 714. doi: 10.3389/fmicb.2017.00714.
161. Ryu, A. R., Park, K., Kim, S. H., Ham, I. T., Kwon, J. Y., Kim, J. H., et al. (2017). Antimicrobial Resistance Patterns of *Escherichia coli* and *Vibrio parahaemolyticus* Isolated from Shellfish from the West Coast of Korea. *Korean J. Fish. Aquat. Sci*. 50(6), 662-668. doi: 10.5657/KFAS.2017.0662.
162. Ryu, A. R., Mok, J. S., Lee, D. E., Kwon, J. Y., and Park, K. (2019). Occurrence, virulence, and antimicrobial resistance of *Vibrio parahaemolyticus* isolated from bivalve shellfish farms along the southern coast of Korea. *Env. Sci. Pollut. Res*. 26(20), 21034-21043. doi: 10.1007/s11356-019-05426-1.
163. Mahmoodi Sadr, M., Shakerian, A., Rahimi, E., and Momtaz, H. (2019). Antibiotic resistance properties and genotypic characterization of enterotoxins in the *Staphylococcus aureus* strains isolated from traditional sweets. *J. Food Saf*. 39, e12573. doi: 10.1111/jfs.12573.
164. Savariraj, W. R., Ravindran, N. B., Kannan, P., Paramasivam, R., Senthilkumar, T. M. A., Kumarasamy, P., et al. (2018). Prevalence, antimicrobial susceptibility and virulence genes of *Staphylococcus aureus* isolated from pork meat in retail outlets in India. *J Food Saf*. e12589. doi: 10.1111/jfs.12589.

165. Sharma, S. K., Yadav, R., Mehta, S. C., and Kataria, A. K. (2020). Detection and analysis of antibiotic resistance variability among *Staphylococcus aureus* isolates from animal and human sources. *VET ARHIV*. 90(5), 493-508. DOI: 10.24099/vet.arhiv.0556.
166. Shen, J. L., Zhao, L. N., Han, W., Xu, Z. J., Jiang, Y., Yang, J. L., et al. (2021). Antimicrobial Resistance, Virulence Gene and Genetic Characteristics of *Vibrio parahaemolyticus* Isolates from Aquatic Products Imported to Shanghai. *Food Sci*. 42(08), 264-269.
167. Shen, Y., Qin, S., and Huo, X. (2020). Study on the infection rate and drug resistance of *Salmonella* in children with diarrhea in some areas of Jiangsu province in 2019. *J. Food Ssf. Food Qual*. 11(15), 5150-5155. doi: 10.19812/j.cnki.jfsq11-5956/ts.2020.15.039.
168. Shen, Y. H., Yan, W., Zhu, X. J., Xu, D. X., and Wu, X. F. (2020). Molecular typing and drug resistance characteristics of 65 strains of *Vibrio arahaemolyticus*. *Chinese Journal of Health Laboratory Technology*. 2020, 30(08): 931-933+940. DOI: CNKI:SUN:ZWJZ.0.2020-08-010.
169. Shi, C. P., Yu, Z. N., Ho, H., Wang, J., Wu, W., Xing, M. R., et al. (2020). Antimicrobial Resistance Patterns, and Genetic Characterization of *Staphylococcus aureus* Isolated from Raw Milk in the Dairy Farms over Two Seasons in China. *MICROB DRUG RESIST*. 27(1), 99-110. doi: 10.1089/mdr.2019.0358.
170. Wu, S., Wu, Q. P., Zhang, J. M., and Chen, M. T. (2015). *Listeria monocytogenes* Prevalence and Characteristics in Retail Raw Foods in China. *PLOS ONE*. 10(8), e0136682. doi: 10.1371/journal.pone.0136682.
171. Silveira-Filho, V. M., Luz, I. S., Campos, A. P. F., Silva, W. M., Barros, M. P., Medeiros, E. S., et al. (2014). Antibiotic resistance and molecular analysis of *Staphylococcus aureus* isolated from cow's milk and dairy products in northeast Brazil. *J. Food Prot*. 77(4), 583-591. doi: 10.4315/0362-028X.JFP-13-343.
172. Skowron, K., Waecka-Zacharska, E., Wiktorczyk-Kapischke, N., Skowron, K. J., Grudlewska-Buda, K., Bauza-Kaszewska, J., et al. (2020). Assessment of the Prevalence and Drug Susceptibility of *Listeria monocytogenes* Strains Isolated from Various Types of Meat. *Foods*. 9(9), 1293. doi: 10.3390/foods9091293.
173. Parvin, M. S., Talukder, S., Ali, M. Y., Chowdhury, E. H., Rahman, T., and Islam, M. T. (2020). Antimicrobial Resistance Pattern of *Escherichia coli* Isolated from Frozen Chicken Meat in Bangladesh. *Pathogens*. 9(6), 420. doi: 10.1080/03079457.2016.1168515.
174. Su, C. L., and Chen, L. M. (2020). Virulence, resistance, and genetic diversity of *Vibrio parahaemolyticus* recovered from commonly consumed aquatic products in Shanghai, China. *MAR POLLUT BULL*. 160(3), 111554. doi: 10.1016/j.marpolbul.2020.111554.
175. Su. J., Wu, Y., Yang, L. D., Ma, X., Yu, F., Liu, Y., et al. (2017). Pollution Condition and the Analysis of Drug Resistance of Foodborne *Staphylococcus aureus* in Xinjiang in 2015. *Farm Products Processing*. (01), 66-68+71. doi: 10.16693/j.cnki.1671-9646(X).2017.01.018.
176. Su, J. H., Zhang, H. H., Fu, H. Q., and Huang, H. (2012). Analysis of 160 serotypes and drug resistance of *Vibrio parahaemolyticus*. *Shanghai J. Prev. Med*. 24(03), 135-138. doi: 10.19428/j.cnki.sjpm.2012.03.011
177. Ta, Y. T., Nguyen, T. T., To, P. B., Pham, D. X., Le, H. T. H., Thi, G. N., et al. (2014). Quantification, Serovars, and Antibiotic Resistance of *Salmonella* Isolated from Retail

Raw Chicken Meat in Vietnam. *J. Food Prot*. 77(1), 57-66. doi: 10.4315/0362-028X.JFP-13-221

178. Tan, C. W., Rukayadi, Y., Hasan, H., Thung, T. Y., Lee, E., Rollon, W. D., et al. (2020). Prevalence and antibiotic resistance patterns of *Vibrio parahaemolyticus* isolated from different types of seafood in Selangor, Malaysia. *Saudi J Biol Sci*. 27(6), 1602-1608. doi: 10.1016/j.sjbs.2020.01.002
179. Tan, C. W., Malcolm, T., Kuan, C. H., Thung, T. Y., Chang, W. S., Loo, Y. Y., et al. (2017). Prevalence and Antimicrobial Susceptibility of *Vibrio parahaemolyticus* Isolated from Short Mackerels (Rastrelliger brachysoma) in Malaysia.*Front. Microbiol*. 8, 1087-. doi: 10.3389/fmicb.2017.01087
180. Tang, J., Ma, G. Z., Huang, X. X., Chen, Y. L., Kong, H. J., Bo Y., et al. (2020). Distribution and drug resistance of food-borne *Salmonella* in Hanzhong. *LAB MED*. 2020, 35(11), 1161-1164.
181. Tang, Y. X. (2017). Isolation and molecular characteristics of *Vibrio parahaemolyticus* in commercially available Tegillarca granosa in Hangu of Binhai New Area of Tianjin. *Occup. Health*. 33(15), 2051-2054. doi: 10.13329/j.cnki.zyyjk.2017.0619
182. Tang, Z., Qiao, X., Qin, S., Shen, Y., Ni, Y. L., Wang, H. Y., et al. (2017). Analysis of contamination status, virulence genes distribution and drug resistance of *Vibrio parahaemolyticus* in aquatic products of Jiangsu province from 2015 to 2017. *Jiangsu J. Prev. Med*. 029(004), 378-381. doi: 10.13668/j.issn.1006-9070.2018.04.005
183. Tang, Z., Yun, S., Si, Q., Zhen, D. Y., Ma, K., Qiao, X., et al. (2018). Infection status and drug resistance of diarrheogenic *Escherichia coli* in foodborne diseases in Jiangsu Province,2017. *Acta Universitatis Medicinalis Nanjing (Natural ence)*. 38(10), 1371-1375. doi: CNKI:SUN:NJYK.0.2018-10-010.
184. Tadesse, H. A., Gidey, N. B., Workelule, K, Hailu, H., Gidey, S., Bsrat, A., et al. (2018). Antimicrobial Resistance Profile of *E. coli* Isolated from Raw Cow Milk and Fresh Fruit Juice in Mekelle, Tigray, Ethiopia. *Vet. Med. Int*. 2018, 1-7. doi: 10.1155/2018/8903142
185. Temelli, S., Kahya, S., Eyigör, A., and Carli, K. T. (2017). Antibiotic Resistance Phenotypes of *Salmonella* Isolates of Chicken Meat and Chicken Origin. *Vet. Fakultesi Dergisi*. 59(2), 107-114. doi: 10.1501/Vetfak_0000002511
186. Torpdahl, M., Lauderdale, T. L., Liang, S. Y., Li, I., Wei, S. H., and Chiou, C. S. (2013). Human isolates of *Salmonella* enterica serovar Typhimurium from Taiwan displayed significantly higher levels of antimicrobial resistance than those from Denmark. *Int. J. Food Microbiol*. 161(2), 69-75. doi: 10.1016/j.ijfoodmicro.2012.11.022
187. Touimi, G. B., Bennani, L., Berrada, S., Moussa, B., and Bennani, B. (2020). Prevalence and antibiotic resistance profiles of Staphylococcus sp. isolated from food, food contact surfaces and food handlers in a Moroccan hospital kitchen. *Lett. Appl. Microbiol*. 70(4), 241-251. doi: 10.1111/lam.13278
188. Uysal, A., and Durak, Y. (2012). Pulsed-field gel electrophoresis typing, antibiotic resistance, and plasmid profiles of *Escherichia coli* strains isolated from foods. *Can J Microbiol*. 58(11), 1278-1287. doi: 10.1139/w2012-108
189. Vega-Sanchez, V., Barba-Leon, J., Gonzalez-Aguilar, D. G., Cabrera-Diaz, E., Pacheco-Gallardo, C., and Orozco-Garcia, A. G. (2020). Antimicrobial resistance in *Salmonella* spp. isolated from pig carcasses in two slaughterhouse types in Jalisco, Mexico. *Rev. Mex.*

Cienc. Pecu. 11(4), 1004-1015. doi: 10.22319/rmcp.v11i4.5386

190. Viana, C., Grossi, J. L., Sereno, M. J., Yamatogi, R. S., Bersot, L. D., Call, D. R., et al. (2020). Phenotypic and genotypic characterization of non-typhoidal *Salmonella* isolated from a Brazilian pork production chain. *Food Res. Int*. 137(6), 109406. doi: 10.1016/j.foodres.2020.109406
191. Wang, D., Zhang, X. A., Chen, Q., Lu, Z., and Wang, L. L. (2014). Study of antimicrobial resistance and molecular characteristics in foodborne *Staphylococcus aureus* in Beijing. *Chinese Journal of Food Hygiene*, 26(05), 428-434. doi: 10.13590/j.cjfh.2014.05.005
192. Wang, G., Wang, M., Cai, Y. M., Tang, X. Z., Ren, X., Zou, Y. Q. S. et al. (2020). Pollution and drug resistance analysis of *Vibrio parahaemolyticus* in freshwater fish in Yunnan. *J. Food Ssf. Food Qual*. 11(09): 2779-2784.
193. Wang, H. L., Tang, X. Y., Su, Y. C., Chen, J. B., and Yan, J. B. (2017). Characterization of clinical *Vibrio parahaemolyticus* strains in Zhoushan, China, from 2013 to 2014. *PLoS ONE*. 12(7), e0180335. doi: 10.1371/journal.pone.0180335
194. Wang, J., He, D. M., Zhu, H. M., Song, M. D., Chen, Q. X., and Lai, W. D. (2013). Etiological analysis of 35 isolates of *Vibrio parahaemolyticus* causing food poisonings. *South China Journal of Preventive Medicine*, 39(06): 24-26+32. doi: 10.13217/j.scjpm.2013.06.024
195. Wang, J. W., Sheng, H. J., Xu, W. L., Huang, J. L., Meng, L. Y., Cao, C. Y., et al. (2019). Diversity of Serotype, Genotype, and Antibiotic Susceptibility of *Salmonella* Prevalent in Pickled Ready-to-Eat Meat. *Front. Microbiol*. 10, 2577. doi: 10.3389/fmicb.2019.02577
196. Wang, L., Jia, H. Y., Zhang, H., Zhang, L. Q., and Hu, H. (2011). Study on Serotype Distribution and Drug Resistance of Foodborne. *Prev. Med*. 18(06), 994-997.
197. Wang, L., Nakamura, H., Kage-Nakadai, E., Hara-Kudo, Y., and Nishikawa, Y. (2017). Prevalence, antimicrobial resistance and multiple-locus variable-number tandem-repeat analysis profiles of diarrheagenic *Escherichia coli* isolated from different retail foods. *Int. J. Food Microbiol*. 249, 44-52. doi: 10.1016/j.ijfoodmicro.2017.03.003
198. Wang, L. L., and Chen, Q. (2016). Molecular subtyping and antibiotic resistance of Listeria monocytogenes isolated from patients in Beijing. *Chinese Journal of Food Hygiene*. 28(04), 426-430. doi: 10.13590/j.cjfh.2016.04.004
199. Wang, L. P., Wu, L., Zhang, B. B., and Wu, Y. H. (2019). Analysis of serotype distribution and antimicrobial resistance of *Salmonella* strains from food in Inner Mongolia Autonomous Region within 2014-2017. *Chinese Journal of Health Laboratory Technology*. 29(23), 2847-2850.
200. Wang, P., Qiang, X. H., and Zhou, L. H. (2017). Etiological analysis of characteristics of *Vibrio parahaemolyticus* in food borne diarrhea patients in Huzhou, Zhejiang. *Chinese Journal of Health Laboratory Technology*, 27(12), 1706-1708. doi: CNKI:SUN:ZWJZ.0.2017-12-011.
201. Wang, Q. Y., Zhu, L. H., Zhang, L. R., Gao, X., Zhang, C., Kan, B., et al. (2012). Analysis of Antibiotics Resistance in *Vibrio parahaemolyticus* Isolated in Tongzhou District, Beijing, 2009 ～ 2011. *Lett. Biotechnol*. 23(03), 333-336. doi: CNKI:SUN:SWTX.0.2012-03-007.

202. Wang, W., Baloch, Z., Jiang, T., Zhang, C. S., Peng, Z. X., Li, F. Q., et al. (2017). Enterotoxigenicity and antimicrobial resistance of *Staphylococcus aureus* isolated from retail food in China.*Front. Microbiol*. 8, 2256-. doi: 10.3389/fmicb.2017.02256
203. Wang, W., Wang, L., Su, J. Y., and Xu, Z. B. (2020). Antibiotic Susceptibility, Biofilm-Forming Ability, and Incidence of Class 1 Integron of *Salmonella* spp., *Escherichia coli*, and *Staphylococcus aureus* Isolated from Various Foods in a School Canteen in China. *Foodborne Pathog. Dis*. 17(4), 269-275. doi: 10.1089/fpd.2019.2694
204. Wang, W., Bi, L., Li, X. L., Li, F. Q., Wang, Y., Li, H., et al. (2020). Antimicrobial resistance， virulence factors and molecular characterization of methicillin-resistant *Staphylococcus aureus* isolates cultured from ready-to-eat foods. *J. Hyg. Res*. 49(01), 56-62. doi: 10.19813/j.cnki.weishengyanjiu.2020.01.010
205. Wang, W., Zhao, X., Zhang, Y. N., Yu-Jie, H. U., Ke, M. A., Wang, Z. G., et al. (2016). Quantitative determination of contamination and antimicrobial resistance of *Salmonella* in chicken carcasses in slaughterhouses in Qingdao. *Chinese Journal of Food Hygiene*. 28(06), 714-719. doi: 10.13590/j.cjfh.2016.06.006.
206. Wang, W. X., Tan, A. J., Lv, S. M., Jin, Z. Q., and Bai, B. (2011). Bactericide Tolerance of Three Species of Foodborne Pathogenic Bacteria Isolated from Pork in Guiyang City. *Journal of Mountain Agriculture and Biology*. 30(02), 141-143+160. doi: 10.15958/j.cnki.sdnyswxb.2011.02.008
207. Wang, X. L., Zhang, M. H., Zhu, L. Q., Tang, Q. Y., and Zou, W. Y. (2020). Virulence gene，drug resistance，molecular typing of *Vibrio parahaemolyticus* in Suzhou, 2016－2019. *Mod. Prev. Med*. 47(21), 3975-3980. doi: 10.19812/j.cnki.jfsq11-5956/ts.2021.16.053.
208. Wang, Y. B., Li, Y., Li, Z., Wang, Y. Y., Yang, J., and Wang, M. (2018). Laboratory Detection of Food-borne Diseases in Shunyi District of Beijing in 2016. *J. Prev. Med. Inf*. 34(03), 344-348. doi: CNKI:SUN:YFYX.0.2018-03-025.
209. Wang, Z. E., Yu, H., Wei, X. X., Shi, J., Qu, M., and Feng, H. R. (2019). Detection and drug resistance of enteric pathogens in Fengtai District in 2017. *Chinese Journal of Health Laboratory Technology*. 29(08), 928-930+936. doi: CNKI:SUN:ZWJZ.0.2019-08-010.
210. Wei, Q., Xiang, L., Zhang, Y. F., Hao, Q., Shen, M., and Tian, X. W. (2016). Serotype distribution and drug resistance of food-born and human orign *Salmonella* in Ningxia. *Chin. J. Antibiot*. 41(9). doi: 10.13461/j.cnki.cja.005770
211. Wieczorek, K., and Osek, J. (2017) Prevalence, genetic diversity and antimicrobial resistance of *Listeria monocytogenes* isolated from fresh and smoked fish in Poland. *Food Microbiol*. 64,164-171. doi: 10.1016/j.fm.2016.12.022
212. Wu, N. W., Li, X., Deng, Y., Mo, L. J., Feng, J. K., Chen, J., et al. (2012). Study on virulence genes and drug resistance of *Vibrio parahemolyticus* isolated from food-poisoning cases in Sanya. *Mod. Prev. Med*. 39(16), 4221-4222+4225. doi: CNKI:SUN:XDYF.0.2012-16-076.
213. Wu, Q. (2015). Correlation analysis of *Vibrio parahaemolyticus* from contaminated aquatic products and clinical infections in Beijing, *Chinese Center for Disease Control and Prevention*. 2015.
214. Wu, S., Zhang, F., Huang, J. H., Wu, Q. P., Zhang, J. M., Dai, J. S., et al. (2019). Phenotypic and genotypic characterization of PVL-positive *Staphylococcus aureus*

isolated from retail foods in China. *Int. J. Food Microbiol*. 304, 119-126. doi: 10.1016/j.ijfoodmicro.2019.05.021

215. Wu, S., Wu, Q. P., Zhang, J. M., Chen, M. T., and Yan, Z. (2015). Prevalence, antibiotic resistance and genetic diversity of *Listeria monocytogenes* isolated from retail ready-to-eat foods in China, *Food control*. 47, 340-347. doi: 10.1016/j.foodcont.2014.07.028
216. Wu, X. F., Zhuang, C. H., Zheng, Y. X., and Chen, Y. Y. (2018). Serotyping and drug-resistance analysis of *Salmonella* isolated from patients with infectious diarrhea in Quanzhou City from 2013 to 2016. *Chinese Journal of Food Hygiene*. 30(04), 368-372. doi: 10.13590/j.cjfh.2018.04.006
217. Xia, D., Wu, X. W., Zhang, X. Q., Liu, Y. H., Jia-Hai, L. U., and Wang, M. (2017). Production of enterotoxin, antimicrobial resistance and pulsed field gel electrophoresis typing of *Staphylococcus aureus* isolated from food poisoning. *J. Trop. Med*. 17(07), 896-899+980. doi: CNKI:SUN:RDYZ.0.2017-07-014.
218. Xie, A. R., Li, Y., Hong, C. J., Shangguan, Z. H., and Yu-Qin, H. U. (2017). Analysis of drug resistance,enterotoxin and its genes study of foodborne *Staphylococcus aureus* isolated from Wenzhou. *Chinese Journal of Health Laboratory Technology*. 27(13), 1872-1875+1878. doi: CNKI:SUN:ZWJZ.0.2017-13-015.
219. Xie, T. F., Wu, Q. P., Zhang, J. M., Xu, X. K., and Cheng, J. H. (2017). Comparison of *Vibrio parahaemolyticus* isolates from aquatic products and clinical by antibiotic susceptibility, virulence, and molecular characterisation. *Food Control*. 71, 315-321. doi: 10.1016/j.foodcont.2016.06.046
220. Xie, T. F., Wu, Q. P., Xu, X. K., Zhang, J., and Guo, W. P. (2015). Prevalence and population analysis of *Vibrio parahaemolyticus*in aquatic products from South China markets. *FEMS MICROBIOL LETT*. 326(22). doi: 10.1093/femsle/fnv178
221. Xie, T. F., Xu, X. K., Wu, Q. P., Zhang, J., and Cheng, J. (2016). Prevalence, Molecular Characterization, and Antibiotic Susceptibility of *Vibrio parahaemolyticus* from Ready-to-Eat Foods in China.*Front. Microbiol*. 7. doi: 10.3389/fmicb.2016.00549
222. Xing, Y., Qian, Y. C., Hu, Z. W., and Zhang, W. Y. (2016). Analysis of serotype distribution and antimicrobial resistance of 75 *Salmonella* strains isolated from clinic patients in Hefei. *Anhui Journal of Preventive Medicine*. 22(05), 291-293+329. doi: CNKI:SUN:AHYF.0.2016-05-001.
223. Xu, B. (2017). Serological types and drug resistance research of *Vibrio parahaemolyticus* in gastrointestinal clinic. *Chinese Journal of Health Laboratory Technology*. 27(08), 1185-1186. doi: CNKI:SUN:ZWJZ.0.2017-08-040.
224. Xu, F. F., Cai, Y., Jin, T., Jing-Ye, X. U., and Sang, Q. F. (2011). An analysis over the results of *Vibrio parahaemolyticus* monitor of infectious diarrhea in Ningbo Jiangbei district from 2007 to 2010. *Chinese Journal of Health Laboratory Technology*. 21(04), 973-975. doi: CNKI:SUN:ZWJZ.0.2011-04-078.
225. Xu, H. Y., Zhang, W., Guo, C., Xiong, H. P., Chen, X., Jiao, X. N., et al. (2019). Prevalence, Serotypes, and Antimicrobial Resistance Profiles Among Salmonella Isolated from Food Catering Workers in Nantong, China. *Foodborne Pathog. Dis*. 16(5), 346-351. doi: 10.1089/fpd.2018.2584

226. Xu, J., Shi, C., Song, M. H., Xu, X. B., Yang, P. Y., Paoli, G., et al. (2014). Phenotypic and genotypic antimicrobial resistance traits of foodborne Staphylococcus aureus isolates from Shanghai. *J. Food Sci*. 79(4), M635-M642. doi: 10.1111/1750-3841.12405.
227. Xu, J. Y., Shen, X. Y., and Yang, Y. B. (2015). Etiological surveillance and analysis of diarrhea of foodborne bacterial pathopens in Ningbo city. *Chinese Rural Health Service Administration*. 35(04), 469-473. doi: CNKI:SUN:ZNWS.0.2015-04-027.
228. Xu, J. Y., Yan, P., Yang, Y. B., Zhang, D. Y., and Rong-Hua, H. U. (2015). Inspection of pathogenic bacteria in food and analysis of epidemic strains in Ningbo. *Chinese Journal of Food Hygiene*. 27(05), 562-568. doi: 10.13590/j.cjfh.2015.05.018.
229. Xu, Q. F., Lv, X. Y., Zhang, X. M., Shi, Y. R., Gao, H. M., Xu, X. X., et al. (2020). Distribution and drug resistance analysis of *Vibrio parahaemolyticus* in Qingpu District of Shanghai in 2015－2016. *Journal of Medical Pest Control*. 36(03), 248-250. doi: CNKI:SUN:YXDZ.0.2020-03-013.
230. Xu, X. K., Cheng, J. H., Wu, Q. P., Zhang, J. M., and Xie, T. F. (2016). Prevalence, characterization, and antibiotic susceptibility of *Vibrio parahaemolyticus* isolated from retail aquatic products in North China. *Bmc. Microbiol*. 2016, 16(1), 32. doi: 10.1186/s12866-016-0650-6
231. Xu, Z. H., Wang, M., Zhou, C. Y., Gu, G. M., Liang, J. Z., Hou, X. J., et al. (2020). Prevalence and antimicrobial resistance of retail-meat-borne *Salmonella* in southern China during the years 2009–2016: The diversity of contamination and the resistance evolution of multidrug-resistant isolates. *Int. J. Food Microbiol*. 333. doi: 10.1016/j.ijfoodmicro.2020.108790
232. Yan, H., Neogi, S. B., Mo, Z., Guan, W. Y., Shen, Z. X., Zhang, S. H., et al. (2010). Prevalence and characterization of antimicrobial resistance of foodborne *Listeria monocytogenes* isolates in Hebei province of Northern China, 2005-2007. *Int. J. Food Microbiol*. 144(2), 310-316. doi: 10.1016/j.ijfoodmicro.2010.10.015.
233. Yan, H. Q., Huang, Y., Bing, L. V., Zhang, X., Pan, B., and Mei, Q. U. (2015). Analysis of the antimicrobial resistance of Vibrio parahaemolytics from different sources. *Chinese Journal of Health Laboratory Technology*. 25(21), 3765-3768+3771. doi: CNKI:SUN:ZWJZ.0.2015-21-062.
234. Yan, S. F., Pei, X. Y., Yang, D. J., Dong-Min, Y. U., Gan, X., Wang, W., et al. (2014). Antibiotic resistance and MLST analysis of foodborne pathogenic *Listeria Monocytogenes* in China in 2012. *Chinese Journal of Food Hygiene*. 26(06), 537-542. doi: 10.13590/j.cjfh.2014.06.005.
235. Yang, H., Ma, Y., Liu, X. T., Lv, W. T., Lu, L. Z., and Xiao, Y. P. (2020). Virulence genes and drug resistance characteristics of Escherichia coli in ready-to-eat duck products. *Acta Agriculturae Zhejiangensis*. 32(10), 1841-1848.
236. Yang, J., Ma, Z. L., and Cai, Z. (2016). Analysis on the contamination and drug resistance of *Vibrio parahaemolyticus* in 112 aquatic products in Taizhou. *Mod. Prev. Med*. 43(20), 3699-3702. doi: CNKI:SUN:XDYF.0.2016-20-015.
237. Yang, J. X., Zhang, Z. F., Zhou, X. J., Cui, Y., and Shi, X. (2019). Prevalence and Characterization of Antimicrobial Resistance in Salmonella enterica Isolates from Retail Foods in Shanghai, China. *Foodborne Pathog. Dis*. 17(1). doi: 10.1089/fpd.2019.2671

238. Yang, L. H., Chen, M., Chen, H. Y., Xue-Bin, X. U., Zhang, X., Wang, X. G., et al. (2010). Surveillance and PFGE analysis on *Vibrio parahaemolyticus* in agricultural products in Minhang. *Chinese Journal Of Health Laboratory Technology*. 20(07), 1611-1614. doi: CNKI:SUN:ZWJZ.0.2010-07-011.
239. Yang, X. J., Wu, Q. P., Zhang, J. M., Huang, J., and Wei, X. (2019). Prevalence, Bacterial Load, and Antimicrobial Resistance of *Salmonella* Serovars Isolated From Retail Meat and Meat Products in China. *Front. Microbiol*. 2019, 10:2121-. doi: 10.3389/fmicb.2019.02121
240. Yang, X. J., Huang, J. H., Wu, Q. P., Zhang, J., Liu, S. R., Guo, W. P., et al. (2016). Prevalence, antimicrobial resistance and genetic diversity of *Salmonella* isolated from retail ready-to-eat foods in China. *Food Control*. 60, 50-56. doi: 10.1016/j.foodcont.2015.07.019
241. Yang, X. W., Kuang, D., Meng, J. H., Pan, H.J ., Shen, J. Q., Zhang, J., et al. (2015). Antimicrobial Resistance and Molecular Typing of *Salmonella* Stanley Isolated from Humans, Foods, and Environment. *Foodborne Pathog. Dis*. 12(12), 945. doi: 10.1089/fpd.2015.2010
242. Yang, Y., Xie, J. F., Li, H., Tan, S. W., Chen, Y. F., and Yu, H. (2017). Prevalence, Antibiotic Susceptibility and Diversity of *Vibrio parahaemolyticus* Isolates in Seafood from South China. *Front. Microbiol*. 8, 2566. doi: 10.3389/fmicb.2017.02566
243. Yang, Y. J. (2015). Research on pulsed-field gel electrophoresis typing, virulent genes and drug resistance in *Vibrio parahaemolyticus* isolates from partial aquatic products in Kaifeng. *Chinese Journal of Health Laboratory Technology*. 25(21), 3691-3693+3699. doi: CNKI:SUN:ZWJZ.0.2015-21-035.
244. Yano, Y., Hamano, K., Satomi, M., Tsutsui, I., Ban, M., and Aue-Umneoy, D. (2014). Prevalence and antimicrobial susceptibility of Vibrio species related to food safety isolated from shrimp cultured at inland ponds in Thailand. *Food Control*. 38(4), 30-36. doi: 10.1016/j.foodcont.2013.09.019
245. Yao, Y., Lu, Y., and Zhang, M. Q. (2019). Pathogen distribution and drug resistance analysis of foodborn diseases in Changshan County, Zhejiang Province. *Chinese Journal of Health Laboratory Technology*. 29(23), 2900-2902. doi: CNKI:SUN:ZWJZ.0.2019-23-029.
246. Li, Z. Y., Yin, B. K., Pan, Z. W., and Zheng, N. C. (2012). Pollution status and resistance of Staphylococcus aureus in food of urban area of Jiangmen City. *Occup. Health*. 28(18), 2263-2265. doi: 10.13329/j.cnki.zyyjk.2012.18.037.
247. Yim, J. H., Kim, K. Y., Chon, J. W., Kim, D. H., Kim, S. H., Choi, D. S., et al. (2015). Incidence, Antibiotic Susceptibility, and Toxin Profiles of Bacillus cereus sensu lato Isolated from Korean Fermented Soybean Products. *J. Food Sci*. 80(6), M1266-M1270. doi: 10.1111/1750-3841.12872
248. Yoon, R. H., Cha, S. Y., Bai, W., Roh, J. H., Seo, H. S., Jae-Young, O. H., et al. (2014). Prevalence of *Salmonella* isolates and antimicrobial resistance in poultry meat from South Korea. *J. Food Protect*. 77(9), 1579-1582. doi: 10.4315/0362-028X.JFP-14-018
249. You, X. Y., Peng, S. L., Zhou, H. D., Liu, Y., Liu, D. F., Hu, K., et al. (2020). Analysis of nontyphoidal Salmonella clinical isolates antibiotic resistance based on whole genome

sequencing in Jiangxi Province in 2018. *Chinese Journal of Food Hygiene*. 5, 493-498. doi: 10.13590/j.cjfh.2020.05.004.

250. Yu, J. M., Ni, J. J., Tao, D. Y., Lao, H. J., Wang, J. F., and Wang, C. (2014). Survey of drug resistance and virulence gene of *Vibrio parahaemolyticus* from imported aquatic products. *Chinese Journal of Food Hygiene*. 26(04), 320-323. doi: 10.13590/j.cjfh.2014.04.004.
251. Yu, P. F., Yu, S. B., Wang, J., Guo, H. Zhang, Y. Liao, X. Y., et al. (2019). Bacillus cereus Isolated From Vegetables in China: Incidence, Genetic Diversity, Virulence Genes, and Antimicrobial Resistance.*Front. Microbiol*. 10. doi: 10.3389/fmicb.2019.00948
252. Yu, Q. Q., Niu, M. Y., Yu, M. Q., Liu, Y. H., Wang, D. P., Shi, and X. M., (2016). Prevalence and antimicrobial susceptibility of *Vibrio parahaemolyticus* isolated from retail shellfish in Shanghai. *Food control*. 60, 263-268. doi: 10.1016/j.foodcont.2015.08.005
253. Yuan, D. K., Zhang, L. P., Huang, S. Z., Kang, M. X., and Mo, X. M. (2013). Serotype and drug-resistance of 79 *Vibrio parahaemolyticus* strains in Dongguan. *Chinese Journal of Health Laboratory Technology*. 23(13). doi: CNKI:SUN:ZWJZ.0.2013-13-043.
254. Yuan, M., Li, Z., Zhao , X., Chen, Z. G., and Cheng, L. (2014). Analysis of drug resistance of food-borne *Escherichia coli* in Nanyang region of Henan Province. *World Chin. J. Digestol*. 22(23), 3459-3463. doi: CNKI:SUN:XXHB.0.2014-23-019.
255. Yuan, P. Z., Wang, X. L, Luo, S. B., and Chuan-Da, L. I. (2016). Analysis of foodborne *Salmonella* surveillance and drug resistance in Neijiang area during 2012-2014. *Lab. Med. Clin*. 13(01), 4-5. doi: CNKI:SUN:JYYL.0.2016-01-002.
256. Yuan, Y., Shun, Y., Zhang, J., Chun, L. I., Nan, S. A., Wei-Wei, L. I., et al. (2013). Research on the pathogenic features of *Vibrio parahaemolyticus* in instant sea foods from Hefei market. *Anhui Journal of Preventive Medicine.* 19(4). doi: CNKI:SUN:AHYF.0.2013-04-000.
257. Yuan, Z. H., Zheng, K. Y., Liu, Q. M., Qu, J. J., Qiu, Q. L., and Ye, Z. Y. (2019). Characteristics of drug resistance and molecular typing of *Salmonellas* isolated in Zhongshan, Guangdong in 2017. *China Trop. Med*. 19(5). doi: 10.13604/j.cnki.46-1064/r.2019.05.13
258. Yue, M., Li, X., Liu, D., and Hu, X. (2020). Serotypes, antibiotic resistance, and virulence genes of *Salmonella* in children with diarrhea. *J CLIN LAB ANAL*. 34(12). doi: 10.1002/jcla.23525
259. Zanella, G. N., Mikcha, J. M. G., Bando, E., Siqueira, V. L. D., and Machinski, M. (2010). Occurrence and Antibiotic Resistance of Coliform Bacteria and Antimicrobial Residues in Pasteurized Cow's Milk from Brazil. *J. Food Protect*. 73(9), 1684-1687. doi: 10.4315/0362-028X-73.9.1684
260. Zeng, X, Y,, Lu, S. L., Du, Y., Wei, C. Y., Cong, Q. U., and Xiu-Gui, L. I. (2018). Antibiotic resistance of foodborne *Salmonella* isolates in Guangxi in 2016. *Chinese Journal of Food Hygiene*. 30(01), 22-27. doi: 10.13590/j.cjfh.2018.01.005.
261. Zeng, Y. B., Xiong, L. G., Tan, M. F., Li, H. Q., Yan, H., Zhang, L., et al. (2019). Prevalence and Antimicrobial Resistance of Salmonella in Pork, Chicken, and Duck from Retail Markets of China. *Foodborne Pathog. Dis*. 16(5) 339-345. doi: 10.1089/fpd.2018.2510

262. Zhang, J., Chen, H. L., Deng, Z. A., Luo, J. M., Liu, Q. Y., Xin-Wei, W. U.,et al. (2018). Enterotoxins and drug resistance of foodborne *Staphylococcus aureus* in Guangzhou City. *Pract. Prev. Med*. 25(04), 398-400. doi: CNKI:SUN:SYYY.0.2018-04-004.
263. Zhang, J. Q., Miao, C., and Chu, J. H. (2020). Analysis of active surveillance results of specific pathogens of foodborne diseases in Yuyao City. *Chinese Journal of Health Laboratory Technology*. 30(15), 1892-1895. doi: CNKI:SUN:ZWJZ.0.2020-15-030.
264. Zhang, J. Q., Luo, X. H., He, S. Y., and Zhang, Y. M. (2010). Monitoring and research of infectious diarrhea in *Vibrio parahaemolyticus*. *Chinese Journal of Health Laboratory Technology*. 20(02), 376-378. doi: CNKI:SUN:ZWJZ.0.2010-02-060.
265. Zhang, L. H., Zhu, X. H., Guo, Z. S., Zhang, L., and Chen, H. L. (2014). Investigation on the molecular characteristics and drug resistance of nontyphoidal *Salmonella* isolates from commercial chickens and diarrhea patients. *Chinese Journal of Food Hygiene*. 26(06), 605-609. doi: 10.13590/j.cjfh.2014.06.022.
266. Zhang, M. Y., Geng, Y. Z., Yu, M., and Zhang, M. M. (2020). Drug resistance analysis of *Salmonella* isolated from different sources in Liaoning province. *J. Food Ssf. Food Qual*. 11(19),7142-7146. doi: 10.19812/j.cnki.jfsq11-5956/ts.2020.19.072.
267. Zhang, S. H., Wu, Q. P., Zhang, J. M., Lai, Z. B., and Zhu, X. M. (2016). Prevalence, genetic diversity, and antibiotic resistance of enterotoxigenic *Escherichia coli* in retail ready-to-eat foods in China. *Food Control*. 68, 236-243. doi: 10.1016/j.foodcont.2016.03.051
268. Zhang, S. H., Hou, F. L., Guan, W. Y., and Shen, Z. X. (2014). Characteristic analyses of prevalence and antimicrobial resistances of *Listeria monocytogenes* in ready-to-eat foods in Hebei Province from 2005 to 2013. *Chinese Journal of Food Hygiene*. 6, 596-599. doi: 10.13590/j.cjfh.2014.06.020.
269. Zhang, X. A., Liu, Y. Z., Zhang, P. H., Wang, D., Ma, X. C., and Chen, Q. (2020). Molecular characteristics and drug resistance of foodborne *Listeria monocytogenes* in Beijing in 2019. *J. Food Ssf. Food Qual*. 11(24), 9343-9348. doi: 10.19812/j.cnki.jfsq11-5956/ts.2020.24.047.
270. Zhang, Y., Chen, M. F., Yu, P. F., Yu, S. B., Wang, J., Guo, H., et al. (2020). Prevalence, Virulence Feature, Antibiotic Resistance and MLST Typing of Bacillus cereus Isolated From Retail Aquatic Products in China. *Front. Microbiol*. 11, 1513. doi: 10.3389/fmicb.2020.01513
271. Zhang, Y., Chen, X. Q., and Song, C. Y. (2011). Foodborne *Salmonella* Serotype Distribution and Sensitivity Analysis in Shanghai in 2010. *Prog. Mod. Biomed*. 11(20), 3938-3941. doi: 10.13241/j.cnki.pmb.2011.20.010.
272. Zhang, Y. Y., Dong, S. L., Chen, H. H., Chen., J. C., Zhang, J. Y., Zhang, Z., et al. (2019). Prevalence, Genotypic Characteristics and Antibiotic Resistance of *Listeria monocytogenes* From Retail Foods in Bulk in Zhejiang Province, China. *Front. Microbiol*. 10, 1710. doi: 10.3389/fmicb.2019.01710
273. Zhang, Z. F., Cao, C. Y., Liu, B., Xu, X. B., Yan, Y. F.,Cui, S. H., et al. (2018). Comparative Study on Antibiotic Resistance and DNA Profiles of *Salmonella* enterica Serovar Typhimurium Isolated from Humans, Retail Foods, and the Environment in Shanghai, China. *Foodborne Pathog. Dis*. 15(8), 481-488. doi: 10.1089/fpd.2017.2414

274. Zhang, Z. Y., Liu, J., Dong, J., and Xu, S. U. (2015). Investigation on the contamination and drug resistance of *Vibrio parahaemolyticus* in shellfishes sold in Tianjin. *Chinese Journal of Health Laboratory Technology*. 25(14), 2413-2415. doi: CNKI:SUN:ZWJZ.0.2015-14-053.
275. Zhao, D., Pei, M. J., Zhang, W. L., Liang, R., Xiao, N., and Tang, Y. T. (2010). Analysis of resistance and virulence genes of foodborne *Staphylococcus aureus* in northwestern Hubei Province. *Chinese Journal of Food Hygiene*. 32(06), 620-625. doi: 10.13590/j.cjfh.2020.06.006.
276. Zhao, S. J., Chen, J. L., Fei, P., Feng, H. X., Wang, Y., Ali, M. A., et al. (2020). Prevalence, molecular characterization, and antibiotic susceptibility of Bacillus cereus isolated from dairy products in China. *J. Dairy. Sci*. 103(5), 3994-4001. doi: 10.3168/jds.2019-17541
277. Zhao, Y., Fu, P., Pei, X. Y., Gang, W., and Guo, Y. (2012). Characteristic analysis of antimicrobial resistance of *Listeria monocytogenes* in foods. *Chinese Journal of Food Hygiene*. 24(01), 5-8. doi: 10.13590/j.cjfh.2012.01.015.
278. Zhen, Z. B., Zhen, W., Wang, H. Q., Yu, Y., Pan, J. C., Chen, Q., et al. (2021). Characteristics and molecular subtyping of multidrug resistant *Salmonella* in Hangzhou. *Microbiol. China*. 48(02) 536-544. doi: 10.13344/j.microbiol.china.200151
279. Zheng, W. L., Dong, J., Wang, Z., Zhao, S., Ma, J., and Wang, G. H. (2015). A study of the epidemiological features of diarrheas caused by *Vibrio parahaemolyticus* and the antibiotic resistance spectrum analysis in Tianjin City. *Chinese Journal of Disease Control and Prevention*. 19(09), 963-965. doi: 10.16462/j.cnki.zhjbkz.2015.09.027
280. Zheng, W. L., Wang, Z., Dong, J., Ma, J., Zhao, S., and Wang, G. H. (2015). *Vibrio parahaemolyticus* contamination and pathogenicity in aquatic products in Tianjin: a cross-sectional study. *J. Environ. Sci. Health*. 32(06), 540-543. doi: 10.16241/j.cnki.1001-5914.2015.06.020
281. Zhou, H. D., Liu, D., Peng, S. L., Liu, D. F., You, X. Y., Liu, Y., et al. (2018). Analysis of molecular type and drug resistance of food-borne *Salmonella* in Jiangxi province from 2016 to 2017. *Chin. J. Disinfect*. 35(10), 764-769. doi: CNKI:SUN:ZGXD.0.2018-10-015.
282. Zhou, H. Z., Li, Y., Zong, C. Y., and Zhou, T. T. (2010). The investigation and analysis on contamination and drug resistance of *Vibrio parahaemolyticus* in small aquatic products in a large supermarket in Suzhou,Jiangsu province. *China Anim. Health Insp*. 27(12): 59-60. doi: CNKI:SUN:ZGDW.0.2010-12-026.
283. Zhou, Q. (2016). Study on the contamination and drug resistant status of *Vibrio parahaemolyticus* in guiyang aquatic products. *Guizhou Medical University.*
284. Zhou, Q., Zhou, L., Tian, P., and Xiang, J. S. (2019). Virulent gene profiles and antimicrobial resistance of foodborne *Bacillus cereus*, Guizhou. *Mod. Prev. Med*. 46(11), 2019-2023. doi: CNKI:SUN:XDYF.0.2019-11-024.
285. Zhou, W. Y., Zheng, J., Ye, S., Shen, X. Y., Yang, Y. B., and Xu, J. Y. (2016). Epidemic analysis and detection in diarrhea patients of food borne bacterial pathogens in Ningbo during 2011-2014. *Chinese Journal of Health Laboratory Technology*. 26(15), 2235-2239. doi: CNKI:SUN:ZWJZ.0.2016-15-035.
286. Zhou, Y., Zhang, X. Q., Hou, S. P., Zhang, J., Zhang, Y. S., Tao, X., et al. (2014). Serotype distribution and drug resistance of 76 *Salmonella* isolates in Guangzhou. *Mod. Prev. Med*. 41(02), 349-350+352. doi: CNKI:SUN:XDYF.0.2014-02-052.

287. Zou, Y. Q. S., Yang, Z. S., Tian, Y. P., Guo, Y. D., Ren, X., Tang, X.Z., et al. (2019). Drug resistance analysis of food-borne *Salmonella* in Yunnan from 2014 to 2018. *J. Food Ssf. Food Qual*. 10(22), 7601-7605. doi: 10.19812/j.cnki.jfsq11-5956/ts.2019.22.028.
288. Zhu, A. H., Zhi, W., Qiu, Y. F., Wei, L. L., Tian, J., Pan, Z. M., et al. (2019). Surveillance study of the prevalence and antimicrobial resistance of *Salmonella* in pork from open markets in Xuzhou, China. *Food Control*. 98, 474-480. doi: 10.1016/j.foodcont.2018.07.035
289. Zhu, J. H., Luo, X. H., Zhang, J. Q., and Huang, S. J. (2020). Surveillance results analysis of food-borne pathogen in Yuyao City. *Chinese Journal of Health Laboratory Technology*. 30(07), 884-887. doi: CNKI:SUN:ZWJZ.0.2020-07-033.
290. Zhu, X. J., Wu, X. F., Xu, D. S., Chen, L. P., Ji, L., Shen, Y. H., et al. (2017). Serotyping and drug resistance of *Vibrio parahaemolyticus* in Huzhou City,2013-2015. *Pract. Prev. Med*. 24(09), 1058-1060. doi: 10.3969/j.issn.1006-3110.2017.09.009.
291. Zhu-Ge, S. Y., Su, A. R., and Li, X. G. (2014). Research on the contamination distribution and drug resistance of *Bacillus cereus* in rice and wheaten food in Guangxi. *Chinese Journal of Health Laboratory Technology*. 24(18), 2661-2662+2672. doi: CNKI:SUN:ZWJZ.0.2014-18-024.
292. Zhu-Ge, S. Y., Su, A. R., and Li, X. G. (2015). Research on *Staphylococcus aureus* enterotoxin property and drug resistance in quick frozen flour product in Guangxi. *Mod. Prev. Med*. 42(10), 1765-1767.
293. Zhou, X. H., Sun, M. H., Xu, P. H., Xu, J. Y., Ni, C. M., and Chen, M. F. (2010). Contamination state of *Salmonella* in raw meat products and analysis of its drug resistance. *Chinese Journal of Health Laboratory Technology*. 20(12), 3425-3427. doi: CNKI:SUN:ZWJZ.0.2010-12-136.
294. Beshiru, A., and Igbinosa, E. O. (2018). Characterization of extracellular virulence properties and biofilm-formation capacity of *Vibrio species* recovered from ready-to-eat (RTE) shrimps. *Microb. Pathogenesis*. 119, 93-102. doi: 10.1016/j.micpath.2018.04.015
295. Beshiru, A., Igbinosa, I. H., and Igbinosa, E. O. (2018). Biofilm formation and potential virulence factors of *Salmonella* strains isolated from ready-to-eat shrimps. *PloS One*. 13(9), e0204345. doi: 10.1371/journal.pone.0204345
296. Chen, Q., Xie, S. M., Lou, X. Q., Cheng, S., Liu, X. D., Zheng, W., et al. (2020). Biofilm formation and prevalence of adhesion genes among *Staphylococcus aureus* isolates from different food sources. *Microbiologyopen*. 2020, 9(1), e946. doi: 10.1002/mbo3.946
297. Kim, H. J., Oh, T., and Baek, S. Y. (2018). Multidrug resistance, biofilm formation, and virulence of *Escherichia coli* isolates from commercial meat and vegetable products. *Foodborne Pathog. Dis*. 15(12), 782-789. doi: 10.1089/fpd.2018.2448
298. Lopez-Leon, P., Luna-Gonzalez, A., Escamilla-Montes, R., Flores-Miranda, M. D., Fierro-Coronado, J. A., Alvarez-Ruiz, P., et al. (2016). Isolation and characterization of infectious *Vibrio parahaemolyticus*, the causative agent of AHPND, from the whiteleg shrimp (*Litopenaeus vannamei*). *Lat. Am. J. Aquat. Res*. 44(3), 470-479. doi: 10.3856/vol44-issue3-fulltext-5
299. Maia, D. S. V., Haubert, L., Kroning, I. S., Soares, K. D., Oliveira, T. L., and da Silva, W. P. (2020). Biofilm formation by *Staphylococcus aureus* isolated from food poisoning outbreaks and effect of Butia odorata Barb. Rodr. Extract on planktonic and biofilm cells.

Lwt-Food Sci. Technol. 117, 108685. doi: 10.1016/j.lwt.2019.108685

300. Ou, C. J., Shang, D. Q., Yang, J. X., Chen, B., Chang, J., Jin, F. N., et al. (2020). Prevalence of multidrug-resistant *Staphylococcus aureus* isolates with strong biofilm formation ability among animal-based food in Shanghai. *Food Control*. 112, 107106. doi: 10.1016/j.foodcont.2020.107106
301. Puah, S. M., Tan, J. A. M. A., Chew, C. H., and Chua, K. H. (2018). Diverse profiles of biofilm and adhesion genes in *Staphylococcus aureus* food strains isolated from sushi and sashimi. *J. Food Sci*. 83(9), 346-351. doi: 10.1111/1750-3841.14300
302. Rodriguez-Lazaro, D., Alonso-Calleja, C., Oniciuc, E, A., Capita, R., Gallego, D., Gonzalez-Machado, C., et al. (2018). Characterization of biofilms formed by foodborne methicillin-resistant *Staphylococcus aureus*. *Front. Microbiol*. 9, 3004. doi: 10.3389/fmicb.2018.03004
